# Typing of *Echinococcus multilocularis* by Region-Specific Extraction and Next-Generation Sequencing of the mitogenome

**DOI:** 10.3389/fmicb.2025.1535628

**Published:** 2025-02-28

**Authors:** Franziska Rachel, Christine Luttermann, Dirk Höper, Franz Josef Conraths, Johannes Dapprich, Pavlo Maksimov

**Affiliations:** ^1^National Reference Laboratory for Echinococcosis, Institute of Epidemiology, Friedrich-Loeffler-Institut – Federal Research Institute for Animal Health (FLI), Greifswald, Germany; ^2^Department of Biology, Faculty of Mathematics and Natural Sciences, University of Greifswald, Greifswald, Germany; ^3^Laboratory for Antiviral Immunity, Institute of Immunology, Friedrich-Loeffler-Institut – Federal Research Institute for Animal Health (FLI), Greifswald, Germany; ^4^Laboratory for NGS-Based Pathogen Characterization and Animal Disease Diagnostics, Institute of Diagnostic Virology, Friedrich-Loeffler-Institut – Federal Research Institute for Animal Health (FLI), Greifswald, Germany; ^5^Generation Biotech, Princeton, NJ, United States

**Keywords:** *Echinococcus multilocularis*, mitogenome, mtDNA, Region-Specific Extraction method, RSE, NGS, SNP

## Abstract

**Background:**

Infection by the fox tapeworm *Echinococcus multilocularis* may lead to a severe zoonosis in humans, alveolar echinococcosis, which may be fatal if left untreated. Typing is important to understand the epidemiology of this parasite, yet there is limited knowledge on the microdiversity of *E. multilocularis* on the local scale, since the typing resolution of established methods is restricted.

**Methods:**

The mitogenome of *E. multilocularis* was used as the target regions to modify, apply and validate the Region-Specific Extraction (RSE) method in combination with Next-Generation Sequencing (NGS). Single Nucleotide Polymorphisms (SNPs) were detected in the mitochondrial DNA (mtDNA) and analysed bioinformatically. To validate the success and the accuracy of the RSE protocol, the mitogenomes of some *E. multilocularis* isolates were also analysed by the Whole-Genome Sequencing (WGS).

**Results:**

With the chosen combination of methods, the entire mitogenome (~13 kb) of *E. multilocularis* could be captured and amplified. The read depth (median ≥ 156X) was sufficient to detect existing SNPs. The comparison of mitogenome sequences extracted by RSE with mitogenome sequences obtained by WGS showed that the accuracy of the RSE method was consistently comparable to direct Whole-Genome Sequencing.

**Conclusion:**

The results demonstrate that the RSE method in combination with NGS is suitable to analyse the microdiversity of *E. multilocularis* at the whole mitogenome level. For the capture and sequencing of large (several kb) genomic regions of *E. multilocularis* and other applications, this method can be very helpful.

## 1 Introduction

*Echinococcus* (*E.*) *multilocularis* (Leuckart, 1863; Vogel, 1957; Vuitton et al., 2020) is regarded as one of the most dangerous endoparasites in the northern hemisphere (Conraths et al., 2017; Deplazes et al., 2017; Thompson, 2017; Vuitton et al., 2020) causing alveolar echinococcosis (AE) in humans, which may be fatal if left untreated (Vuitton et al., 2015). Alveolar echinococcosis is classified as one of 20 neglected tropical diseases by the WHO (Bodimeade et al., 2019; Baumann, 2020). Likewise, *E. multilocularis* was included by FAO/WHO in a ranking of the most relevant food-borne pathogens in Europe and is one of the most important of them (Torgerson et al., 2020; van der Giessen et al., 2021). The life cycle is diheteroxenious and consists of a definitive host in Europe mainly the Red Fox (*Vulpes vulpes*), but also domestic dogs (*Canis lupus familiaris*), and the Arctic Fox (*Vulpes lagopus*) on Svalbard as well as the Raccoon Dog (*Nyctereutes procyonoides*), a neozoon at least in most parts of Europe, and a wide range of intermediate hosts including rodents (mainly Arvicolinae) as the main intermediate hosts (Eckert et al., 2001; Henttonen et al., 2001; Vuitton et al., 2003; Kapel et al., 2006; Romig et al., 2006; Dyachenko et al., 2008; Knapp et al., 2012; Chaignat et al., 2015; Conraths and Deplazes, 2015; Bagrade et al., 2016; Romig et al., 2017; Lucius et al., 2018; Conraths and Maksimov, 2020; Kjær et al., 2021; Umhang et al., 2021b; Woolsey and Miller, 2021; Pilarczyk et al., 2022). Humans (but also domestic pigs, wild boar, and horses) represent dead-end intermediate hosts (EFSA, 2015; Conraths and Maksimov, 2020; Vuitton et al., 2020; Gottstein and Deplazes, 2021). They are infected by faecal-oral ingestion of viable eggs excreted by definitive hosts (Deplazes et al., 2011; Woolsey and Miller, 2021).

The entirety of the mitochondrial DNA (mtDNA) is also referred to as the mitogenome [or more rarely as the chondriome (Zardoya, 2020)]. The mtDNA has been widely used as a target region for species identification (Paijmans et al., 2013; Zardoya, 2020; Formaggioni et al., 2021). The mitochondrion is most likely the result of endosymbiosis of a member of the Alphaproteobacteria with its host cell (Gray et al., 1999; Gray, 2012; Zardoya, 2020; Formaggioni et al., 2021; Raval et al., 2022). The mitochondrion therefore has its own DNA, which has adapted to the host in the course of evolution and is therefore smaller, but nevertheless represents a suitable target for species determination (Boore, 1999; Lang et al., 1999; Nakao et al., 2002; Roger et al., 2017; Zardoya, 2020).

The mitogenome of *E. multilocularis* is a single circular DNA molecule comprising of 13,738 bp (Nakao et al., 2002). It contains genes that are important for the respiratory chain (oxidative phosphorylation) and thus for the energy production [Adenosine 5′-(tetrahydrogen triphosphate) or short ATP] of the cells, as well as genes for the subunits of the ribosomes (Nakao et al., 2002). These include, for example, the genes *nad*1 (NADH dehydrogenase subunit 1), *atp*6 (ATPase subunit 6), *cox*1 (cytochrome c oxidase subunit 1), and *rrnS* (small sub-unit of ribosomal RNA) (Boore, 1999; Nakao et al., 2000; Nakao et al., 2002; Trachsel et al., 2007; Zardoya, 2020) which are also target regions for the diagnosis and typing of *Echinococcus* spp. (Herzig et al., 2021).

For genotyping, Single Nucleotide Polymorphisms (SNPs) are commonly used (Brumfield et al., 2003; Morin et al., 2004; Helyar et al., 2011), which represent differences at a single nucleotide position in a DNA sequence between individual genomes of a species (Morin et al., 2004; van Dijk et al., 2014; Nordheim and Knippers, 2018). Genotyping by SNP detection is regarded at least as powerful as the commonly used microsatellite technology (Helyar et al., 2011; Flanagan and Jones, 2019). NGS technology is increasingly used to characterise genotypes using SNPs (Bruijns et al., 2018). However, whole genome sequencing is too expensive for routine diagnostics. Moreover, data processing and analysis, particularly in the case of *E. multilocularis*, are time-consuming, demand significant computing resources, and require specialised bioinformatic skills and equipment. Methods like Region-Specific Extraction (RSE) method, which produces long reads make it possible to characterise long contiguous sequences (Dapprich et al., 2016; Kinkar et al., 2019), so that errors in bioinformatic evaluation can be reduced. As a result, nucleotide sequence-based differences are captured and identified more precisely at a localised scale. Therefore, the combination of SNP typing with RSE offered a chance to sequence the entire mitogenome of *E. multilocularis* for different purposes (Nakao et al., 2002; Laurimäe et al., 2018; Zhao et al., 2022; Bohard et al., 2023). It may enable fingerprinting of individual *E. multilocularis* genotypes and can help to differentiate genotypes also in relation to other characteristics, perhaps even virulence (Šnábel et al., 2020).

While adult *Echinococcus* spp. parasites could only be differentiated morphologically in the past, it is now possible to recognise *E. multilocularis* with the help of molecular methods based on genetic markers (Nakao et al., 2009; Ito et al., 2013; Konyaev et al., 2013; Lymbery, 2017; Šnábel et al., 2020; Santa et al., 2021; Santoro et al., 2024). Complete sequences of the mitochondrial DNA (mtDNA) of *E. multilocularis* have been published (Nakao et al., 2002; Zhao et al., 2022; Bohard et al., 2023), but the technical and time effort was considerable. Therefore, faster methods are needed for routine work and molecular typing. One approach to characterise the diversity and distribution of *E. multilocularis* is the use of the EmsB microsatellite locus (Bart et al., 2006). With the help of this microsatellite marker on chromosome 5 of the genomic DNA (gDNA) of *E. multilocularis*, a finer classification at the country level has been performed (Knapp et al., 2009; Knapp et al., 2010; Herzig et al., 2021). Since the EmsB represents a single marker consisting of a short piece of repetitive DNA sequence, resolution of microdiversity at the local level may be underestimated. Hence, to increase the typing resolution, which is essential for molecular epidemiology, it might be advisable to use the entire mitochondrial genome as a basis for estimation of the molecular diversity on the local but also on the global geospatial scale. This can be achieved by the Region-Specific Extraction (RSE) method (Dapprich et al., 2016), which can help to generate complete mitogenome sequences.

Here we demonstrate that the long-range DNA target capture RSE method, modified and validated for the *E. multilocularis* mitogenome, can be used in combination with an Illumina NGS platform as a tool for extraction, capturing, sequencing, and genotyping of DNA from individual *E. multilocularis* specimens at the whole mitochondrial genome level.

## 2 Materials and methods

### 2.1 Study area and parasites

All *E*. *multilocularis* adult parasite samples came from Germany and were collected by the German State Veterinary Investigation Centres of the Federal States of Brandenburg, Thuringia, and Lower Saxony or by local hunters in these Federal States (for details, see Supplementary 2, Table S2). We obtained two samples from Lower Saxony (Em_1 and Em_6), three samples from Brandenburg (Em_3, Em_5, and Em_12) and nine samples from Thuringia (Em_2, Em_4, Em_7 to Em_11, Em_13 and Em_14). The samples consisted of worms from the intestinal mucosa of Red Foxes (*Vulpes vulpes*). The sample of *E. granulosus* cyst material (Eg_1; Supplementary 2, Table S2) was obtained from Kenya (cattle) and was used in the microsatellite analysis as an outgroup control.

For biosafety reasons, carcases of infected definitive hosts and samples that could contain adult stages or eggs were stored for at least 1 week at −80°C to inactivate eggs (Jacobs et al., 1994; Eckert et al., 2001).

### 2.2 The use of parasites in the experiments

To estimate the minimum required amount of worm material for the RSE method and to look if it changes the SNP number and SNP profile, different numbers of worms were processed. Starting with a single adult parasite, in which all proglottids were removed with a scalpel, to samples with eight worms (Supplementary 2, Table S2). The proglottids were removed from the worms (Em_3 to Em_6; Supplementary 2, Table S2) to see if there was a difference in the SNP profile between samples without proglottids and samples with proglottids (with eggs; all other samples in Supplementary 2, Table S2). Furthermore, the approach was used to study, whether it is possible to use such a small amount of sample material (due to the fact that the number of worms per Red Fox can be very small). The samples came from nine Red Foxes (Red Fox 1 to Red Fox 9; Supplementary 2, Table S2).

We deliberately used several adult parasites from a single Red Fox to determine the repeatability and accuracy regarding the detected SNPs. Furthermore, the samples Em_8 and Em_9 were derived from the same DNA (technical replicate) to test the accuracy when used for RSE. A dilution series was prepared to analyse the analytical detection limit. A selected DNA sample (Em_14) was serially diluted in a logarithmic (log_2_) fashion, achieving a maximum dilution of 1:64. For enhanced readability the dilutions were consecutively labelled from undiluted (Em_14) to 1:64 (Em_20) (see Supplementary 2, Table S2). Furthermore, the undiluted sample Em_14 was also included in all subsequent analyses as a reference.

### 2.3 List of reagents, materials, software, and equipment

The reagents, materials, software, and equipment used in this study are described in Supplementary 1.

### 2.4 Collection of adult *Echinococcus multilocularis* parasites from the Red Fox intestinal mucosa

Adult parasites were collected by the Sedimentation and Counting Technique (SCT) as described (Eckert et al., 1984; Eckert et al., 2001; Maksimov et al., 2017). The *E. multilocularis* specimens were picked from the sediment of intestinal mucosa using a stereomicroscope and a 10 μL pipette with filter tips. For the experiments, either complete adult parasites were used or all proglottids were separated with a scalpel (one separate scalpel per worm to avoid cross-contamination). The anterior ends of the adult worms or the whole parasites were transferred into 10 μL 0.1X TE buffer in 1.5 mL Eppendorf tubes (one specimen per Eppendorf tube) and the samples were stored at −20°C until further use.

### 2.5 DNA extraction with phenol-chloroform method for worm material

For DNA extraction of worm material a modification of the protocol of Pacific Biosciences of California, Inc. (PACBIO, 2012) was used (Supplementary 2, Figure S10). The thawed worms were centrifuged for 5 min at 13,000 rpm (17,949 *g*). The supernatant was removed and 200 μL of digestion buffer (not older than 2 weeks, with proteinase K) were added (preparation of the buffer, see Supplementary 1). Samples were incubated overnight at 56°C in a thermomixer at 300 rpm. Subsequently, samples were centrifuged at 500 rpm (26.55 *g*) for 1 min and one volume (200 μL) of phenol-chloroform (Phenol:Chloroform:Isoamyl Alcohol 25:24:1 saturated with 10 mM Tris, pH 8.0, 1 mM EDTA) was added to the sample. After vortexing for 1 min, the samples were centrifuged for 7 min at 14,000 rpm (20,817 *g*). A volume of 180 μL of the upper phase was transferred to a second tube and 200 μL elution buffer (EB, preparation of the buffer, see Supplementary 1) was added to the lower phase in the first tube, vortexed for 1 min, and centrifuged again for 7 min at 14,000 rpm (20,817 *g*). The upper phase was also added to the second tube. The volume of the mixed upper phases was determined, and one volume of phenol-chloroform was added. The second tube was vortexed for 1 min and then centrifuged for 7 min at 14,000 rpm (20,817 *g*). After this step, the upper phase was transferred into a third tube. The volume in this tube was determined and 1/10 volume of ammonium acetate was added to reach a final concentration of 0.75 M in the tube. Then, glycogen (20 mg/mL to an end concentration of 20 μg per tube) was added to the tube and the sample was briefly vortexed at low speed. The volume was again determined, and 2.5 volumes of 100% ice-cold ethanol were added. The tube was briefly vortexed at low speed. For the precipitation of the DNA, the tube was stored at −20°C for at least 1 h. The sample was then centrifuged at 14,000 rpm (20,817 *g*) for 20 min (at 4°C). The supernatant was discarded, 300 μL of ice-cold 80% ethanol were added to the pellet, and the tube briefly vortexed three times at low speed. The tube was then again centrifuged at 14,000 rpm (20,817 *g*) for 15 min (at 4°C). This step was repeated a second time (adding 300 μL of ice-cold 80% ethanol and centrifugation). The supernatant was discarded and the pellet dried in a thermomixer at 37°C for approx. 6 min (0 rpm). The pellet (template DNA) was dissolved in 100 μL elution buffer (EB) without mixing.

### 2.6 DNA extraction of cyst material

The DNA of utilised cyst material was not extracted as part of the present study. It has been provided for EmsB microsatellite analysis as an outgroup control. Nevertheless, the DNA extraction method is described here. For the digestion and DNA extraction of cyst material, the NucleoSpin Tissue kit (MACHEREY-NAGEL GmbH & Co. KG) was used. The cyst material was weighed, and 25 mg was placed in an Eppendorf tube. A total of 180 μL T1 buffer and 25 μL proteinase K were added and the sample incubated at 56°C for 3 h at 300 rpm in the thermomixer. The digested sample was then vortexed and briefly centrifuged at 500 rpm (26.55 *g*) for a few seconds. A volume of 200 μL of B3 buffer was added, the tube vortexed, and incubated for 10 min at 70°C in the thermomixer (at 0 rpm). The sample was then further processed according to the manufacturer’s instructions (NucleoSpin Tissue kit; 01/2017, Rev. 17). The sample was used in a 1:10 dilution (diluted with nuclease-free water).

### 2.7 Capture Primer Set for Region-Specific Extraction

For the Region-Specific Extraction (RSE) a *E. multilocularis* mtDNA Capture Primer Set (CPS) was prepared. To this end, five different established primer pairs of different PCRs were used (Supplementary 2, Table S3). These are at least *Echinococcus* spp. specific and are distributed well over the genome (Supplementary 2, Figure S1). All primers (Supplementary 2, Table S3) were purchased from metabion international AG, Planegg, Germany. The primers were centrifuged for 1 min at 500 rpm (26.55 *g*) and resolved with nuclease-free water according to the manufacturer primer report to obtain a concentration of 100 μM. To illustrate where the primers bind in the genome, the Geneious Prime^®^ programme (Biomatters, Inc., Boston, United States) was utilised (see Supplementary 2, Figure S1). The *E. multilocularis* primers were analysed for primer dimer formation using the website http://www.primer-dimer.com/ (Lu et al., 2017; Johnston et al., 2019). All possible combinations were analysed pairwise (multiplex analysis, accessed 24.11.2021, see Supplementary 2, Table S4).

The calculation of the CPS concentration was performed according to Dapprich et al. (2016). A volume of 2 μL of each primer (with an initial concentration of 100 μM) was added to the mix. The following primers were used (for sequences and further information see also Supplementary 2, Table S3): EM-H15_F and EM-H17_R (Stieger et al., 2002; Trachsel et al., 2007), Cest1 and Cest2 (Trachsel et al., 2007), JB11.5 and JB12.5 (Bowles and McManus, 1993), F/CO1 and R/CO1 (Xiao et al., 2003), and atp6st for and atp6st rev (Herzig, 2019; Herzig et al., 2021). All primers together resulted in a mix of 20 μL (100 μM). For a 1:5 dilution, these 20 μL were mixed with 80 μL of nuclease-free water (20 μM).

### 2.8 TaqMan^®^ real-time qPCRs for monitoring the results

For monitoring the results of the RSE experiment but also for a testing of the extracted *E. multilocularis* DNA samples, a modification of the TaqMan^®^ real-time qPCR described by Isaksson et al. (2014) was applied as described elsewhere (Maksimov et al., 2019). The DNA samples were checked by amplification in the TaqMan^®^ real-time qPCR before they were used in the RSE method, after RSE, and after the REPLI-g Mini kit (a Multiple Displacement Amplification (MDA) method; QIAGEN, Hilden, Germany, Cat. No. / ID: 150023).

### 2.9 RSE method

**Figure 1:**
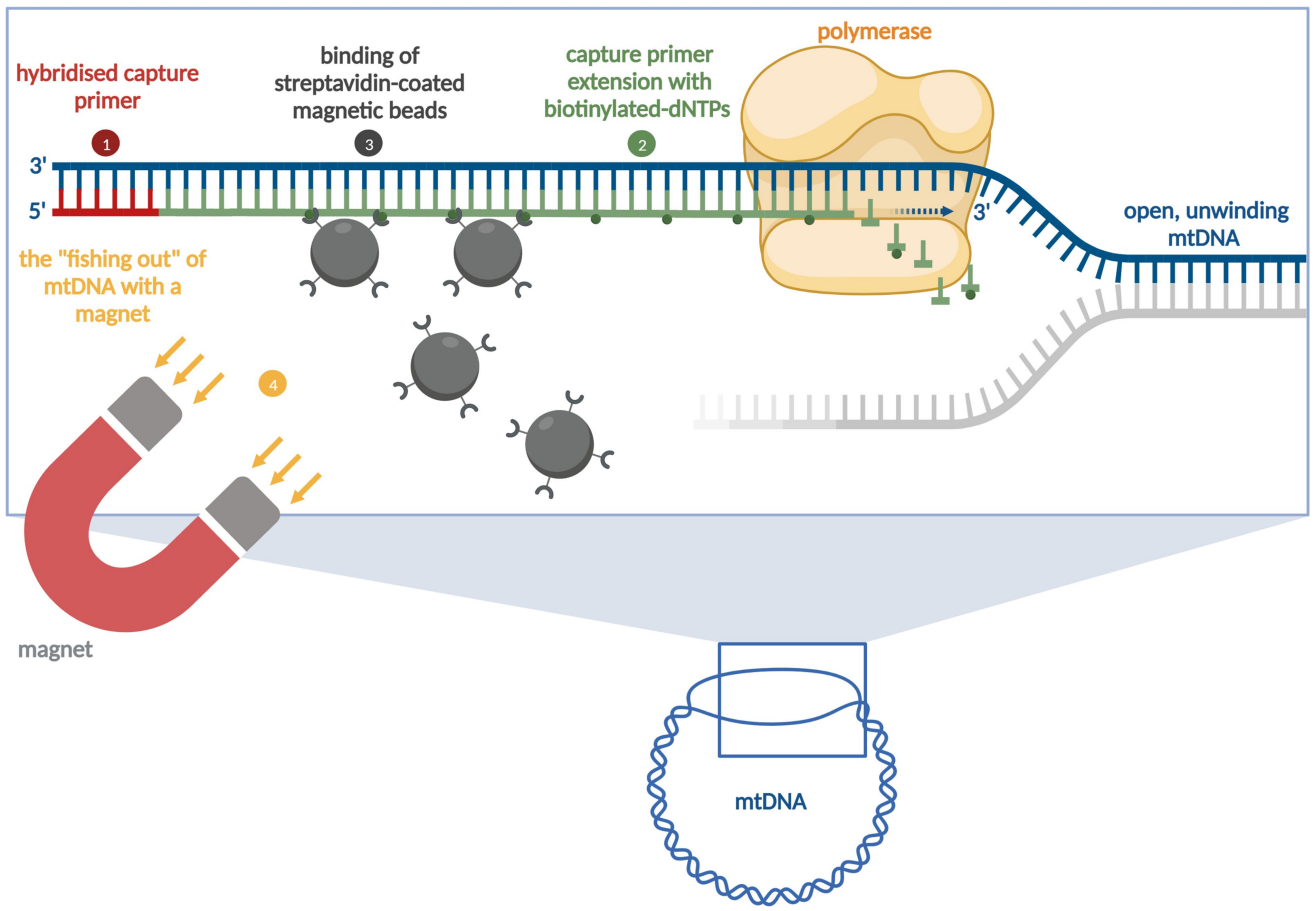
The principle of the Region-Specific Extraction (RSE) method [modified according to Dapprich et al., 2016]. The first step involves denaturing the DNA and hybridising the primers [from the Capture Primer Set (CPS)]. In the second step, the bound primers are enzymatically extended with biotinylated nucleotides. The third step involves binding the streptavidin-coated magnetic beads. In the fourth step the primer/target DNA complex is then “fished out” using a magnet, cleaned, and heat detached from the bead surface (figure not to scale). Mitochondrial DNA = mtDNA, Deoxynucleoside triphosphates = dNTPs. Created in BioRender. Rachel, F. (2025). BioRender.com.

The RSE method was developed by Dapprich et al. (2016) for the targeted sequencing of the human major histocompatibility complex (MHC) (Figure 1). The RSE method utilises capture primers and magnetic beads (Dapprich et al., 2016). With the help of the streptavidin-biotin bond, the mtDNA from *E. multilocularis* can be captured and amplified using a subsequent MDA method (Dapprich et al., 2016). For the RSE method, the phenol-chloroform-extracted parasite DNAs, the prepared CPS, the RSE kit (Generation Biotech, LLC, Princeton, United States), and the REPLI-g Mini kit (MDA method, QIAGEN, Hilden, Germany) were used. For use with *E. multilocularis* adult parasites, the method was modified by reducing (halving) the reaction volume and increasing the sample volume. The CPS concentration (5 μM) remained the same. The procedure was as follows: First, two water baths were heated up to 92°C and 60°C, respectively. Under a PCR cabinet, 22.5 μL of H-solution from RSE kit (RSE-H; room temperature), 11.25 μL of CPS, and 6.25 μL of nuclease-free water were added to a 1.5 mL Eppendorf tube. Subsequently, 5 μL of DNA were added (45 μL reaction volume). Then, DNA denaturation was carried out for 5 min at 92°C (water bath), followed by primer extension at 60°C for 15 min (water bath). Afterwards, each sample was taken individually from the water bath and 45 μL of freshly resuspended magnetic microparticles (RSE-B; room temperature) were added. The sample was carefully mixed with the beads using the pipette. This was followed by incubation for 1 h at room temperature in the thermomixer (500 rpm). The sample was then briefly centrifuged and placed in a magnetic rack for 3 min. The supernatant was withdrawn with a pipette and discarded. A volume of 120 μL wash buffer from RSE kit (RSE-W; room temperature) was added to the tube without resuspensions of the beads. The incubation time on the magnetic rack was 3 min. The supernatant was removed and then 330 μL wash buffer was added to the tube without resuspending the beads. The incubation time on the magnetic rack was again 3 min. The supernatant was discarded and 45 μL resuspension solution (RSE-R) was added to the beads and mixed carefully with the pipette. To detach the DNA from the beads, the sample was placed in a thermomixer (0 rpm) at 82°C for 15 min. The sample was then briefly centrifuged and placed in a magnetic rack for 3 min. The supernatant was transferred into a new 1.5 mL Eppendorf tube (RSE Magnetic Capture Eluate = MCE). When the RSE had been completed, the DNA was amplified. The REPLI-g Mini Kit was used for this purpose. All required buffers (DLB, D1 and N1) were prepared according to the manufacturer’s instructions. In a new 1.5 mL Eppendorf tube, 29 μL REPLI-g Mini Reaction Buffer were mixed with 1 μL REPLI-g Mini DNA Polymerase (30 μL master mix). Furthermore, 10 μL of D1 buffer and 24 μL of MCE were added to another 1.5 mL Eppendorf tube and incubated for 3 min at room temperature before 20 μL of prepared N1 buffer were added. The solution was mixed carefully. To 30 μL master mix, 20 μL of denatured DNA were added and mixed carefully with the pipette. This preparation was incubated overnight for 16 h at 30°C in a thermomixer (0 rpm). After this time, an inactivation step was carried out for 5 min at 65°C in a thermomixer (0 rpm). The sample was then briefly centrifuged. The sample was stored at 4°C. All “after REPLI-g” samples were then sent for sequencing (NGS).

### 2.10 Dilution series

A serial dilution was prepared to determine the detection limit of the RSE method. To this end, 40 μL of nuclease-free water were placed in 1.5 mL Eppendorf tubes and then 40 μL of DNA or the respective dilution added, thoroughly mixed and transferred to the next tube (from undiluted to 1:64). The DNA concentrations were determined by NanoDrop™ 2000 spectrophotometer. A control qPCR (Isaksson et al., 2014; Maksimov et al., 2019) was also performed using samples of the serial dilutions (with three technical replicates of each dilution step). The DNA samples extracted directly from the worms (“before RSE”), the DNA samples processed in the RSE protocol but not amplified by REPLI-g kit (“after RSE”), and DNA samples processed completely by RSE method (“after REPLI-g”) were amplified by mentioned qPCR to additionally control the amount of mitogenome DNA (qPCR conditions see above). With the “after REPLI-g” samples the limits in the NGS data were analysed (these were measured with the MiSeq™ device). The programmes for data analysis can be found in Supplementary 1.

### 2.11 EmsB microsatellite analysis

The EmsB primers (Supplementary 2, Table S3) were used as described by Bart et al. (2006) and the PCR protocol was adapted as described by Herzig et al. (2021). Briefly, the volume of the PCR mix was 25 μL and included 2.5 μL 10X buffer, 2 μM forward (EmsB A), 2 μM reverse primer (EmsB C), 400 μM dNTPs, 3 mM MgCl_2_, 4 U/rxn platinum Taq DNA polymerase, nuclease-free water, 7% DMSO, and 4 μL template DNA (for more information of the reagents see also Supplementary 1). The PCR cycling conditions consisted of an initial denaturation for 2 min at 94°C, 40 cycles with a denaturation step for 30 s at 94°C, annealing for 30 s at 60°C, and elongation for 30 s at 72°C. For the EmsB microsatellite analysis, the samples were further processed as follows: for every sample 10 μL Hi-Di™ Formamide was mixed with 0.3 μL ROX 500 (size standard). To this mix 1 μL PCR product was added. Immediately prior to analysis in the genetic analyser instrument (Applied Biosystems Hitachi 3500), samples were incubated at 95°C for 5 min in a thermomixer. The analysis of data were carried out according to the EmsB analysis guidelines (Knapp et al., 2017) with the computer software GeneMapper™ (Applied Biosystems™), Microsoft Excel (Microsoft Corporation), R (R Core Team, 2022), R package pvclust (Suzuki and Shimodaira, 2006), and RStudio [Posit Software, PBC formerly RStudio, PBC (RStudio Team, 2020)]. More information about the programmes for data analysis can be found in Supplementary 1.

### 2.12 Next-Generation Sequencing (NGS)

For Next-Generation Sequencing (NGS), the samples (4 DNA samples for WGS and the “after REPLI-g” samples) were sent either to the NGS unit of the Institute of Virus Diagnostics, Friedrich-Loeffler-Institut, Greifswald – Insel Riems, Germany (Illumina MiSeq™) or to Eurofins (Genomics Germany GmbH, NovaSeq™ 6,000).

### 2.13 Bioinformatic data analysis

Sequencing of *E. multilocularis* was conducted using Illumina sequencing technologies (Supplementary 1). The quality of the Illumina NGS “fastq” data was evaluated with the ‘fastQC’ program package.^1^

For variant calling, the respective reads were mapped by BWA-MEM (Burrows-Wheeler Aligner, Maximal Exact Match) (Li and Durbin, 2009) to the *E. multilocularis* reference genomes (BioProject no. PRJEB122) with the annotation Version 2015-12-WormBase downloaded from the website WormBase ParaSite^2^ (Tsai et al., 2013; Howe et al., 2016; Howe et al., 2017) and “Freebayes” software was used to call variants in the respective genomes, thus creating isolate specific VCF (Variant Call Format) files (Garrison and Marth, 2012). All VCF files were then combined with the “Bcftool merge” software to prepare the data for further analysis. In the next step the called variants were filtered by “vcftool” software applying the hard filtering parameter “-minGQ15” “--minDP 10” to validate the called genetic variants (Danecek et al., 2011; Danecek et al., 2021). Further filtering, validation, merging, comparing, simple statistics, and other manipulations of the annotated VCF files were performed with ‘SNPSift’ tool (Cingolani et al., 2012), ‘VCFTools’ (Danecek et al., 2011), and ‘bcftools’ (Li et al., 2009). Further downstream statistic and genetic analysis were done using R (R Core Team, 2022) and RStudio (RStudio Team, 2020).

### 2.14 Haplotype network

**Figure 2:**
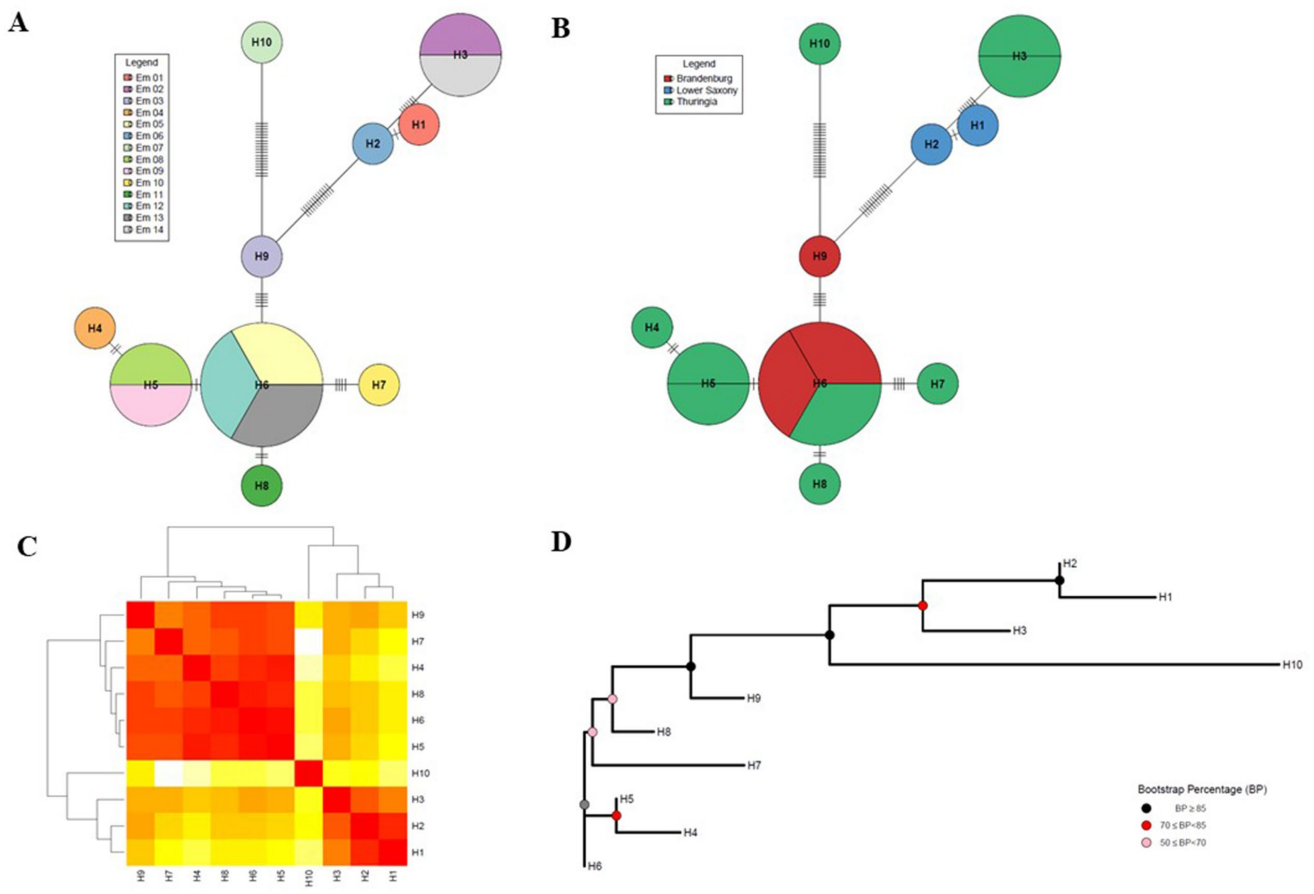
Haplotype network analysis of the *Echinococcus multilocularis* mitogenome. **(A)** Haplotype network represented by individuals. The name in the legend abbreviations stand for: Em_X = *Echinococcus multilocularis* sample with sample ID (with *X* = 1–14). **(B)** Haplotype network sorted by region. The colours in the illustration abbreviations stand for: red = Brandenburg, blue = Lower Saxony, and green = Thuringia. HX = Haplotype number (with *X* = 1–10). **(C)** Heat map based on the number of nucleotide differences between the haplotypes. Each branch of the phylogenetic tree represents the corresponding haplotype in the matrix. Colours: dark red = close relationships, white = far relationships. **(D)** Neighbour-joining (NJ) tree for mitogenome of *Echinococcus multilocularis* (Hamming distance method of nucleotide differences). Coloured internal nodes represent the bootstrap confidence level (values were specified by colouring according to confidence intervals). Bootstrap Percentage (BP) ≥85 the confidence interval is strong, 70 ≤ BP < 85 moderate, and weak for 50 ≤ BP < 70. **(A–D)** Created after (Toparslan et al., 2020).

To find out whether the tested *E. multilocularis* group harbours different haplotypes, a haplotype network analysis was applied to analyse the frequency and number of haplotypes, as well as to calculate a haplotype distance matrix [all R packages and the used R script can be found in Toparslan et al. (2020)]. Furthermore, a heat map based on the number of nucleotide differences between the haplotypes and a Neighbour-joining (NJ) tree (Hamming distance method of nucleotide differences) for the mitogenome of *E. multilocularis* was generated (Figure 2 and Supplementary 2, Figures S7, S9, Tables S11–S13). To confirm the results of the network above, a haplotype network (integer NJ network) was generated using the PopArt programme (version 1.7) from the website http://popart.otago.ac.nz (French et al., 2014; Leigh and Bryant, 2015; Supplementary 2, Figure S8, Table S10).

## 3 Results

### 3.1 Capture Primer Set and primer dimer detection

The results of the primer dimer detection, summarised in Supplementary 2, Table S4, shows that all ΔG values are above −9 kcal/mol (−6.78 kcal/mol to 2.1 kcal/mol). Such values are generally considered acceptable. The selected Capture Primer Set allowed for the extraction of the entire mitochondrial genome (~14 kb long) in the respective samples using the RSE protocol.

### 3.2 Testing of analytical sensitivity

A dilution series was used to determine the detection limit. The measurement of the DNA concentration using the NanoDrop™ 2000 spectrophotometer indicated (measured as a technical triplet) that DNA could still be detected up to a dilution of 1:8. This dilution level corresponded to a DNA concentration of approx. 0.6 ng/μL (Supplementary 2, Figure S2, Table S5). The results of the qPCR for the dilution series DNA samples “before RSE” (DNA samples before processing in the RSE protocol), “after RSE” (DNA samples obtained/captured by Capture Primer Set but not amplified by the MDA method in RSE protocol yet), and “after REPLI-g” (samples obtained/captured by Capture Primer Set and finally amplified by the MDA method to amplify the specifically captured DNA regions) showed (Supplementary 2, Figure S3) that there was no longer an increase in the amount of DNA (red curve of “after REPLI-g” samples) at dilutions higher than 1:8 (green curve of the “before RSE” samples). It should be noted that, probably for technical reasons during the RSE experiment, there was no increase in the DNA concentration for the 1:2 dilution “after the REPLI-g” (red curve) (Supplementary 2, Figure S3). All other dilution levels show a reasonable increase in ct values (a.k.a, cq values) over the course of the dilution series (Supplementary 2, Figure S3). In the Supplementary 2, Figure S3, Table S6, only two of three measurements are listed for the undiluted sample, due to a measurement error on the device. In summary, the cut-off is at a ct value of ~25 (Supplementary 2, Table S6).

**Figure 3:**
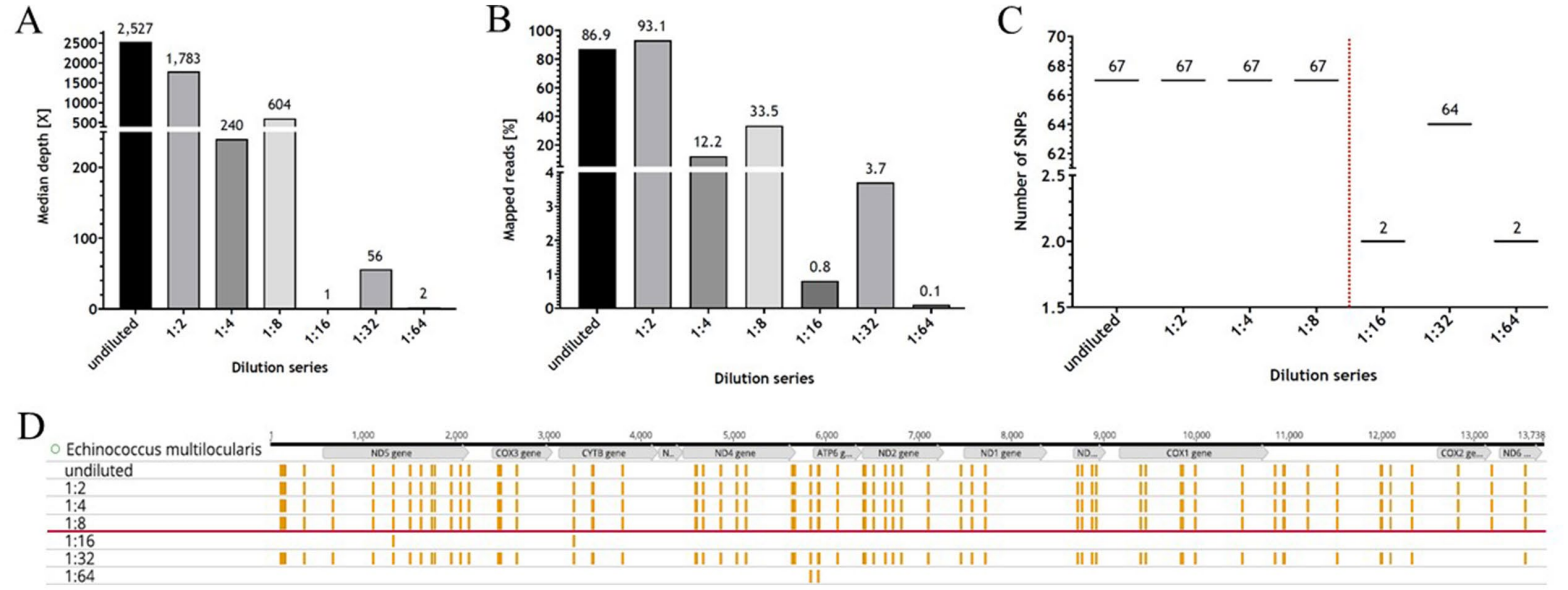
The NGS data of the dilution series. The results of the programme “multiqc” (MultiQC v1.12 – written by Phil Ewels, available on GitHub). **(A,B)** Are graphical representations of the Table 1 for median depth and mapped reads, respectively. **(C)** Depicts the number of Single Nucleotide Polymorphisms (SNPs) as a function of the dilution level (from undiluted to 1:64). The number of SNPs is stable up to a dilution of 1:8 (red dotted line). **(D)** Shows the mitochondrial genome (mtDNA) of *Echinococcus multilocularis* (Nakao et al., 2002) with the position of the genes (in grey), as well as the SNPs (presented in yellow) for the respective dilution level. The red line indicates the detection limit. The illustration was created with GraphPad Prism^®^ **(A–C)** and Geneious Prime^®^ **(D)**.

The results of the NGS analysis for the dilution series for the after REPLI-g samples (Table 1) showed that the coverage of the genome ranges from 100 to 0%, with not much difference between the 30X and 50X depth values. However, the values decreased steadily with increasing dilutions up to 1:8, after which there was a significant drop in values down to 0% coverage (Table 1). The median depth of the dilution series ranges from 1.0X (1:16 dilution level) to 2,527.0X (undiluted) (Figure 3 and Table 1). The median depth also remained stable up to the 1:8 dilution level (604.0X). The percentage of mapped reads ranged from 93.1% (1:2) to 0.1% (1:64), whereby the values of the absolute numbers in millions of mapped reads were similar (Table 1). In conclusion, the detection limit for the RSE method was found in a 1:8 dilution and presenting a ct value of ~25.

**Table 1:** The NGS data of the dilution series.

Dilution	≥30X	≥50X	Med. depth [X]	% Aligned	M Aligned	M Total reads
Undiluted	100.0%	100.0%	2527.0	86.9%	0.2	0.2
1:2	100.0%	100.0%	1783.0	93.1%	0.2	0.2
1:4	99.0%	95.7%	240.0	12.2%	0.0	0.2
1:8	98.3%	91.7%	604.0	33.5%	0.1	0.2
1:16	0.0%	0.0%	1.0	0.8%	0.0	0.0
1:32	71.1%	55.1%	56.0	3.7%	0.0	0.2
1:64	0.0%	0.0%	2.0	0.1%	0.0	0.2

The results of the programme “multiqc” (MultiQC v1.12 – written by Phil Ewels, available on GitHub). ≥ 30X = proportion of genome with at least 30-fold coverage, ≥ 50X = proportion of genome with at least 50-fold coverage, Med. depth = median depth, % Aligned = percentage of mapped reads, M Aligned = number of mapped reads (in millions), and M Total reads = number of reads (in millions).

### 3.3 qPCR, RSE, and NGS results of the field samples

**Figure 4:**
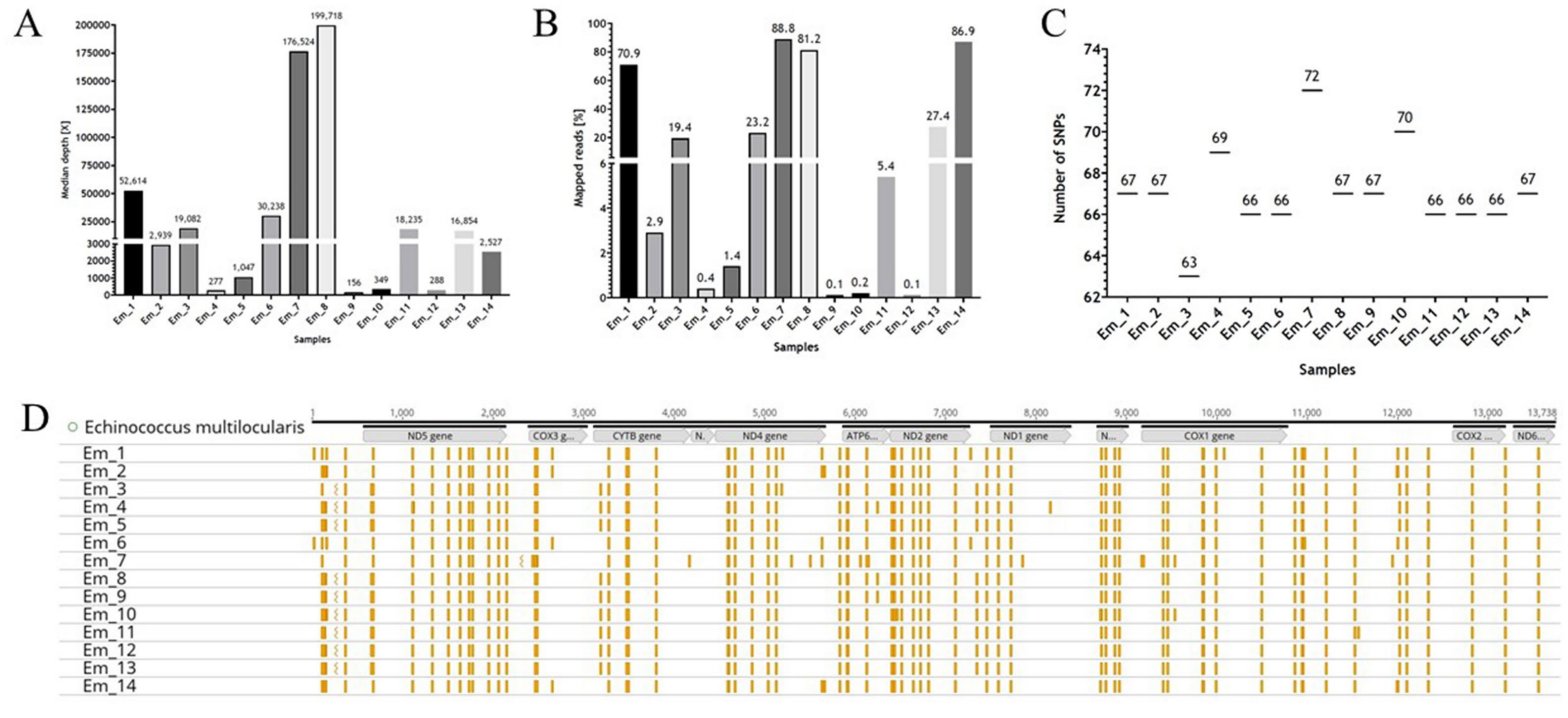
The NGS data of the samples. **(A,B)** Are graphical representations of the Table 3 for median depth and mapped reads, respectively. **(C)** Depicts the number of Single Nucleotide Polymorphisms (SNPs) as a function of sample. **(D)** Shows the mitochondrial genome (mtDNA) of *Echinococcus multilocularis* (Nakao et al., 2002) with the position of the genes (in grey), as well as the SNPs (presented in yellow) for the samples. The illustration was created with GraphPad Prism^®^ **(A–C)** and Geneious Prime^®^ **(D)**.

For the samples used in this study, qPCR analyses were also carried out on the samples “before RSE” (DNA samples extracted directly from the worms), “after RSE” (DNA samples processed according to the RSE protocol but not amplified by REPLI-g kit), and “after REPLI-g” (DNA samples processed completely by RSE method) to estimate the success of the RSE method relative to the amount of recovered DNA in different steps of the RSE method. All “before RSE” samples met the required ct value of <25.0 (Table 2). The values of the “before RSE” samples varied from ct 17.7 (sample Em_11 with 8 worms) to 24.8 (Em_6 with 1 worm, from which the proglottids were removed) (Table 2 and see also for more information of the samples Supplementary 2, Table S2). The ct values of all samples first increased after the RSE method (Table 2). The ct values varied from 22.5 (Em_11 with 8 worms) to 29.3 (Em_4 with 1 worm, from which the proglottids were removed) (Table 2). After the unspecific amplification of the captured *E. multilocularis* mtDNA by the REPLI-g kit (MDA method), the ct values for most samples decreased significantly (Table 2 and Supplementary 2, Figure S4), so that ct values of 24.7 (sample Em_9 with 1 worm) to 6.4 (sample Em_6 with 1 worm, from which the proglottids were removed) were obtained. These DNA quantities were sufficient for the NGS measurement. The overview of the NGS data (Table 3; measured with the NovaSeq™ instrument) shows that the coverage was 100% for the majority of the samples, both for a depth of 30X and 50X. For samples that did not reach 100% coverage, the values ranged from 98.8 to 99.6% (for 30X) and from 83.2 to 98.1% (for 50X). All samples reached at least a median depth of 156X (up to a median depth of 199,718.0X; Figure 4A and Table 1). The number of mapped reads (Figure 4B) of the samples ranged from 0.1% (Em_9 and Em_12) to 88.8% (Em_7). Figure 4C shows also that the samples contained different numbers of SNPs. The largest number (72 SNPs, Em_7) was found in the sample with 8 worms and the lowest (63 SNPs) in a sample Em_3 (Figure 4C; for more information of the sample see Supplementary 2, Table S2). An overview of all SNPs (Figure 4D and Table 4) showed that there were monomorphic SNPs relative to the reference genome in all samples. At the same time, there were also sample-specific or polymorphic SNPs.

**Table 2:** DNA concentration results of the samples.

Sample ID	“Before RSE” sample [ct value]	“After RSE” sample [ct value]	“After REPLI-g” sample [ct value]
Em_1	22.9	26.5	11.1
Em_2	22.6	28.0	7.2
Em_3	21.6	26.5	6.9
Em_4	24.1	29.3	11.1
Em_5	22.6	27.2	10.7
Em_6	24.8	29.2	6.4
Em_7	21.2	26.5	7.9
Em_8	20.4	26.2	6.9
Em_9	22.0	27.1	24.7
Em_10	20.5	25.5	22.8
Em_11	17.7	22.5	17.5
Em_12	20.5	25.6	23.4
Em_13	21.7	28.9	9.1
Em_14	21.8	24.9	7.7
All samples	<25.0		

The measured ct values for the “before RSE,” “after RSE,” and “after REPLI-g” samples are shown. All “before RSE” samples had a ct value <25.

**Table 3:** The NGS data of the samples.

Sample ID	≥30X	≥50X	Med. depth [X]	% Aligned	M Aligned	M Total reads
Em_1	100.0%	100.0%	52614.0	70.9%	8.2	11.6
Em_2	99.6%	96.9%	2939.0	2.9%	0.3	11.4
Em_3	100.0%	100.0%	19082.0	19.4%	2.3	11.9
Em_4	99.6%	98.1%	277.0	0.4%	0.0	8.6
Em_5	100.0%	100.0%	1047.0	1.4%	0.2	11.6
Em_6	100.0%	100.0%	30238.0	23.2%	3.2	13.8
Em_7	100.0%	100.0%	176524.0	88.8%	23.9	26.9
Em_8	100.0%	100.0%	199718.0	81.2%	21.3	26.2
Em_9	98.8%	83.2%	156.0	0.1%	0.0	28.1
Em_10	99.5%	95.6%	349.0	0.2%	0.0	21.3
Em_11	100.0%	100.0%	18235.0	5.4%	1.7	32.5
Em_12	99.4%	94.8%	288.0	0.1%	0.0	26.4
Em_13	100.0%	100.0%	16854.0	27.4%	2.4	8.9
Em_14	100.0%	100.0%	2527.0	86.9%	0.2	0.2

The results of the programme “multiqc” (MultiQC v1.12 – written by Phil Ewels, available on GitHub). ≥ 30X = proportion of genome with at least 30-fold coverage, ≥ 50X = proportion of genome with at least 50-fold coverage, Med. depth = median depth, % Aligned = percentage of mapped reads, M Aligned = number of mapped reads (in millions), and M Total reads = number of reads (in millions).

**Table 4:** Overview of the results for the samples.

Gene			Lower Saxony		Brandenburg			Thuringia										
	Position [bp]	SNP_ID	Em_1	Em_6	Em_3	Em_5	Em_12	Em_4	Em_2	Em_7	Em_8	Em_9	Em_10	Em_11	Em_13	Em_14	Change	Polymorphism Type
	16	Em_mtSNP_16	G	G													A - > G	Substitution
	112	Em_mtSNP_112	A	A	A	A	A	A	A	A	A	A	A	A	A	A	C - > A	Substitution
	132	Em_mtSNP_132				T	T	T			T	T	T	T	T		A - > T	Substitution
	134	Em_mtSNP_134				T	T	T			T	T	T	T	T		A - > T	Substitution
	141	Em_mtSNP_141				A	A	A	A		A	A	A	A	A	A	G - > A	Substitution
	148	Em_mtSNP_148				C	C	C	C		C	C	C		C	C	T - > C	Substitution
	155	Em_mtSNP_155	T	T					T				T			T	A - > T	Substitution
	157	Em_mtSNP_157	T	T					T				T			T	A - > T	Substitution
	363	Em_mtSNP_363	T	T	T	T	T	T	T	T	T	T	T	T	T	T	C - > T	Substitution
*nd*5	658	Em_mtSNP_658			A	A	A	A			A	A	A	A	A		G - > A	Substitution
	671	Em_mtSNP_671	A	A	A	A	A	A	A	A	A	A	A	A	A	A	C - > A	Substitution
	1105	Em_mtSNP_1105	T	T	T	T	T	T	T	T	T	T	T	T	T	T	C - > T	Substitution
	1120	Em_mtSNP_1120						A									G - > A	Substitution
	1325	Em_mtSNP_1325	T	T	T	T	T	T	T	T	T	T	T	T	T	T	C - > T	Substitution
	1502	Em_mtSNP_1502	T	T	T	T	T	T	T	T	T	T	T	T	T	T	C - > T	Substitution
	1631	Em_mtSNP_1631	A	A	A	A	A	A	A	A	A	A	A	A	A	A	G - > A	Substitution
	1738	Em_mtSNP_1738	G	G	G	G	G	G	G	G	G	G	G	G	G	G	A - > G	Substitution
	1774	Em_mtSNP_1774	C	C	C	C	C	C	C	C	C	C	C	C	C	C	T - > C	Substitution
	1947	Em_mtSNP_1947	G	G	G	G	G	G	G	G	G	G	G	G	G	G	A - > G	Substitution
	2055	Em_mtSNP_2055	A	A	A	A	A	A	A	A	A	A	A	A	A	A	G - > A	Substitution
	2144	Em_mtSNP_2144	G	G	G	G	G	G	G	G	G	G	G	G	G	G	A - > G	Substitution
*cox*3	2440	Em_mtSNP_2440								A							G - > A	Substitution
	2466	Em_mtSNP_2466	T	T	T	T	T	T	T	T	T	T	T	T	T	T	C - > T	Substitution
	2471	Em_mtSNP_2471								G							T - > G	Substitution
	2484	Em_mtSNP_2484	G	G	G	G	G	G	G	G	G	G	G	G	G	G	A - > G	Substitution
	2658	Em_mtSNP_2658	T	T					T							T	C - > T	Substitution
*cyt*b	3191	Em_mtSNP_3191			G	G	G	G			G	G	G	G	G		A - > G	Substitution
	3275	Em_mtSNP_3275	C	C	C	C	C	C	C	C	C	C	C	C	C	C	T - > C	Substitution
	3479	Em_mtSNP_3479	C	C	C	C	C	C	C	C	C	C	C	C	C	C	T - > C	Substitution
	3490	Em_mtSNP_3490	A	A	A	A	A	A	A	A	A	A	A	A	A	A	G - > A	Substitution
	3798	Em_mtSNP_3798	C	C	C	C	C	C	C	C	C	C	C	C	C	C	T - > C	Substitution
	4172	Em_mtSNP_4172								G							A - > G	Substitution
*nd*4	4589	Em_mtSNP_4589	G	G	G	G	G	G	G	G	G	G	G	G	G	G	A - > G	Substitution
	4601	Em_mtSNP_4601	A	A	A	A	A	A	A	A	A	A	A	A	A	A	G - > A	Substitution
	4671	Em_mtSNP_4671	A	A	A	A	A	A	A	A	A	A	A	A	A	A	G - > A	Substitution
	4859	Em_mtSNP_4859	C	C	C	C	C	C	C	C	C	C	C	C	C	C	T - > C	Substitution
	5036	Em_mtSNP_5036	C	C	C	C	C	C	C	C	C	C	C	C	C	C	T - > C	Substitution
	5132	Em_mtSNP_5132	T	T	T	T	T	T	T	T	T	T	T	T	T	T	C - > T	Substitution
	5183	Em_mtSNP_5183			A												G - > A	Substitution
	5200	Em_mtSNP_5200	T														C - > T	Substitution
	5294	Em_mtSNP_5294								G							A - > G	Substitution
	5507	Em_mtSNP_5507								A							G - > A	Substitution
	5637	Em_mtSNP_5637	C	C					C	C						C	T - > C	Substitution
	5658	Em_mtSNP_5658							G							G	A - > G	Substitution
	5835	Em_mtSNP_5835	C	C	C	C	C	C	C	C	C	C	C	C	C	C	T - > C	Substitution
*atp*6	5911	Em_mtSNP_5911	T	T	T	T	T	T	T	T	T	T	T	T	T	T	C - > T	Substitution
	5924	Em_mtSNP_5924	G	G	G	G	G	G	G	G	G	G	G	G	G	G	T - > G	Substitution
	6055	Em_mtSNP_6055								C							T - > C	Substitution
	6125	Em_mtSNP_6125	A	A	A	A	A	A	A	A	A	A	A	A	A	A	T - > A	Substitution
	6150	Em_mtSNP_6150								A							G - > A	Substitution
	6247	Em_mtSNP_6247						T			T	T					C - > T	Substitution
*nd*2	6404	Em_mtSNP_6404	G	G	G	G	G	G	G	G	G	G	G	G	G	G	A - > G	Substitution
	6430	Em_mtSNP_6430	T	T	T	T	T	T	T	T	T	T	T	T	T	T	G - > T	Substitution
	6460	Em_mtSNP_6460											A				G - > A	Substitution
	6511	Em_mtSNP_6511	C	C	C	C	C	C	C	C	C	C	C	C	C	C	T - > C	Substitution
	6640	Em_mtSNP_6640	T	T	T	T	T	T	T	T	T	T	T	T	T	T	A - > T	Substitution
	6726	Em_mtSNP_6726	G	G	G	G	G	G	G	G	G	G	G	G	G	G	T - > G	Substitution
	6811	Em_mtSNP_6811	C	C	C	C	C	C	C	C	C	C	C	C	C	C	T - > C	Substitution
	7105	Em_mtSNP_7105	G	G	G	G	G	G	G	G	G	G	G	G	G	G	A - > G	Substitution
	7277	Em_mtSNP_7277	A	A													T - > A	Substitution
	7349	Em_mtSNP_7349			C	C	C	C			C	C	C	C	C		T - > C	Substitution
	7451	Em_mtSNP_7451	A	A	A	A	A	A	A	A	A	A	A	A	A	A	G - > A	Substitution
*nd*1	7578	Em_mtSNP_7578	A	A	A	A	A	A	A	A	A	A	A	A	A	A	G - > A	Substitution
	7721	Em_mtSNP_7721	T	T	T	T	T	T	T	T	T	T	T	T	T	T	C - > T	Substitution
	7725	Em_mtSNP_7725								G							T - > G	Substitution
	7848	Em_mtSNP_7848								C							T - > C	Substitution
	8156	Em_mtSNP_8156						A									G - > A	Substitution
*nd*3	8717	Em_mtSNP_8717	C	C	C	C	C	C	C	C	C	C	C	C	C	C	T - > C	Substitution
	8766	Em_mtSNP_8766	C	C	C	C	C	C	C	C	C	C	C	C	C	C	T - > C	Substitution
	8868	Em_mtSNP_8868	C	C	C	C	C	C	C	C	C	C	C	C	C	C	T - > C	Substitution
	8916	Em_mtSNP_8916	A	A	A	A	A	A	A	A	A	A	A	A	A	A	G - > A	Substitution
	9163	Em_mtSNP_9163								G							A - > G	Substitution
*cox*1	9183	Em_mtSNP_9183								T							G - > T	Substitution
	9401	Em_mtSNP_9401	C	C	C	C	C	C	C	C	C	C	C	C	C	C	T - > C	Substitution
	9453	Em_mtSNP_9453	C	C	C	C	C	C	C	C	C	C	C	C	C	C	T - > C	Substitution
	9528	Em_mtSNP_9528											T				G - > T	Substitution
	9532	Em_mtSNP_9532								T							C - > T	Substitution
	9839	Em_mtSNP_9839	G	G	G	G	G	G	G	G	G	G	G	G	G	G	T - > G	Substitution
	9852	Em_mtSNP_9852	T	T	T	T	T	T	T	T	T	T	T	T	T	T	C - > T	Substitution
	9986	Em_mtSNP_9986	A	A	A	A	A	A	A	A	A	A	A	A	A	A	G - > A	Substitution
	10493	Em_mtSNP_10493	A	A	A	A	A	A	A	A	A	A	A	A	A	A	G - > A	Substitution
	10856	Em_mtSNP_10856	A	A	A	A	A	A	A	A	A	A	A	A	A	A	G - > A	Substitution
	10936	Em_mtSNP_10936	G	G	G	G	G	G	G	G	G	G	G	G	G	G	A - > G	Substitution
	10944	Em_mtSNP_10944	C	C	C	C	C	C	C	C	C	C	C	C	C	C	T - > C	Substitution
	10949	Em_mtSNP_10949	A	A	A	A	A	A	A	A	A	A	A	A	A	A	G - > A	Substitution
	10967	Em_mtSNP_10967	A	A													G - > A	Substitution
	11202	Em_mtSNP_11202	A	A	A	A	A	A	A	A	A	A	A	A	A	A	G - > A	Substitution
	11561	Em_mtSNP_11561												T			C - > T	Substitution
	11939	Em_mtSNP_11939								G							A - > G	Substitution
	11992	Em_mtSNP_11992							C							C	T - > C	Substitution
	11998	Em_mtSNP_11998	G	G					G							G	A - > G	Substitution
	12018	Em_mtSNP_12018			T	T	T	T			T	T	T	T	T		C - > T	Substitution
	12093	Em_mtSNP_12093	A	A	A	A	A	A	A	A	A	A	A	A	A	A	G - > A	Substitution
	12329	Em_mtSNP_12329	G	G	G	G	G	G	G	G	G	G	G	G	G	G	A - > G	Substitution
*cox*2	12823	Em_mtSNP_12823	G	G	G	G	G	G	G	G	G	G	G	G	G	G	A - > G	Substitution
	13188	Em_mtSNP_13188	G	G	G	G	G	G	G	G	G	G	G	G	G	G	A - > G	Substitution
*nd*6	13549	Em_mtSNP_13549	C	C	C	C	C	C	C	C	C	C	C	C	C	C	T - > C	Substitution
Number of SNPs			67	66	63	66	66	69	67	72	67	67	70	66	66	67		

All SNPs of all samples in the study are depicted relative to the reference genome of *Echinococcus multilocularis* (Nakao et al., 2002). Their positions in the mitogenome in base pair [bp], the SNP ID, the change at the position, as well as the number of SNPs are also shown at the bottom of the table. All SNPs that occurred in all samples are highlighted in grey.

**Figure 5:**
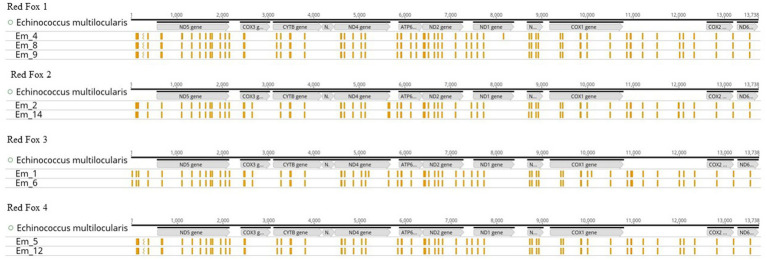
Single Nucleotide Polymorphisms (SNPs) of samples sorted by host animal (Red Fox 1–4). All graphs show the mitochondrial genome (mtDNA) of *Echinococcus multilocularis* (Nakao et al., 2002) with the position of the genes (in grey), as well as the SNPs (presented in yellow) for the samples. The illustration was created with Geneious Prime^®^.

To test the reproducibility of the results, several samples were used from a total of four Red Foxes (Figure 5), with two samples representing a technical replicate (Supplementary 2, Table S9). There was a maximum of two differences in the SNPs in each of two samples. The differences occurred in samples Em_1 and Em_4. All other SNPs of the Red Fox samples were identical.

**Figure 6:**
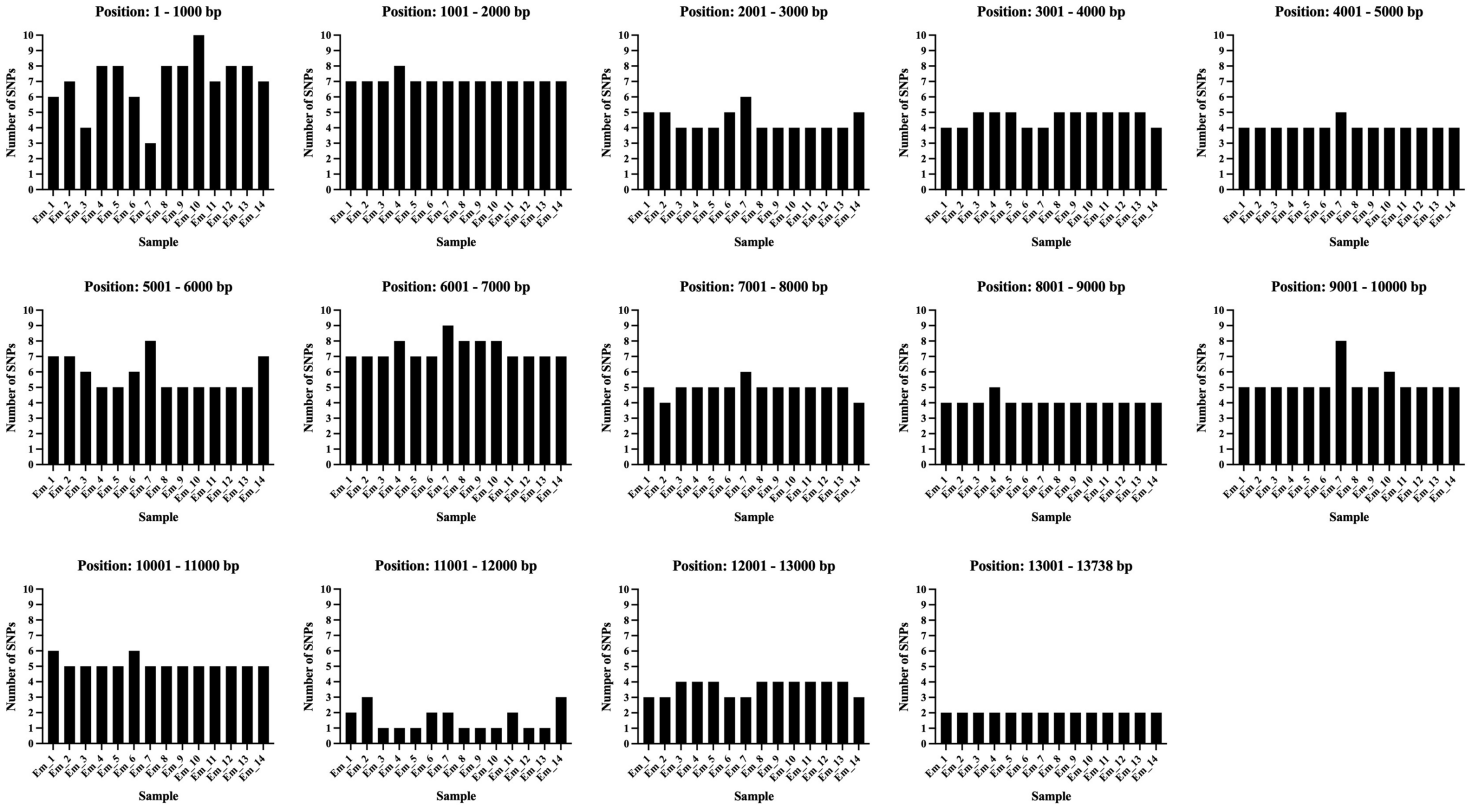
The diagram depicted the SNP density and its distribution in the mitogenome. The figure illustrates the mitogenome divided into 1,000 base sections. All samples are shown for each section. On the *y*-axis the number of SNPs are pointed. All SNPs of all samples in the study are depicted relative to the reference genome of *Echinococcus multilocularis* (Nakao et al., 2002). The illustration was created with GraphPad Prism^®^.

The observed density of SNPs (relative to the reference genome) was not evenly distributed across the mitogenome (Figure 6). To assess the differences in density, the mitogenome was divided into 1,000 base sections. The analysis showed that the variability between the samples was higher, and the number of SNPs was larger in the first 1,000 bases compared to the remaining sections (Figure 6). A similarly large number of SNPs can also be found between bases 6,000 and 7,000. A more homogeneous region is found between 11,000 and 12,000 (Figure 6).

To investigate if the RSE method provides results comparable to those of the WGS, full genome analyses were carried out on four samples (from Brandenburg and Thuringia) (Table 5 and Supplementary 2, Table S14). The Table 5 always shows a comparison of the results of the RSE method and the WGS of one sample. The data demonstrate that the SNPs of three out of the four samples were identical. Overall, only one SNP differed (yellow marking in the Table 5).

**Table 5:** Overview of the comparison of SNPs of four samples (RSE and WGS).

Gene			Thuringia						Brandenburg			
	Position [bp]	SNP_ID	Em_9_RSE	Em_9_WGS	Em_10_RSE	Em_10_WGS	Em_11_RSE	Em_11_WGS	Em_12_RSE	Em_12_WGS	Change	Polymorphism Type
	112	Em_mtSNP_112	A	A	A	A	A	A	A	A	C - > A	Substitution
	132	Em_mtSNP_132	T	T	T	T	T	T	T	T	A - > T	Substitution
	134	Em_mtSNP_134	T	T	T	T	T	T	T	T	A - > T	Substitution
	141	Em_mtSNP_141	A	A	A	A	A	A	A	A	G - > A	Substitution
	148	Em_mtSNP_148	C	C	C	C		C	C	C	T - > C	Substitution
	155	Em_mtSNP_155			T	T					A - > T	Substitution
	157	Em_mtSNP_157			T	T					A - > T	Substitution
	363	Em_mtSNP_363	T	T	T	T	T	T	T	T	C - > T	Substitution
*nd*5	658	Em_mtSNP_658	A	A	A	A	A	A	A	A	G - > A	Substitution
	671	Em_mtSNP_671	A	A	A	A	A	A	A	A	C - > A	Substitution
	1105	Em_mtSNP_1105	T	T	T	T	T	T	T	T	C - > T	Substitution
	1325	Em_mtSNP_1325	T	T	T	T	T	T	T	T	C - > T	Substitution
	1502	Em_mtSNP_1502	T	T	T	T	T	T	T	T	C - > T	Substitution
	1631	Em_mtSNP_1631	A	A	A	A	A	A	A	A	G - > A	Substitution
	1738	Em_mtSNP_1738	G	G	G	G	G	G	G	G	A - > G	Substitution
	1774	Em_mtSNP_1774	C	C	C	C	C	C	C	C	T - > C	Substitution
	1947	Em_mtSNP_1947	G	G	G	G	G	G	G	G	A - > G	Substitution
	2055	Em_mtSNP_2055	A	A	A	A	A	A	A	A	G - > A	Substitution
	2144	Em_mtSNP_2144	G	G	G	G	G	G	G	G	A - > G	Substitution
*cox*3	2466	Em_mtSNP_2466	T	T	T	T	T	T	T	T	C - > T	Substitution
	2484	Em_mtSNP_2484	G	G	G	G	G	G	G	G	A - > G	Substitution
*cyt*b	3191	Em_mtSNP_3191	G	G	G	G	G	G	G	G	A - > G	Substitution
	3275	Em_mtSNP_3275	C	C	C	C	C	C	C	C	T - > C	Substitution
	3479	Em_mtSNP_3479	C	C	C	C	C	C	C	C	T - > C	Substitution
	3490	Em_mtSNP_3490	A	A	A	A	A	A	A	A	G - > A	Substitution
	3798	Em_mtSNP_3798	C	C	C	C	C	C	C	C	T - > C	Substitution
*nd*4	4589	Em_mtSNP_4589	G	G	G	G	G	G	G	G	A - > G	Substitution
	4601	Em_mtSNP_4601	A	A	A	A	A	A	A	A	G - > A	Substitution
	4671	Em_mtSNP_4671	A	A	A	A	A	A	A	A	G - > A	Substitution
	4859	Em_mtSNP_4859	C	C	C	C	C	C	C	C	T - > C	Substitution
	5036	Em_mtSNP_5036	C	C	C	C	C	C	C	C	T - > C	Substitution
	5132	Em_mtSNP_5132	T	T	T	T	T	T	T	T	C - > T	Substitution
	5835	Em_mtSNP_5835	C	C	C	C	C	C	C	C	T - > C	Substitution
*atp*6	5911	Em_mtSNP_5911	T	T	T	T	T	T	T	T	C - > T	Substitution
	5924	Em_mtSNP_5924	G	G	G	G	G	G	G	G	T - > G	Substitution
	6125	Em_mtSNP_6125	A	A	A	A	A	A	A	A	T - > A	Substitution
	6247	Em_mtSNP_6247	T	T							C - > T	Substitution
*nd*2	6404	Em_mtSNP_6404	G	G	G	G	G	G	G	G	A - > G	Substitution
	6430	Em_mtSNP_6430	T	T	T	T	T	T	T	T	G - > T	Substitution
	6460	Em_mtSNP_6460			A	A					G - > A	Substitution
	6511	Em_mtSNP_6511	C	C	C	C	C	C	C	C	T - > C	Substitution
	6640	Em_mtSNP_6640	T	T	T	T	T	T	T	T	A - > T	Substitution
	6726	Em_mtSNP_6726	G	G	G	G	G	G	G	G	T - > G	Substitution
	6811	Em_mtSNP_6811	C	C	C	C	C	C	C	C	T - > C	Substitution
	7105	Em_mtSNP_7105	G	G	G	G	G	G	G	G	A - > G	Substitution
	7349	Em_mtSNP_7349	C	C	C	C	C	C	C	C	T - > C	Substitution
	7451	Em_mtSNP_7451	A	A	A	A	A	A	A	A	G - > A	Substitution
*nd*1	7578	Em_mtSNP_7578	A	A	A	A	A	A	A	A	G - > A	Substitution
	7721	Em_mtSNP_7721	T	T	T	T	T	T	T	T	C - > T	Substitution
*nd*3	8717	Em_mtSNP_8717	C	C	C	C	C	C	C	C	T - > C	Substitution
	8766	Em_mtSNP_8766	C	C	C	C	C	C	C	C	T - > C	Substitution
	8868	Em_mtSNP_8868	C	C	C	C	C	C	C	C	T - > C	Substitution
	8916	Em_mtSNP_8916	A	A	A	A	A	A	A	A	G - > A	Substitution
*cox*1	9401	Em_mtSNP_9401	C	C	C	C	C	C	C	C	T - > C	Substitution
	9453	Em_mtSNP_9453	C	C	C	C	C	C	C	C	T - > C	Substitution
	9528	Em_mtSNP_9528			T	T					G - > T	Substitution
	9839	Em_mtSNP_9839	G	G	G	G	G	G	G	G	T - > G	Substitution
	9852	Em_mtSNP_9852	T	T	T	T	T	T	T	T	C - > T	Substitution
	9986	Em_mtSNP_9986	A	A	A	A	A	A	A	A	G - > A	Substitution
	10493	Em_mtSNP_10493	A	A	A	A	A	A	A	A	G - > A	Substitution
	10856	Em_mtSNP_10856	A	A	A	A	A	A	A	A	G - > A	Substitution
	10936	Em_mtSNP_10936	G	G	G	G	G	G	G	G	A - > G	Substitution
	10944	Em_mtSNP_10944	C	C	C	C	C	C	C	C	T - > C	Substitution
	10949	Em_mtSNP_10949	A	A	A	A	A	A	A	A	G - > A	Substitution
	11202	Em_mtSNP_11202	A	A	A	A	A	A	A	A	G - > A	Substitution
	11561	Em_mtSNP_11561					T	T			C - > T	Substitution
	12018	Em_mtSNP_12018	T	T	T	T	T	T	T	T	C - > T	Substitution
	12093	Em_mtSNP_12093	A	A	A	A	A	A	A	A	G - > A	Substitution
	12329	Em_mtSNP_12329	G	G	G	G	G	G	G	G	A - > G	Substitution
*cox*2	12823	Em_mtSNP_12823	G	G	G	G	G	G	G	G	A - > G	Substitution
	13188	Em_mtSNP_13188	G	G	G	G	G	G	G	G	A - > G	Substitution
*nd*6	13549	Em_mtSNP_13549	C	C	C	C	C	C	C	C	T - > C	Substitution
			67	67	70	70	66	67	66	66		
	Identical		100%		100%		≈ 98.51%		100%			

All SNPs of all samples are depicted relative to the reference genome of *Echinococcus multilocularis* (Nakao et al., 2002). There was only one difference between RSE and WGS in a single sample (SNP marked in red for sample Em_11). Their positions in the mitogenome [bp], the SNP ID, the change at the position, as well as the number of SNPs and the match (in percent) are also shown at the bottom of the table. RSE, Region-Specific Extraction method; WGS, Mitogenome from Whole-Genome Sequencing; bp, base pair; SNP, Single Nucleotide Polymorphism.

### 3.4 Haplotype network

In order to determine the variability (measured as SNPs) in the mitochondrial DNA using the RSE method, a haplotype network was created using an R-script according to Toparslan et al. (2020) (Figure 2 and Supplementary 2, Figures S7, S9, Tables S11–S13). The results show that worms from the same Red Fox or from the same Federal State differed only by very few SNPs or could even attributed to the same haplotype. To confirm the results, the formation of the haplotype network was repeated with a second programme, which corroborated this result (Supplementary 2, Figure S8, Table S10). Figure 2C shows a heatmap, in which three clusters can be distinguished, which reflect a close relationship (dark red) between the haplotypes. Figure 2D shows the corresponding confidence intervals, which represent the phylogenetic relationship (Neighbour-joining (NJ) tree) between the haplotypes when analysed using the bootstrap method (Hamming distance method of nucleotide differences). The haplotype H10 forms a separate cluster, H1 to H3 belong to a second cluster and all others a third one. Haplotype H10 is a single sample (Em_7) from Red Fox 6 from Thuringia. The four samples from the second cluster came from two foxes (Red Fox 2 and Red Fox 3) from Thuringia and Lower Saxony, respectively. These are samples Em_2 (1 worm) and Em_14 (1 worm) from Thuringia and Em_1 (1 worm) and Em_6 (1 worm from which the proglottids were removed) for Lower Saxony. In the third cluster, the samples came from Thuringia and Brandenburg, whereby the three samples from Brandenburg belong to two closely related haplotypes (H9 and H6). Here, samples Em_5 and Em_12 come from Red Fox 4 and show no differences in the number of SNPs and thus form two of the three samples of haplotype H6.

All results of the haplotype analysis demonstrate that 10 haplotypes could be found in three clusters with large confidence intervals with a few samples analysed using the RSE method. Overall, the results reflect the origin of the samples and thus support the accuracy of the RSE method. It seems possible to find differences in microdiversity at the regional level (i.e., German Federal States in this case).

### 3.5 EmsB microsatellite analysis

The EmsB data yielded a homogeneous picture of the samples. A clear distinction between *E. granulosus* (as outgroup sample) and *E. multilocularis* was evident (Supplementary 2, Figures S5, S6). The peaks in the electropherogram for *E. multilocularis* ranged from 209 to 241 bp and for *E. granulosus* ranged from 260 to 284 bp (Maillard et al., 2009). The *E. multilocularis* samples belong to the European clade and may be divided into two profiles (G and D) taking the genetic distance threshold into consideration (Knapp et al., 2007).

## 4 Discussion

While adult *Echinococcus* spp. could in the past only be differentiated morphologically, it is now possible to attribute specimens of the species *E. multilocularis* roughly to different clades that cover large areas. This is possible by using molecular biological methods, which are in most cases based on a few genes or markers (Nakao et al., 2009; Ito et al., 2013; Konyaev et al., 2013; Lymbery, 2017; Šnábel et al., 2020; Santa et al., 2021). Nakao et al. (2009) were able to distinguish four clades with 18 haplotypes (E1-E5 for Europe, N1 and N2 for North America, A1 to A10 for Asia, and O1 for an undefined region) based on the analysis of one quarter of the entire mitochondrial genome. Similar results were obtained by others (Ito et al., 2013; Konyaev et al., 2013; Lymbery, 2017). Jastrzembski (2017) noted that most of these results are not based on a uniform approach (different numbers of isolates and of mitochondrial genes, etc.). The use of the complete mitogenome would thus be helpful to obtain clearer results.

Complete sequences of the mitogenome of *E. multilocularis* have been published (Nakao et al., 2002; Zhao et al., 2022; Bohard et al., 2023), but the technical and time effort was huge, so that a faster method is needed for routine genotyping, such as the Region-Specific Extraction (RSE) method (Dapprich et al., 2016).

The specificity of a method such as the RSE method depends heavily on the specificity of the primers used. We selected primers deemed suitable for the RSE method from various publications and assured that these primers, could be used in a Capture Primer Set (CPS). The focus was on *E. multilocularis*-specific primers. To obtain a broader spectrum of primers to cover the mitogenome, primers specific to *Echinococcus* spp. were also included in this CPS. As the selected primers had been successfully used in other studies, we only looked at the site where they bind to the mtDNA and if there was a risk that they formed primer dimers (Bustin and Huggett, 2017). Checking primer-dimer formation is therefore important for efficiency (Owczarzy et al., 2008). The parameter considered for this is the ΔG value (Rychlik, 1995; SantaLucia and Hicks, 2004; Owczarzy et al., 2008; Hendling and Barišić, 2019). The ΔG value reflects the stability of binding oft two primer pairs. The lower the value, the greater the bond stability (increase the likelihood of dimer formation) (SantaLucia and Hicks, 2004). The primers used for the RSE method had ΔG values that were more positive than −9 kcal/mol (SantaLucia and Hicks, 2004; Johnston et al., 2019). Thus, the results of the primers selected for the RSE method in our study to type *E. multilocularis* using its mitogenome showed that the primers were suitable for the CPS as they are unlikely to form primer dimers (SantaLucia and Hicks, 2004; Johnston et al., 2019).

A control qPCR (Isaksson et al., 2014; Maksimov et al., 2019) was performed to determine the mtDNA quantity of *E. multilocularis* during the course of the experiments (dilution series to determine the analytical sensitivity, field samples for the detection of SNPs). The primers of this qPCR bind in a region of the mitogenome where also the *E. multilocularis*-specific primers of the Trachsel PCR (Trachsel et al., 2007), which were used in the CPS, also bind (Supplementary 2, Table S3, Figure S1), namely in the region of the *rrnS* gene. This is a rather solitary binding site of the CPS primers on the mitogenome compared to other sites in the mtDNA. This means that several primer pairs do not overlap in the *rrnS* gene, providing a realistic representation of the increase in the amount of mtDNA for *E. multilocularis* using the RSE method. This means that the increase in mtDNA can be recorded directly via the control qPCR.

The analytical sensitivity for the RSE method for the *E. multilocularis* mitogenome, determined using control qPCR (Isaksson et al., 2014; Maksimov et al., 2019) and the dilution series, has a ct value of <25. This value refers to the ‘before RSE’ samples to ensure that sufficient *E. multilocularis*-specific mtDNA is present in the sample to successfully perform the RSE method and NGS. The reason for restricting the results to the ct values is that the total DNA concentration measured with the NanoDrop technology can be inaccurate (especially for values <2 ng/μL; see NanoDrop 2000 User Manual, page 3–2, Measurement Ranges for Nucleic Acids) and may not provide reliable information on the concentration of *E. multilocularis* mtDNA specifically. Since the *E. multilocularis* mtDNA can vary in the original samples (‘before RSE’ samples), for example due to the condition and developmental status of the adult parasite specimens in the sample and also the state of decomposition of the final host Red Fox (e.g., activity of the DNAses in the host intestine), this can lead to a misjudgement of the *E. multilocularis* mtDNA concentration. We thus considered it useful to control the experiments using the ct values of the *E. multilocularis*-specific qPCR.

The dilution series was not only used to determine the ct value of the detection limit using control qPCR, but also to detect and analyse the correct positions and number of SNPs using the NGS data. The NGS results of the dilution series show that the positions of the SNPs remain stable up to a dilution of 1:4. At a dilution of 1:8, a decrease in the number of SNPs was observed, which is probably due to the lower read depth or the lack of a sufficient number of reads to detect the position and number of SNPs correctly (Figure 3C). Thus, the NGS data of the dilution series also show that a reliable statement about the SNPs present can be made for DNA quantities with a ct value <25.

With the knowledge of the results obtained by the dilution series experiment, the 14 field samples were assessed accordingly. All 14 samples (Em_1 to Em_14) had a ct value <25 of the ‘before RSE’ samples, so that a successful course of the experiments (RSE, NGS) could be assumed. The results of the samples showed that the detection limit was determined correctly, since the ct values of the ‘after REPLI-g’ values had decreased significantly (Table 2). This provided a high level of confidence for sufficient coverage and complete detection of all SNPs. The NGS data now showed that 100% coverage was achieved for the majority of field samples (Em_1 to Em_14) with a minimum read depth of 30X or 50X. The target average read depth of 100X was achieved for all samples. A higher depth was desirable, however, as this gave greater certainty that the found SNPs were not random. Although the percentage of mapped reads (% aligned) ranged between 0.1 and 88.8%, the median depth was still large enough to cover the genome at least 156-fold, which means that it is possible to assess the SNPs present as the number of reads per SNP site is still large enough.

Our data (Figure 4D) showed that many SNPs were monomorphic compared to the reference sequence in all analysed samples, but individual SNPs could also be detected in the respective mitogenome of a sample. Figure 4C also demonstrates that the number of SNPs varied between the samples. On the one hand, the present monomorphic SNPs do not contribute to the genetic variability (McCauley et al., 2007) and are usually excluded from the data analysis, but on the other hand, if a SNP is present in several individuals in a study, it can be assumed that it is not a sequencing error (Bansal et al., 2010). The presence of these monomorphic SNPs therefore increases the certainty that the mtDNA SNPs found for *E. multilocularis* using the RSE method are correct.

If the data (number of worms, red fox ID, number of SNPs and median depth) of the samples (Em_1 to Em_14) are compared, it becomes obvious that the number of SNPs does not seem to depend on the number of worms in the sample. For example, sample Em_7 has the largest number of SNPs (72) and sample Em_11 contained 66 SNPs but both samples consist of 8 worms. Sample Em_12 with 5 worms also had only 66 SNPs. In contrast, sample Em_10 (4 worms) contained 70 SNPs. One might thus hypothesise that the mean depth of sequencing can explain the differences in the numbers of SNPs observed, since lower coverage and read depth (e.g., <30X) may lead to random SNPs. However, this is not the case in our study, as the two samples Em_8 and Em_9 were taken from the same fox (Red Fox 1) and are thus technical replicates and yet they have significantly different read depths (199,718X for Em_8 and 156X for Em_9) but the same number (67) and type of SNPs (Figure 5 and Supplementary 2, Table S9). Therefore, if the read depth (of at least 100X) of the samples was not sufficient to map the SNPs correctly, we would have obtained a different number of SNPs for the two technical replicates. However, if we compare these two samples with a third sample (Em_4) from the same Red Fox (Red Fox 1), we detected two additional SNPs (69). Since we used a biological replicate in this case, i.e., different worms from the same fox, random point mutations in the genome of these two worms may explain the discrepancies (Knapp et al., 2009; Knapp et al., 2021). The same applies to the two samples (Em_1 and Em_6) from Red Fox 3, from which we concluded that the number and type of SNPs depended on the sample itself (possibly related to unrecognised technical issues) and the SNPs can be reproduced using the RSE method.

To ensure that our modified RSE method does not produce false SNP results, we compared the RSE data with whole genome sequencing (WGS) data. For this purpose, mitochondrial DNA (mtDNA) was extracted from the WGS data, and the SNPs were subsequently compared. Unfortunately, we were only able to perform this comparison with 4 out of 14 samples due to difficulties in obtaining sufficient DNA for the respective whole genome sequencing. The analysis of the mitochondrial whole genome revealed a match between the SNPs identified using the RSE method, with only one SNP being different. This finding confirms that the data generated by the RSE protocol is reliable and can serve as an alternative method for SNP detection in long nucleotide sequences (up to several thousands).

To assess intraspecific diversity, we applied a haplotype network algorithm to indicate the relatedness of the samples (Leigh and Bryant, 2015). Since we only have a small sample size and intended only testing the RSE method for *E. multilocularis* as a proof of principle, we cannot make an exact phylogenetic statement here, nevertheless we were able to distinguish 10 haplotypes within our 14 samples (Figure 2A). A higher diversity in the mitochondrial data was also recently reported by Bohard et al. (2023) with human cyst material, who also applied the complete mitogenome sequences in the study. An important finding of our results here is that we could roughly separate the worms from Red Foxes by region (i.e., the German Federal State in our case; Figure 2B), which may indicate that it is possible to map microdiversity at the local level. To confirm this, further studies are needed with a larger sample size from more regions.

Comparison with EmsB microsatellite analysis showed that the RSE method reflected a considerably higher diversity. Although the EmsB method is widely used (Bart et al., 2006; Knapp et al., 2008; Maillard et al., 2009; Herzig et al., 2021; Knapp et al., 2021; Umhang et al., 2021a) and shows a high resolution for a large-scale genotype distribution of *Echinococcus multilocularis*, this method is based on a single, but highly variable, small section of genomic DNA located on chromosome 5 (Bart et al., 2006; Knapp et al., 2007). It can therefore only reflect a small part of the possible variability. To find a higher diversity at the local level, it is necessary to broaden the focus and look at a larger section of DNA (mitogenome). Therefore, it should be important to re-determine and classify the genetic diversity of *E. multilocularis* based on the whole mitochondrial DNA (mtDNA) (or in combination with gDNA to better interpret the phylogeny of the parasite (Paijmans et al., 2013)). Yet, the use of mitochondrial DNA (mtDNA) seems to have advantages, compared to the EmsB method, since the mtDNA is haploid, occurs in a high copy number per cell (in two forms of mitochondria, aerobe and anaerobe (Martínez-González et al., 2022)), has a higher evolutionary rate than nuclear markers (such as EmsB), and does not show recombination (Paijmans et al., 2013; Spotin et al., 2015; Jastrzembski, 2017; Spotin et al., 2018).

Our data indicate that the EmsB analyses of the samples allow to assign them to two profiles, with two samples (from Brandenburg and Lower Saxony) showing the G profile. All other samples show a D profile (Supplementary 2, Figure S5). The *E. granulosus* sample, as the control outgroup, can be clearly distinguished from *E. multilocularis* and lies in the correct range for this parasite (Maillard et al., 2009). The data, at least for the samples from Brandenburg, agree with the results published by Herzig et al. (2021). Here, a grouping of samples with a G profile was also found in the north-western region of Brandenburg. In conclusion, we found a significantly higher diversity with the RSE method as compared to EmsB analysis.

The limitation of the study was the small sample size, which did not allow group genotypes in a phylogenetic tree. Spatial separation of the detected microdiversity could therefore not be demonstrated. More samples need to be tested for this type of analysis. Nevertheless, with these few samples we were able to clearly show that there must be a microdiversity of *Echinococcus multilocularis* that is greater than the two profiles previously detected with EmsB microsatellites in this study. Other studies have already come to similar conclusions (Laurimäe et al., 2018; Bohard et al., 2023).

In conclusion, the results demonstrate that it is possible to use the RSE method to detect and analyse the entire mitogenome of *E. multilocularis* for genotyping purposes. Even with a few samples, we were able to show that a higher intraspecific diversity was found with the RSE method compared to the widely used EmsB microsatellite analysis. Comparison with WGS data showed that the accuracy and validity of the RSE method delivers reliable results and can be applied as an alternative sequencing and typing method.

## Data Availability

The datasets presented in this study can be found in online repositories. The names of the repository/repositories and accession number(s) can be found at: https://www.ebi.ac.uk/ena, PRJEB74928.

## Footnotes

^1^ https://www.bioinformatics.babraham.ac.uk/projects/fastqc/

^2^ https://parasite.wormbase.org
