# Supplementary material for: Typing of Echinococcus multilocularis by Region-Specific Extraction and Next-Generation Sequencing of the mitogenome
Source: Front Microbiol. 2025 Feb 28;16:1535628. doi: 10.3389/fmicb.2025.1535628 (PMC11906691; doi:10.3389/fmicb.2025.1535628)
Supplement: Supplementary file 1 [file Data_Sheet_1.pdf]

Table S1: List of reagents, materials, software, and equipment.

Method		Designation	Composition	Product name	Manufacturer	Order no.	Further procedure
**Sedimentation and counting technique (SCT) method**	**Reagents**	0.9% saline solution	9 g NaCl	Natriumchlorid >99,5%, p.a., ACS, ISO	Carl Roth GmbH + Co. KG	3957.1	-
			ad. 1000 ml double-distilled water	Milli-Q® Advantage A10 Water Purification System	Merck KGaA	Z00Q0V0WW	
		4% Helipur® solution	40 ml Helipur®	Helipur®	B. Braun Melsungen AG	8505202	-
			960 ml distilled water	tap water	-	-	
		3.75% sodium hypochlorite	312.5 ml Sodium hypochlorite	Sodium hypochlorite solution, 12 % Cl, Biocide Grade	Carl Roth GmbH + Co. KG	0078.1	-
			687.5 ml distilled water	tap water	-	-	
	**Consumables**	50 ml Falcon tube	-	Falcon® 50 ml conical centrifuge tubes	Fisher Scientific GmbH	10788561	-
		Square polystyrene petri dish	-	Square polystyrene petri dish non-sterile, 120 x 120 x 17 mm	Greiner Bio-One International GmbH	EL50.1	-
		1.5 ml Eppendorf tubes	-	Eppendorf Safe-Lock Tubes, 1.5 mL, Eppendorf Quality™	Eppendorf SE	30120086	-
	**Equipment**	Binocular	-	Nikon SMZ 745T	Nikon Metrology GmbH	-	-
		Precision scale	-	Precision scale PNS/PNJ	KERN & SOHN GmbH	PNJ 3000-2M##3	-
		measuring cylinder	-	500 ml Kartell™ (TPX™) PMP Graduated Conical Measures	Kartell S.p.A. – LABWARE Division	10050941	-
		- 80°C freezer	-	Ultra-Low Temperature Freezer C760-86 Innova®	New Brunswick™ an Eppendorf Company	U9410-0002	-
		scissors	-	microscope scissors (curved, 105 mm)	A. Hartenstein	SN02	-
		forceps	-	splinter forceps (straight, 120 mm)	A. Hartenstein	PZ02	-
		microscope slides	-	microscope slides thickness approx. 1 mm with cut edges (plain)	Paul Marienfeld GmbH & Co. KG	1000000	-
**Collection of the parasites from the sediment of Red Fox intestinal mucosa**	**Reagents**	0.1x TE buffer	50 µl ROTI®Stock 100x TE, 100x conc.	ROTI®Stock 100x TE, 100x conc., BioScience Grade, ready-to-use, steam sterilized	Carl Roth GmbH + Co. KG	1052.1	-
			49.95 ml nuclease-free water	Nuclease-Free Water, for Molecular Biology	Sigma-Aldrich (subsidiary of Merck KGaA)	W4502-1L	-
	**Consumables**	50 ml Falcon tube	-	Falcon® 50 ml conical centrifuge tubes	Fisher Scientific GmbH	10788561	-
		pipette filter tips	-	Filter tip PP	nerbe plus GmbH & Co. KG	07-642-8300	-
	**Equipment**	pipettes	-	Single-channel micropipette Transferpette® S, adjustable, DE-M	BRAND GMBH + CO KG	705874	-
		scalpels	-	Scalpel Blades Figure No.11. for single use	InstrumenteNRW	100xx SK11-005	-
**Digestion of the parasites**	**Reagents**	5 M NaOH (100 ml)	100 ml A bidest.	Milli-Q® Advantage A10 Water Purification System	Merck KGaA	Z00Q0V0WW	the approach was done on ice
			20 g NaOH	Sodium Hydroxide pellets (USP-NF, BP, Ph. Eur.) pure, pharma grade	AppliChem GmbH	141687	
		2 N HCl (50 ml)	41.71 ml A. bidest.	Milli-Q® Advantage A10 Water Purification System	Merck KGaA	Z00Q0V0WW	-
			8.29 ml 37% HCl	Hydrochloric Acid 37% (Reag. USP) for analysis, ACS, ISO	AppliChem GmbH	131020	-
		25 % HCl (50 ml)	16.22 ml A. bidest.	Milli-Q® Advantage A10 Water Purification System	Merck KGaA	Z00Q0V0WW	-
			33.78 ml 37% HCl	Hydrochloric Acid 37% (Reag. USP) for analysis, ACS, ISO	AppliChem GmbH	131020	-
		0.5 M EDTA (pH 8.0)	46.53 g EDTA	Ethylendiamin-tetraessigsäure Dinatriumsalz Dihydrat ≥ 99 %, p.a., ACS	Carl Roth GmbH + Co. KG	8043.2	-
			approx. 200 ml A bidest.	Milli-Q® Advantage A10 Water Purification System	Merck KGaA	Z00Q0V0WW	-
			5 M NaOH	self made	-	-	add until the EDTA has dissolved
			25% HCl	self made	-	-	for adjustment of pH 8.0
			A. bidest.	Milli-Q® Advantage A10 Water Purification System	Merck KGaA	Z00Q0V0WW	fill up to 250 ml
		250 mM EDTA (pH 8.0)	100 ml 0.5 M EDTA (pH 8.0)	self made	-	-	-
			100 ml A. bidest.	Milli-Q® Advantage A10 Water Purification System	Merck KGaA	Z00Q0V0WW	-
			25% HCl	self made	-	-	for adjustment of pH 8.0
		1 M Tris-HCl (pH 8.0)	12.1 g Tris	UltraPure™ Tris buffer	Invitrogen™ Thermo Fisher Scientific Inc.	15504020	-
			approx. 70 ml A. bidest.	Milli-Q® Advantage A10 Water Purification System	Merck KGaA	Z00Q0V0WW	-
			25% HCl	self made	-	-	for adjustment of pH 8.0
			A. bidest.	Milli-Q® Advantage A10 Water Purification System	Merck KGaA	Z00Q0V0WW	fill up to 100 ml
		digestion buffer (not older than 2 weeks)	0.58 g NaCl (100 mM)	Sodium chloride (NaCl)	Carl Roth GmbH+Co. KG	3957.1	-
			10 ml 250 mM EDTA (pH 8.0; 25 mM)	self made	-	-	-
			0.5 g SDS (0.5%)	SDS ultra pure, ≥99 %, for electrophoresis, biochemistry and molecular biology	Carl Roth GmbH + Co. KG	2326.2	-
			1 ml 1 M Tris-HCl (pH 8.0; 10 mM)	self made	-	-	-

**Digestion of the parasites**	**Reagents**	digestion buffer (not older than 2 weeks)	ad 100 ml A. bidest	Milli-Q® Advantage A10 Water Purification System	Merck KGaA	Z00Q0V0WW	-
			1 µl Proteinase K to 200 µl digestion buffer (0.1 mg/ml)	Macherey-Nagel™ Proteinase K (20 mg/ml)	Macherey-Nagel™	11912312	add shortly before use
	**Equipment**	centrifuge	-	Eppendorf Centrifuge 5430R	Eppendorf SE	5428000205	-
		pH meter	-	766 Laboratory pH Meter	Knick elektronische Meßgeräte GmbH & Co.	ZU 6956 & ZU 6955	-
		thermomixer	-	Eppendorf Thermomixer comfort	Eppendorf AG	5355 000.11	-
		precision scale	-	Precision scale PNS/PNJ	KERN & SOHN GmbH	PNJ 3000-2M##3	-
**DNA extraction with phenol-chloroform method**	**Reagents**	Phenol-chloroform	-	Phenol:Chloroform:Isoamyl Alcohol 25:24:1	Sigma-Aldrich (subsidiary of Merck KGaA)	P2069-100ML	-
		2 N HCl (50 ml)	41.71 ml A. bidest.	Milli-Q® Advantage A10 Water Purification System	Merck KGaA	Z00Q0V0WW	-
			8.29 ml 37% HCl	Hydrochloric Acid 37% (Reag. USP) for analysis, ACS, ISO	AppliChem GmbH	131020	-
		10 mM Tris-HCl (pH 8.5; Elution buffer = EB)	0.121 g Tris	UltraPure™ Tris buffer	Invitrogen™ Thermo Fisher Scientific Inc.	15504020	-
			95 ml nuclease-free water	Nuclease-Free Water, for Molecular Biology	Sigma-Aldrich (subsidiary of Merck KGaA)	W4502-1L	-
			2 N HCl	self made	-	-	for adjustment of pH 8.5
			ad 100 ml nuclease-free water	Nuclease-Free Water, for Molecular Biology	Sigma-Aldrich (subsidiary of Merck KGaA)	W4502-1L	-
		Ammonium acetate (7.5 M)	-	Ammonium acetate solution for molecular biology 7,5 M	Sigma-Aldrich (subsidiary of Merck KGaA)	A2706-500ml	-
		glycogen (20 mg/ml)	20 mg glycogen	Glycogen from oyster, ≥75% dry basis	Sigma-Aldrich (subsidiary of Merck KGaA)	G8751-5G	-
			1 ml nuclease-free water	Nuclease-Free Water, for Molecular Biology	Sigma-Aldrich (subsidiary of Merck KGaA)	W4502-1L	-
		100% ethanol	-	Ethanol ROTIPURAN® ≥ 99.8%, p.a.	Carl Roth GmbH+Co. KG	9065.4	-
		80% ethanol	80 ml 100% ethanol	Ethanol ROTIPURAN® ≥ 99.8%, p.a.	Carl Roth GmbH+Co. KG	9065.4	-
			20 ml nuclease-free water	Nuclease-Free Water, for Molecular Biology	Sigma-Aldrich (subsidiary of Merck KGaA)	W4502-1L	-
	**Equipment**	chemical fume cupboard	-	Secuflow mc6® das Laborsystem	WALDNER Laboreinrichtungen GmbH & Co. KG	-	for working with phenol-chloroform
		vortexer	-	Heidolph REAX 2000	Heidolph Instruments GmbH & Co. KG	-	-
		centrifuge 1	-	Eppendorf Centrifuge 5430R	Eppendorf SE	5428000205	-
		centrifuge 2	-	Hettich Zentrifugen Mikro 200	Andreas Hettich GmbH & Co. KG	2400	-
		pH meter	-	766 Laboratory pH Meter	Knick elektronische Meßgeräte GmbH & Co.	ZU 6956 & ZU 6955	-
		thermomixer	-	Eppendorf Thermomixer comfort	Eppendorf AG	5355 000.11	-
		precision scale	-	Precision scale PNS/PNJ	KERN & SOHN GmbH	PNJ 3000-2M##3	-
		- 20 °C freezer	-	G 5216 ProfiLine	Liebherr-Hausgeräte GmbH	-	-
		refrigerator	-	LKexv Modelle	Liebherr-Hausgeräte GmbH	-	
**Digestion and DNA extraction of the cyst material**	**Equipment**	scissors	-	microscope scissors (curved, 105 mm)	A. Hartenstein	SN02	-
		forceps	-	splinter forceps (straight, 120 mm)	A. Hartenstein	PZ02	-
		precision scale	-	Precision scale PNS/PNJ	KERN & SOHN GmbH	PNJ 3000-2M##3	-
		thermomixer	-	Eppendorf Thermomixer comfort	Eppendorf AG	5355 000.11	-
		centrifuge	-	Eppendorf Centrifuge 5430R	Eppendorf SE	5428000205	-
		vortexer	-	Heidolph REAX 2000	Heidolph Instruments GmbH & Co. KG	-	-
		pipettes	-	Single-channel micropipette Transferpette® S, adjustable, DE-M	BRAND GMBH + CO KG	705874	-
	**Materials**	NucleoSpin Tissue kit	-	NucleoSpin Tissue, Mini kit for DNA from cells and tissue	MACHEREY-NAGEL GmbH & Co. KG	REF 740952.250	-
		1.5 ml Eppendorf tubes	-	Eppendorf Safe-Lock Tubes, 1.5 mL, Eppendorf Quality™	Eppendorf SE	30120086	-

**Digestion and DNA extraction of the cyst material**	**Materials**	pipette filter tips	-	Filter tip PP	nerbe plus GmbH & Co. KG	07-642-8300	-
	**Reagents**	nuclease-free water	-	Nuclease-Free Water, for Molecular Biology	Sigma-Aldrich (subsidiary of Merck KGaA)	W4502-1L	-
**Capture Primer Set (CPS)**	**Reagents**	primers	-	name depending on the primer	metabion international AG	-	-
		nuclease-free water	-	Nuclease-Free Water, for Molecular Biology	Sigma-Aldrich (subsidiary of Merck KGaA)	W4502-1L	-
	**Software**	primer dimers detection	-	http://www.primer-dimer.com/	Australian Institute for BioEngineering and Nanotechnology (AIBN), Centre for Personalised Nanomedicine, Trau Lab, Jennifer Lu and Darren Korbie	-	-
		Geneious Prime®	-	Geneious Prime® 2021.0.1 (Build 2020-12-01 11:16, Java Version 11.0.9+11 (64 bit))	Biomatters Limited	-	-
**TaqMan® real-time qPCRs for monitoring the results**	**Reagents**	primers	-	name depending on the primer	metabion international AG	-	-
		probes	-	name depending on the probe	metabion international AG	-	-
		nuclease-free water	-	Nuclease-Free Water, for Molecular Biology	Sigma-Aldrich (subsidiary of Merck KGaA)	W4502-1L	-
	**Software**	Excel	-	Office Professional Plus 2019, Excel	Microsoft Corporation	-	-
		GraphPad Prism		Prism 8 for Windows 64-bit (version 8.1.0 (325))	GraphPad Software, Inc.	-	-
		Bio-Rad CFX Maestro	-	Bio-Rad CFX Maestro 1.1 (version 4.1.2433.1219)	Bio-Rad Laboratories, Inc.	-	-
	**Materials**	1.5 ml Eppendorf tubes	-	Eppendorf Safe-Lock Tubes, 1.5 mL, Eppendorf Quality™	Eppendorf SE	30120086	-
		PCR plate sealing films	-	Microseal 'B' PCR Plate Sealing Film, adhesive, optical	Bio-Rad Laboratories, Inc.	MSB1001	-
		96-well plates	-	Multiplate™ 96-Well PCR Plates, low profile, unskirted, clear	Bio-Rad Laboratories, Inc.	MLL9601	-
		pipette filter tips		Filter tip PP	nerbe plus GmbH & Co. KG	-	-
		QuantiTect Multiplex PCR NoROX Kit	-	QuantiTect Multiplex PCR NoROX Kit (200)	QIAGEN	204743	-
	**Equipment**	pipettes	-	Single-channel micropipette Transferpette® S, adjustable, DE-M	BRAND GMBH + CO KG	-	-
		thermal cycler	-	C1000 Touch™ Thermal Cycler with CFX96™ Optical Reaction Modul	Bio-Rad Laboratories, Inc.	-	-
**RSE method**	**Reagents**	nuclease-free water	-	Nuclease-Free Water, for Molecular Biology	Sigma-Aldrich (subsidiary of Merck KGaA)	W4502-1L	-
	**Materials**	RSE kit	-	Region Specific Extraction (RSE)	Generation Biotech, LLC	-	-
		REPLI-g Mini Kit	-	REPLI-g Mini Kit (25)	QIAGEN	150023	-
		pipette filter tips	-	Filter tip PP	nerbe plus GmbH & Co. KG	-	-
		1.5 ml Eppendorf tubes	-	Eppendorf Safe-Lock Tubes, 1.5 mL, Eppendorf Quality™	Eppendorf SE	30120086	-
	**Equipment**	thermomixer	-	Eppendorf Thermomixer comfort	Eppendorf AG	5355 000.11	-
		water bath 1	-	Julabo 19 ED	JULABO GmbH	-	-
		water bath 2	-	Shaking water baths, VLSB12	VWR International, LLC	462-0493	-
		vortexer	-	Test tube shakers ROTILABO® Mini Vortex	ROTH SELECTION	HXH6.1	-
		magnet rack	-	Magna GrIP Rack (8 well) to hold 1.5 - 2 ml tubes	Sigma-Aldrich (subsidiary of Merck KGaA)	20-400	-
		pipettes	-	Single-channel micropipette Transferpette® S, adjustable, DE-M	BRAND GMBH + CO KG	-	-
		UV Pipette Carousel	-	nUVa Clean - UV Pipette Carousel	Biozym Scientific GmbH	55P5590-E	-
		PCR cabinet	-	DNA/ RNA UV-Clean UVC/T-M-AR	LTF Labortechnik GmbH & Co. KG	-	-
**Dilution series**	**Reagents**	primers	-	name depending on the primer	metabion international AG	-	-
		probes	-	name depending on the probe	metabion international AG	-	-
		nuclease-free water	-	Nuclease-Free Water, for Molecular Biology	Sigma-Aldrich (subsidiary of Merck KGaA)	W4502-1L	-
	**Software**	Excel	-	Office Professional Plus 2019, Excel	Microsoft Corporation	-	-
		GraphPad Prism	-	Prism 8 for Windows 64-bit (version 8.1.0 (325))	GraphPad Software, Inc.	-	-

	**Software**	Bio-Rad CFX Maestro	-	Bio-Rad CFX Maestro 1.1 (version 4.1.2433.1219)	Bio-Rad Laboratories, Inc.	-	-
**Dilution series**	**Materials**	1.5 ml Eppendorf tubes	-	Eppendorf Safe-Lock Tubes, 1.5 mL, Eppendorf Quality™	Eppendorf SE	30120086	-
		PCR plate sealing films	-	Microseal 'B' PCR Plate Sealing Film, adhesive, optical	Bio-Rad Laboratories, Inc.	MSB1001	-
		96-well plates	-	Multiplate™ 96-Well PCR Plates, low profile, unskirted, clear	Bio-Rad Laboratories, Inc.	MLL9601	-
		pipette filter tips		Filter tip PP	nerbe plus GmbH & Co. KG	-	-
		QuantiTect Multiplex PCR NoROX Kit	-	QuantiTect Multiplex PCR NoROX Kit (200)	QIAGEN	204743	-
	**Equipment**	NanoDrop	-	NanoDrop™ 2000 spectrophotometer	Thermo Fisher Scientific Inc.	ND-2000	
		pipettes	-	Single-channel micropipette Transferpette® S, adjustable, DE-M	BRAND GMBH + CO KG	-	-
		thermal cycler	-	C1000 Touch™ Thermal Cycler with CFX96™ Optical Reaction Modul	Bio-Rad Laboratories, Inc.	-	-
**EmsB microsatellite analysis**	**Reagents**	primers	-	name depending on the primer	metabion international AG	-	-
		probes	-	name depending on the probe	metabion international AG	-	-
		nuclease-free water	-	Nuclease-Free Water, for Molecular Biology	Sigma-Aldrich (subsidiary of Merck KGaA)	W4502-1L	-
		Taq Polymerase	-	Platinum Taq Polymerase	invitrogen™ by Thermo Fisher Scientific Inc.	10966018	with 10x buffer & Mg_2Cl (50mM)
		dNTPs	-	dNTP Set (4 x 0.5 ml, each 50 mM)	stratec molecular GmbH (became to Invitek Molecular)	3020301200	-
		DMSO	-	Dimethylsulfoxid, BioUltra, for molecular biology, ≥99.5% (GC)	Sigma-Aldrich (subsidiary of Merck KGaA)	41639-100ML	-
		Formamide	-	Hi-Di™ Formamide	Applied Biosystems™ by Thermo Fisher Scientific	4311320	-
		ROX 500	-	GeneScan™ 500 ROX™ dye Size Standard	Applied Biosystems™ by Thermo Fisher Scientific	401734	-
	**Materials**	1.5 ml Eppendorf tubes	-	Eppendorf Safe-Lock Tubes, 1.5 mL, Eppendorf Quality™	Eppendorf SE	30120086	-
		PCR plate sealing films	-	Microseal 'B' PCR Plate Sealing Film, adhesive, optical	Bio-Rad Laboratories, Inc.	MSB1001	-
		96-well plates	-	Multiplate™ 96-Well PCR Plates, low profile, unskirted, clear	Bio-Rad Laboratories, Inc.	MLL9601	-
		pipette filter tips		Filter tip PP	nerbe plus GmbH & Co. KG	-	-
	**Equipment**	thermomixer	-	Eppendorf Thermomixer comfort	Eppendorf AG	5355 000.11	-
		thermal cycler	-	C1000 Touch™ Thermal Cycler with 96-Well Fast Reaction Module	Bio-Rad Laboratories, Inc.	-	-
		Genetic Analyser	-	Applied Biosystems Hitachi 3500 Genetic Analyzer instrument	Applied Biosystems™ by Thermo Fisher Scientific	-	-
		pipettes	-	Single-channel micropipette Transferpette® S, adjustable, DE-M	BRAND GMBH + CO KG	-	-
	**Software**	GeneMapper™	-	GeneMapper™ (version 5.0)	Applied Biosystems™ by Thermo Fisher Scientific	4366847	-
		Excel	-	Office Professional Plus 2019, Excel	Microsoft Corporation	-	-
		R	-	Cran R 4.1.2 + Rtools 4.0v2	Free Software Foundation, Inc.	-	-
		RStudio	-	version 2021.9.1-372	Posit Software, PBC formerly RStudio, PBC	-	-
		R package pvclust	-	version 2.2-0	https://CRAN.R-project.org/package=pvclust	-	-
**Next-Generation Sequencing (NGS)**	**Equipment**	MiSeq	-	Illumina MiSeq™	Illumina, Inc.	-	sent to NGS unit of the Institute of Virus Diagnostics, Friedrich-Loeffler-Institut, Greifswald – Insel Riems, Germany
		NovaSeq	-	Illumina NovaSeq™ 6000	Illumina, Inc.	-	sent to Eurofins Genomics Germany GmbH
