# Supplementary material for: Typing of Echinococcus multilocularis by Region-Specific Extraction and Next-Generation Sequencing of the mitogenome
Source: Front Microbiol. 2025 Feb 28;16:1535628. doi: 10.3389/fmicb.2025.1535628 (PMC11906691; doi:10.3389/fmicb.2025.1535628)
Supplement: Supplementary file 2 [file Data_Sheet_2.pdf]

Table S2: Samples of *E. multilocularis*, their origin and use in different experiments.

Sample ID	Country	Federal state	Host species	Red Fox ID	Origin of material	Parasite	No. of adult specimens	Dilution step	RSE	WGS	EmsB
**Em_1**	Germany	Lower Saxony	Red Fox (*Vulpes vulpes*)	Red Fox 3	Intestinal mucosa	*E. multilocularis*	1	-	X	-	X
**Em_2**	Germany	Thuringia	Red Fox (*Vulpes vulpes*)	Red Fox 2	Intestinal mucosa	*E. multilocularis*	1	-	X	-	X
**Em_3**	Germany	Brandenburg	Red Fox (*Vulpes vulpes*)	Red Fox 5	Intestinal mucosa	*E. multilocularis*	1 worm without proglottids	-	X	-	X
**Em_4**	Germany	Thuringia	Red Fox (*Vulpes vulpes*)	Red Fox 1	Intestinal mucosa	*E. multilocularis*	1 worm without proglottids	-	X	-	X
**Em_5**	Germany	Brandenburg	Red Fox (*Vulpes vulpes*)	Red Fox 4	Intestinal mucosa	*E. multilocularis*	1 worm without proglottids	-	X	-	X
**Em_6**	Germany	Lower Saxony	Red Fox (*Vulpes vulpes*)	Red Fox 3	Intestinal mucosa	*E. multilocularis*	1 worm without proglottids	-	X	-	X
**Em_7**	Germany	Thuringia	Red Fox (*Vulpes vulpes*)	Red Fox 6	Intestinal mucosa	*E. multilocularis*	8	-	X	-	X
**Em_8**	Germany	Thuringia	Red Fox (*Vulpes vulpes*)	Red Fox 1	Intestinal mucosa	*E. multilocularis*	1	-	X	-	X
**Em_9**	Germany	Thuringia	Red Fox (*Vulpes vulpes*)	Red Fox 1	Intestinal mucosa	*E. multilocularis*	1	-	X	X	X
**Em_10**	Germany	Thuringia	Red Fox (*Vulpes vulpes*)	Red Fox 7	Intestinal mucosa	*E. multilocularis*	4	-	X	X	X
**Em_11**	Germany	Thuringia	Red Fox (*Vulpes vulpes*)	Red Fox 8	Intestinal mucosa	*E. multilocularis*	8	-	X	X	X
**Em_12**	Germany	Brandenburg	Red Fox (*Vulpes vulpes*)	Red Fox 4	Intestinal mucosa	*E. multilocularis*	5	-	X	X	X
**Em_13**	Germany	Thuringia	Red Fox (*Vulpes vulpes*)	Red Fox 9	Intestinal mucosa	*E. multilocularis*	1	-	X	-	X
**Em_14**	Germany	Thuringia	Red Fox (*Vulpes vulpes*)	Red Fox 2	Intestinal mucosa	*E. multilocularis*	1	undiluted	X	-	X
**Em_15**	Germany	Thuringia	Red Fox (*Vulpes vulpes*)	Red Fox 2	Intestinal mucosa	*E. multilocularis*	1	1:2	X	-	-
**Em_16**	Germany	Thuringia	Red Fox (*Vulpes vulpes*)	Red Fox 2	Intestinal mucosa	*E. multilocularis*	1	1:4	X	-	-
**Em_17**	Germany	Thuringia	Red Fox (*Vulpes vulpes*)	Red Fox 2	Intestinal mucosa	*E. multilocularis*	1	1:8	X	-	-
**Em_18**	Germany	Thuringia	Red Fox (*Vulpes vulpes*)	Red Fox 2	Intestinal mucosa	*E. multilocularis*	1	1:16	X	-	-
**Em_19**	Germany	Thuringia	Red Fox (*Vulpes vulpes*)	Red Fox 2	Intestinal mucosa	*E. multilocularis*	1	1:32	X	-	-
**Em_20**	Germany	Thuringia	Red Fox (*Vulpes vulpes*)	Red Fox 2	Intestinal mucosa	*E. multilocularis*	1	1:64	X	-	-
**Eg_1**	Kenya	NA	Cattle	-	Cyst material	*Echinococcus granulosus* (Gx)	-	-	-	-	X

Legend:

RSE Region-Specific Extraction method

WGS Mitogenome from Whole-Genome Sequencing

EmsB A tandem repeated multi-loci microsatellite for *E. multilocularis* (on chromosome 5)

X The analysis was carried out with this sample

NA Information not available

Table S3: All Primers and Probes (mtDNA = mitochondrial DNA, gDNA = genomic DNA).

Designation	Primer	Probe	Direction	Sequence (5'-3')	Genome	Gene	Target species	Reference
**EM-H15_F**	x		forward	CCA TAT TAC AAC AAT ATT CCT ATC	mtDNA	*rrnS*	*E. multilocularis*	Stieger et al. (2002), Trachsel et al. (2007)
**EM-H17_R**	x		reverse	GTG AGT GAT TCT TGT TAG GGG AAG	mtDNA	*rrnS*	*E. multilocularis*	Stieger et al. (2002), Trachsel et al. (2007)
**Cest1**	x		forward	TGC TGA TTT GTT AAA GTT AGT GAT C	mtDNA	*nad* 1	*E. multilocularis*	Trachsel et al. (2007)
**Cest2**	x		reverse	CAT AAA TCA ATG GAA ACA ACA ACA AG	mtDNA	*nad* 1	*E. multilocularis*	Trachsel et al. (2007)
**JB11.5**	x		forward	TTA TGG TAG ATA TTA TAG	mtDNA	*nad* 1	*Echinococcus* sp.	Bowles and McManus (1993)
**JB12.5**	x		reverse	CAC ACA CAT AAA ACA AGC	mtDNA	*nad* 1	*Echinococcus* sp.	Bowles and McManus (1993)
**F/CO1**	x		forward	TTG AAT TTG CCA CGT TTG AAT GC	mtDNA	*cox* 1	*Echinococcus* sp.	Xiao et al. (2003)
**R/CO1**	x		reverse	GAA CCT AAC GAC ATA ACA TAA TGA	mtDNA	*cox* 1	*Echinococcus* sp.	Xiao et al. (2003)
**atp6st for**	x		forward	GTT GTC CGT TAA ATT TCT TTT AGC	mtDNA	*atp* 6	*Echinococcus* sp.	Herzig et al. (2021)
**atp6st rev**	x		reverse	GGA ATA ATT GCT AAC CTA CAC AAC	mtDNA	*atp* 6	*Echinococcus* sp.	Herzig et al. (2021)
**EmMGBF**	x		forward	GTG CTG CTY ATA AGA GTT TTT G	mtDNA	*rrnS*	*E. multilocularis*	Isaksson et al. (2014)
**EmMGBR**	x		reverse	CTA TTA AGT CCT AAA CAA TAC CAT A	mtDNA	*rrnS*	*E. multilocularis*	Isaksson et al. (2014)
**EmMGBP**		x		VIC-ACA ACA ATA TTC CTA TCA ATG T-MGB	mtDNA	*rrnS*	*E. multilocularis*	Isaksson et al. (2014)
**EmsB A**	x		forward	6-FAM-GTG TGG ATG AGT GTG CCA TC	gDNA	-	*Echinococcus* sp.	Bart et al. (2006)
**EmsB C**	x		reverse	CCA CCT TCC CTA CTG CAA TC	gDNA	-	*Echinococcus* sp.	Bart et al. (2006)

Figure S1: Binding sites of used five primer pairs (in green) for Capture Primer Set (CPS) on the mitogenome of *Echinococcus multilocularis* reference genome (13,738 bp, GenBank: NC_000928). Circular genome with genes (in grey). Created with Geneious Prime®.

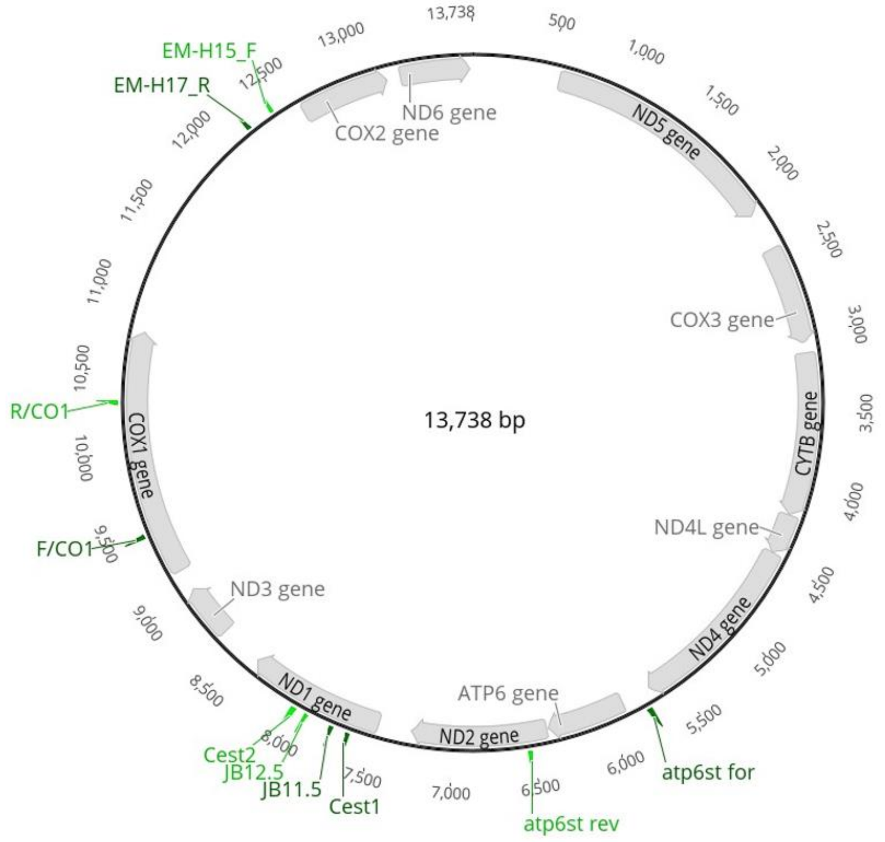

Table S4: Results of the primer-dimer detection using the website http://www.primer-dimer.com/ (access: 24.11.2021). A multiplex analysis (each primer is screened against all other primers) was performed. The ΔG value reflects the stability of binding of two primer pairs. The lower the value, the greater the bond stability (increases the likelihood of dimer formation). Values above - 9 kcal/mol are generally considered acceptable.

Forward Primer Name	Forward Primer Seq	Reverse Primer Name	Reverse Primer Seq	Structure	ΔG [kcal/mol]
EM-H15_F	CCATATTACAACAATATTCCTATC	Cest1	TGCTGATTTGTTAAAGTTAGTGATC	heterodimer	-6.78
EM-H17_R	GTGAGTGATTCTTGTTAGGGGAAG	Cest1	TGCTGATTTGTTAAAGTTAGTGATC	heterodimer	-6.78
Cest1	TGCTGATTTGTTAAAGTTAGTGATC	Cest2	CATAAATCAATGGAAACAACAACAAG	heterodimer	-6.78
Cest1	TGCTGATTTGTTAAAGTTAGTGATC	JB11.5	TTATGGTAGATATTATAG	heterodimer	-6.78
Cest1	TGCTGATTTGTTAAAGTTAGTGATC	JB12.5	CACACACATAAAACAAGC	heterodimer	-6.78
Cest1	TGCTGATTTGTTAAAGTTAGTGATC	F/CO1	TTGAATTTGCCACGTTTGAATGC	heterodimer	-6.78
Cest1	TGCTGATTTGTTAAAGTTAGTGATC	R/CO1	GAACCTAACGACATAACATAATGA	heterodimer	-6.78
Cest1	TGCTGATTTGTTAAAGTTAGTGATC	atp6st for	GTTGTCCGTTAAATTTCTTTTAGC	heterodimer	-6.78
Cest1	TGCTGATTTGTTAAAGTTAGTGATC	atp6st rev	GGAATAATTGCTAACCTACACAAC	heterodimer	-6.78
EM-H17_R	GTGAGTGATTCTTGTTAGGGGAAG	Cest2	CATAAATCAATGGAAACAACAACAAG	heterodimer	-5.09
EM-H15_F	CCATATTACAACAATATTCCTATC	F/CO1	TTGAATTTGCCACGTTTGAATGC	heterodimer	-3.65
EM-H17_R	GTGAGTGATTCTTGTTAGGGGAAG	F/CO1	TTGAATTTGCCACGTTTGAATGC	heterodimer	-3.65
Cest2	CATAAATCAATGGAAACAACAACAAG	F/CO1	TTGAATTTGCCACGTTTGAATGC	heterodimer	-3.65
JB11.5	TTATGGTAGATATTATAG	F/CO1	TTGAATTTGCCACGTTTGAATGC	heterodimer	-3.65
JB12.5	CACACACATAAAACAAGC	F/CO1	TTGAATTTGCCACGTTTGAATGC	heterodimer	-3.65
F/CO1	TTGAATTTGCCACGTTTGAATGC	R/CO1	GAACCTAACGACATAACATAATGA	heterodimer	-3.65
F/CO1	TTGAATTTGCCACGTTTGAATGC	atp6st for	GTTGTCCGTTAAATTTCTTTTAGC	heterodimer	-3.65
F/CO1	TTGAATTTGCCACGTTTGAATGC	atp6st rev	GGAATAATTGCTAACCTACACAAC	heterodimer	-3.65
EM-H15_F	CCATATTACAACAATATTCCTATC	atp6st for	GTTGTCCGTTAAATTTCTTTTAGC	heterodimer	-3.48
EM-H17_R	GTGAGTGATTCTTGTTAGGGGAAG	atp6st for	GTTGTCCGTTAAATTTCTTTTAGC	heterodimer	-3.48
Cest2	CATAAATCAATGGAAACAACAACAAG	atp6st for	GTTGTCCGTTAAATTTCTTTTAGC	heterodimer	-3.48
JB11.5	TTATGGTAGATATTATAG	atp6st for	GTTGTCCGTTAAATTTCTTTTAGC	heterodimer	-3.48
JB12.5	CACACACATAAAACAAGC	atp6st for	GTTGTCCGTTAAATTTCTTTTAGC	heterodimer	-3.48
R/CO1	GAACCTAACGACATAACATAATGA	atp6st for	GTTGTCCGTTAAATTTCTTTTAGC	heterodimer	-3.48
atp6st for	GTTGTCCGTTAAATTTCTTTTAGC	atp6st rev	GGAATAATTGCTAACCTACACAAC	heterodimer	-3.48
EM-H17_R	GTGAGTGATTCTTGTTAGGGGAAG	JB12.5	CACACACATAAAACAAGC	heterodimer	-3.39
EM-H15_F	CCATATTACAACAATATTCCTATC	JB12.5	CACACACATAAAACAAGC	heterodimer	-2.8
Cest2	CATAAATCAATGGAAACAACAACAAG	JB12.5	CACACACATAAAACAAGC	heterodimer	-2.8
JB11.5	TTATGGTAGATATTATAG	JB12.5	CACACACATAAAACAAGC	heterodimer	-2.8
JB12.5	CACACACATAAAACAAGC	R/CO1	GAACCTAACGACATAACATAATGA	heterodimer	-2.8
JB12.5	CACACACATAAAACAAGC	atp6st rev	GGAATAATTGCTAACCTACACAAC	heterodimer	-2.8
EM-H15_F	CCATATTACAACAATATTCCTATC	R/CO1	GAACCTAACGACATAACATAATGA	heterodimer	-2.49
EM-H15_F	CCATATTACAACAATATTCCTATC	EM-H17_R	GTGAGTGATTCTTGTTAGGGGAAG	heterodimer	-2.06
EM-H17_R	GTGAGTGATTCTTGTTAGGGGAAG	atp6st rev	GGAATAATTGCTAACCTACACAAC	heterodimer	-1.85
EM-H15_F	CCATATTACAACAATATTCCTATC	JB11.5	TTATGGTAGATATTATAG	heterodimer	-1.75
JB11.5	TTATGGTAGATATTATAG	atp6st rev	GGAATAATTGCTAACCTACACAAC	heterodimer	-0.18
EM-H15_F	CCATATTACAACAATATTCCTATC	Cest2	CATAAATCAATGGAAACAACAACAAG	heterodimer	0
EM-H17_R	GTGAGTGATTCTTGTTAGGGGAAG	JB11.5	TTATGGTAGATATTATAG	heterodimer	0
Cest2	CATAAATCAATGGAAACAACAACAAG	JB11.5	TTATGGTAGATATTATAG	heterodimer	0
Cest2	CATAAATCAATGGAAACAACAACAAG	R/CO1	GAACCTAACGACATAACATAATGA	heterodimer	0
Cest2	CATAAATCAATGGAAACAACAACAAG	atp6st rev	GGAATAATTGCTAACCTACACAAC	heterodimer	0
JB11.5	TTATGGTAGATATTATAG	R/CO1	GAACCTAACGACATAACATAATGA	heterodimer	0
EM-H17_R	GTGAGTGATTCTTGTTAGGGGAAG	R/CO1	GAACCTAACGACATAACATAATGA	heterodimer	0.35
R/CO1	GAACCTAACGACATAACATAATGA	atp6st rev	GGAATAATTGCTAACCTACACAAC	heterodimer	0.35
EM-H15_F	CCATATTACAACAATATTCCTATC	atp6st rev	GGAATAATTGCTAACCTACACAAC	heterodimer	2.1

Results of the dilution series. DNA content of serially diluted sample. The dilution series was measured on the NanoDrop 2000 device with three technical replicates. **Figure S2:** The graphical overview of all dilutions (from undiluted to 1:64). The figure displays the dilution levels depending on the DNA concentration [ng/µl] in Log_2. The red dotted line symbolised the potential detection limit. **Table S5:** The table presents dilution steps with its nucleic acid concentration in ng/µl (mean value).

Figure S2

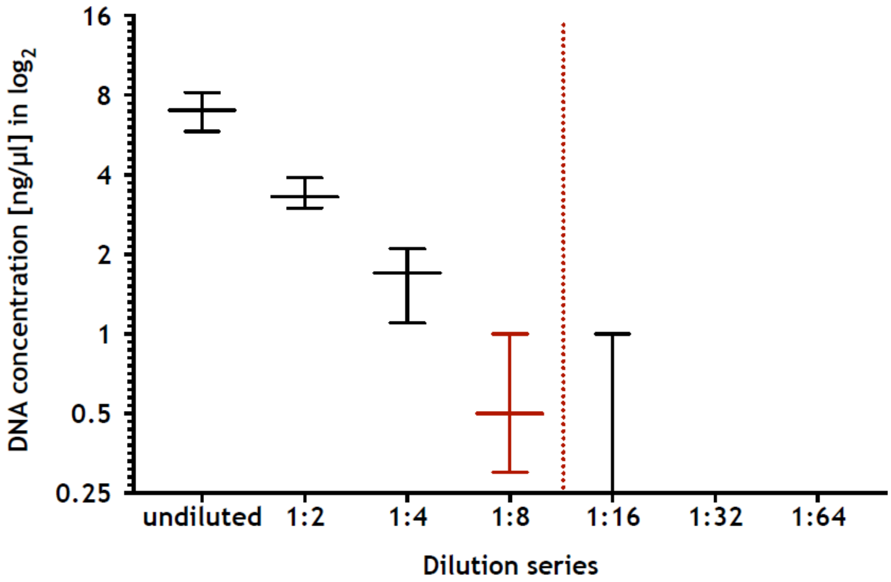

Table S5

„Before RSE“ samples

Dilution	Nucleic acid [ng/µl]
undiluted	7.02
1:2	3.40
1:4	1.63
1:8	0.60
1:16	0.33
1:32	0.00
1:64	0.00

Control qPCR (Isaksson et al., 2014; Maksimov et al., 2019) of the dilution series. The dilution series was measured with three technical replicates. **Figure S3** shows the qPCR results. In the figure, only two of 3 measurements are listed for the undiluted sample, due to a measurement error on the device. The red line symbolised the potential detection limit. The colours of the curves stand for: green = "before RSE", blue = "after RSE", and red = "after REPLI-g" samples. **Table S6:** The table depicted the mean values of the ct values of the dilution steps.

Figure S3

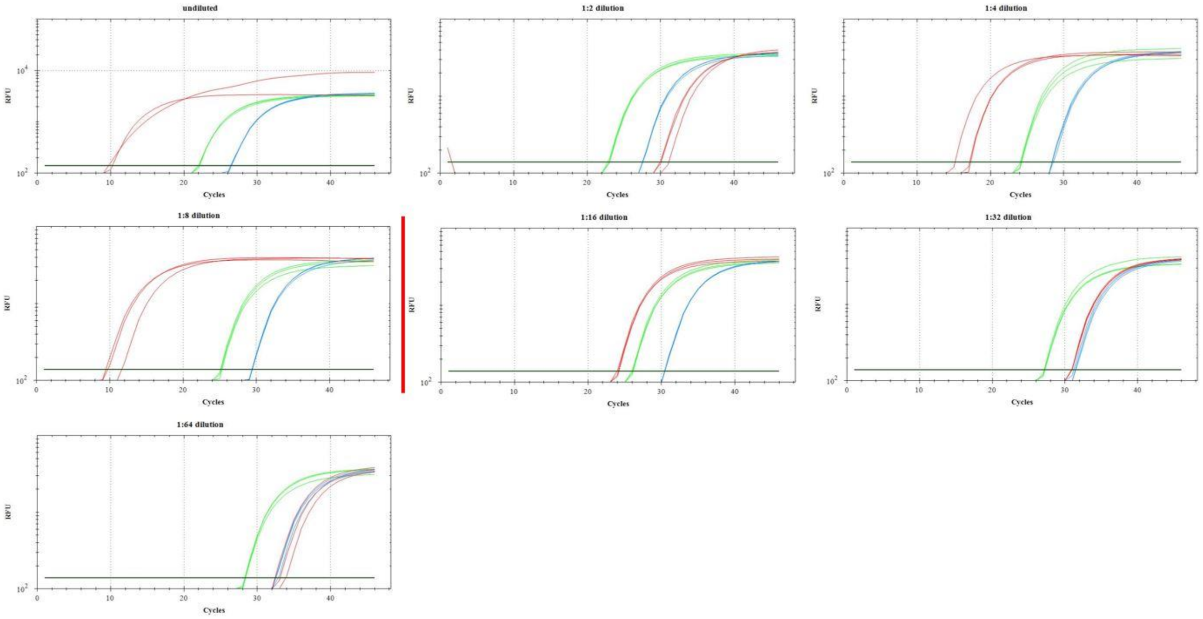

Table S6

Dilution	"Before RSE" sample [ct value]	"After RSE" sample [ct value]	"After REPLI-g" sample [ct value]
undiluted	22.0	26.3	9.9
1:2	23.0	27.6	30.4
1:4	24.1	28.4	16.5
1:8	25.1	29.3	10.3
1:16	26.0	30.4	24.1
1:32	27.1	31.3	31.0
1:64	28.3	32.7	33.1

Figure S4: Result of the significance measurement of the ct values of the qPCR (Isaksson et al., 2014; Maksimov et al., 2019) of all mitogenome samples (Em_1 to Em_14). The graphic shows whisker plots (with min. and max. values) of the "before RSE" (in green), "after RSE" (in blue) and "after REPLI-g" (in red) samples. A one-way ANOVA (created with the GraphPad Prism programme) was carried out for the significance measurement. The p-value is <0.0001 (****) in each case.

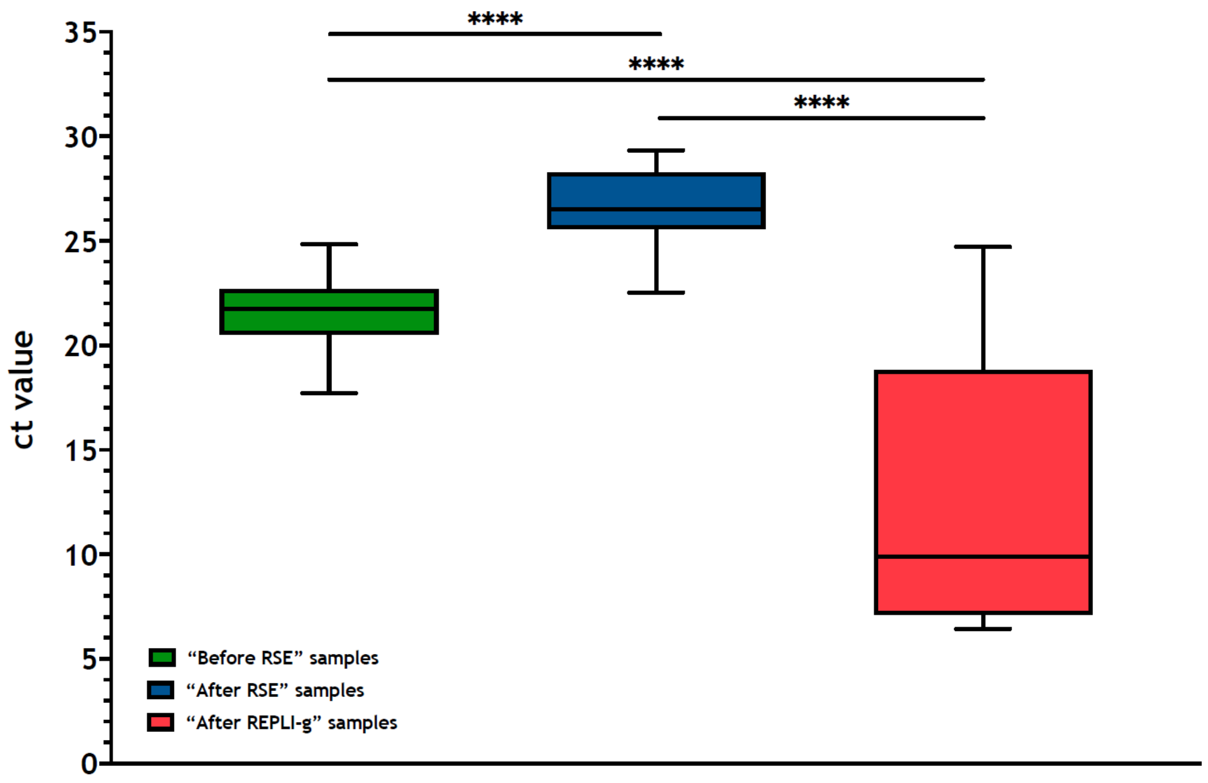

Results of the EmsB microsatellite data. Figure S5: EmsB dendrogram. The name abbreviations stand for: Eg = *Echinococcus granulosus*, Em = *Echinococcus multilocularis*. The samples can be divided into two profiles D and G. An *E. granulosus* sample from Kenya served as an outgroup control (red au number = for approx. unbiased value in percent and green bp number = for bootstrap [B = 1,000]). A genetic threshold of 0.08 was applied for EmsB profile discrimination (red line). **Figure S6:** Electropherograms. Two samples to illustrate the positioning of peaks of *Echinococcus multilocularis* compared to *Echinococcus granulosus*.

Figure S5

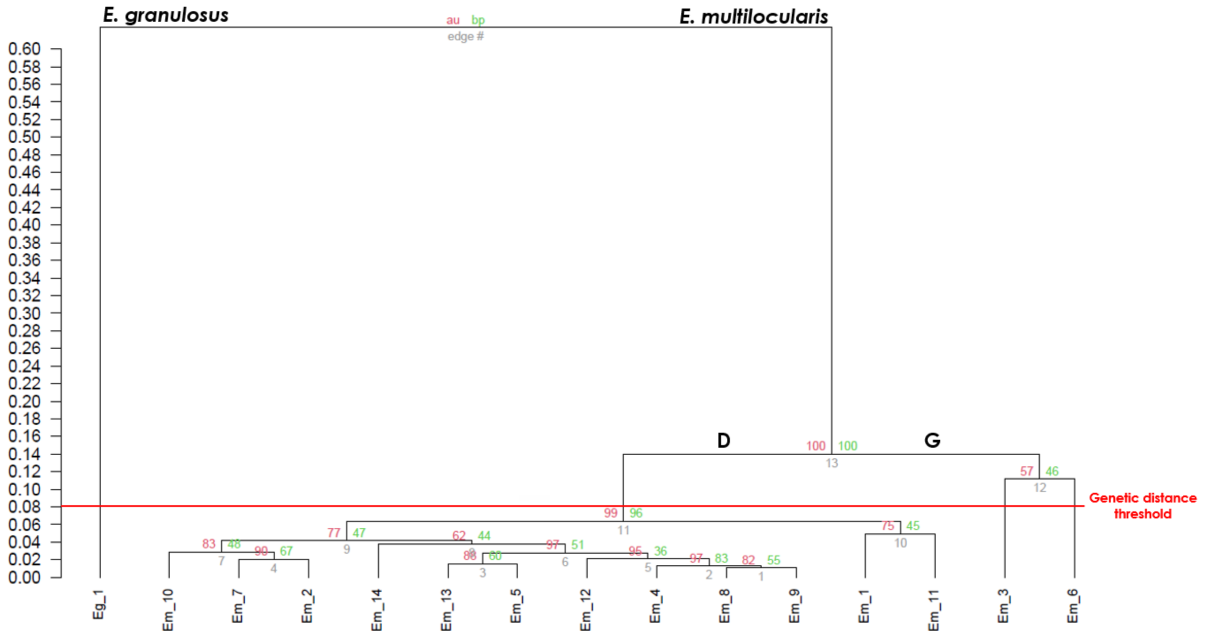

Figure S6

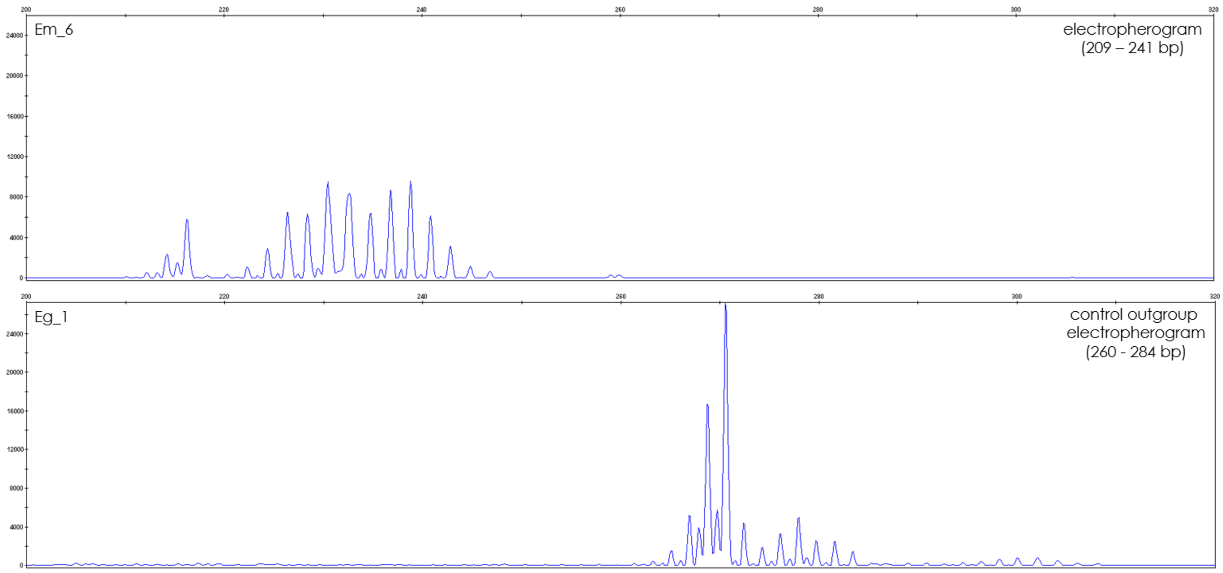

Table S7: Overview of the results for the dilution series. All INDELS and SNPs of the dilution series are depicted relative to the reference genome of *Echinococcus multilocularis* (Nakao et al., 2002). Their positions in the mitogenome, the change at the position, as well as the number of INDELs and SNPs are also shown. A second table summarises only the SNPs.

SNPs & INDELs of the dilution series

	Position [bp]		Dilution step								
**Number**	**Minimum**	**Maximum**	**undiluted**	**1:2**	**1:4**	**1:8**	**1:16**	**1:32**	**1:64**	**Change**	**Polymorphism Type**
1	112	112	A	A	A	A		A		C -> A	Substitution
2	141	141	A	A	A	A		A		G -> A	Substitution
3	148	148	C	C	C	C		C		T -> C	Substitution
4	155	155	T	T	T	T		T		A -> T	Substitution
5	157	157	T	T	T	T		T		A -> T	Substitution
6	363	363	T	T	T	T		T		C -> T	Substitution
7	671	671	A	A	A	A		A		C -> A	Substitution
8	1105	1105	T	T	T	T		T		C -> T	Substitution
9	1325	1325	T	T	T	T	T	T		C -> T	Substitution
10	1502	1502	T	T	T	T		T		C -> T	Substitution
11	1631	1631	A	A	A	A		A		G -> A	Substitution
12	1738	1738	G	G	G	G		G		A -> G	Substitution
13	1774	1774	C	C	C	C		C		T -> C	Substitution
14	1947	1947	G	G	G	G		G		A -> G	Substitution
15	2055	2055	A	A	A	A		A		G -> A	Substitution
16	2144	2144	G	G	G	G		G		A -> G	Substitution
17	2466	2466	T	T	T	T		T		C -> T	Substitution
18	2484	2484	G	G	G	G		G		A -> G	Substitution
19	2658	2658	T	T	T	T		T		C -> T	Substitution
20	3275	3275	C	C	C	C	C	C		T -> C	Substitution
21	3479	3479	C	C	C	C		C		T -> C	Substitution
22	3490	3490	A	A	A	A		A		G -> A	Substitution
23	3798	3798	C	C	C	C		C		T -> C	Substitution
24	4589	4589	G	G	G	G		G		A -> G	Substitution
25	4601	4601	A	A	A	A		A		G -> A	Substitution
26	4671	4671	A	A	A	A		A		G -> A	Substitution
27	4859	4859	C	C	C	C		C		T -> C	Substitution
28	5036	5036	C	C	C	C		C		T -> C	Substitution
29	5132	5132	T	T	T	T		T		C -> T	Substitution
30	5637	5637	C	C	C	C		C		T -> C	Substitution
31	5658	5658	G	G	G	G		G		A -> G	Substitution
32	5835	5835	C	C	C	C		C	C	T -> C	Substitution
33	5911	5911	T	T	T	T		T	T	C -> T	Substitution
34	5924	5924	G	G	G	G		G		T -> G	Substitution
35	6125	6125	A	A	A	A		A		T -> A	Substitution
36	6404	6404	G	G	G	G		G		A -> G	Substitution
37	6430	6430	T	T	T	T		T		G -> T	Substitution
38	6511	6511	C	C	C	C		C		T -> C	Substitution
39	6640	6640	T	T	T	T		T		A -> T	Substitution
40	6726	6726	G	G	G	G		G		T -> G	Substitution
41	6811	6811	C	C	C	C		C		T -> C	Substitution
42	7105	7105	G	G	G	G		G		A -> G	Substitution
43	7451	7451	A	A	A	A		A		G -> A	Substitution
44	7578	7578	A	A	A	A		A		G -> A	Substitution
45	7721	7721	T	T	T	T		T		C -> T	Substitution
46	8717	8717	C	C	C	C		C		T -> C	Substitution
47	8766	8766	C	C	C	C		C		T -> C	Substitution
48	8868	8868	C	C	C	C		C		T -> C	Substitution
49	8916	8916	A	A	A	A		A		G -> A	Substitution
50	9401	9401	C	C	C	C		C		T -> C	Substitution
51	9453	9453	C	C	C	C		C		T -> C	Substitution
52	9839	9839	G	G	G	G		G		T -> G	Substitution
53	9852	9852	T	T	T	T		T		C -> T	Substitution
54	9986	9986	A	A	A	A		A		G -> A	Substitution
55	10493	10493	A	A	A	A		A		G -> A	Substitution
56	10856	10856	A	A	A	A		A		G -> A	Substitution
57	10936	10936	G	G	G	G		G		A -> G	Substitution
58	10944	10944	C	C	C	C		C		T -> C	Substitution
59	10949	10949	A	A	A	A		A		G -> A	Substitution
60	11202	11202	A	A	A	A				G -> A	Substitution
61	11523	11523	-	-	-	-		-		-T	Deletion
62	11992	11992	C	C	C	C		C		T -> C	Substitution
63	11998	11998	G	G	G	G		G		A -> G	Substitution
64	12093	12093	A	A	A	A		A		G -> A	Substitution
65	12329	12329	G	G	G	G		G		A -> G	Substitution
66	12823	12823	G	G	G	G				A -> G	Substitution
67	13188	13188	G	G	G	G				A -> G	Substitution
68	13549	13549	C	C	C	C		C		T -> C	Substitution
	**Number of**										
		**INDELs:**	**1**	**1**	**1**	**1**		**1**			
		**SNPs:**	**67**	**67**	**67**	**67**	**2**	**64**	**2**		

**Legend:**	**-**	**Deletion present**

SNPs of the dilution series

Number	Position [bp]	Dilution step							Change	Polymorphism Type
		undiluted	1:2	1:4	1:8	1:16	1:32	1:64		
1	112	A	A	A	A		A		C -> A	Substitution
2	141	A	A	A	A		A		G -> A	Substitution
3	148	C	C	C	C		C		T -> C	Substitution
4	155	T	T	T	T		T		A -> T	Substitution
5	157	T	T	T	T		T		A -> T	Substitution
6	363	T	T	T	T		T		C -> T	Substitution
7	671	A	A	A	A		A		C -> A	Substitution
8	1105	T	T	T	T		T		C -> T	Substitution
9	1325	T	T	T	T	T	T		C -> T	Substitution
10	1502	T	T	T	T		T		C -> T	Substitution
11	1631	A	A	A	A		A		G -> A	Substitution
12	1738	G	G	G	G		G		A -> G	Substitution
13	1774	C	C	C	C		C		T -> C	Substitution
14	1947	G	G	G	G		G		A -> G	Substitution
15	2055	A	A	A	A		A		G -> A	Substitution
16	2144	G	G	G	G		G		A -> G	Substitution
17	2466	T	T	T	T		T		C -> T	Substitution
18	2484	G	G	G	G		G		A -> G	Substitution
19	2658	T	T	T	T		T		C -> T	Substitution
20	3275	C	C	C	C	C	C		T -> C	Substitution
21	3479	C	C	C	C		C		T -> C	Substitution
22	3490	A	A	A	A		A		G -> A	Substitution
23	3798	C	C	C	C		C		T -> C	Substitution
24	4589	G	G	G	G		G		A -> G	Substitution
25	4601	A	A	A	A		A		G -> A	Substitution
26	4671	A	A	A	A		A		G -> A	Substitution
27	4859	C	C	C	C		C		T -> C	Substitution
28	5036	C	C	C	C		C		T -> C	Substitution
29	5132	T	T	T	T		T		C -> T	Substitution
30	5637	C	C	C	C		C		T -> C	Substitution
31	5658	G	G	G	G		G		A -> G	Substitution
32	5835	C	C	C	C		C	C	T -> C	Substitution
33	5911	T	T	T	T		T	T	C -> T	Substitution
34	5924	G	G	G	G		G		T -> G	Substitution
35	6125	A	A	A	A		A		T -> A	Substitution
36	6404	G	G	G	G		G		A -> G	Substitution
37	6430	T	T	T	T		T		G -> T	Substitution
38	6511	C	C	C	C		C		T -> C	Substitution
39	6640	T	T	T	T		T		A -> T	Substitution
40	6726	G	G	G	G		G		T -> G	Substitution
41	6811	C	C	C	C		C		T -> C	Substitution
42	7105	G	G	G	G		G		A -> G	Substitution
43	7451	A	A	A	A		A		G -> A	Substitution
44	7578	A	A	A	A		A		G -> A	Substitution
45	7721	T	T	T	T		T		C -> T	Substitution
46	8717	C	C	C	C		C		T -> C	Substitution
47	8766	C	C	C	C		C		T -> C	Substitution
48	8868	C	C	C	C		C		T -> C	Substitution
49	8916	A	A	A	A		A		G -> A	Substitution
50	9401	C	C	C	C		C		T -> C	Substitution
51	9453	C	C	C	C		C		T -> C	Substitution
52	9839	G	G	G	G		G		T -> G	Substitution
53	9852	T	T	T	T		T		C -> T	Substitution
54	9986	A	A	A	A		A		G -> A	Substitution
55	10493	A	A	A	A		A		G -> A	Substitution
56	10856	A	A	A	A		A		G -> A	Substitution
57	10936	G	G	G	G		G		A -> G	Substitution
58	10944	C	C	C	C		C		T -> C	Substitution
59	10949	A	A	A	A		A		G -> A	Substitution
60	11202	A	A	A	A				G -> A	Substitution
61	11992	C	C	C	C		C		T -> C	Substitution
62	11998	G	G	G	G		G		A -> G	Substitution
63	12093	A	A	A	A		A		G -> A	Substitution
64	12329	G	G	G	G		G		A -> G	Substitution
65	12823	G	G	G	G				A -> G	Substitution
66	13188	G	G	G	G				A -> G	Substitution
67	13549	C	C	C	C		C		T -> C	Substitution
**Number of**	**SNPs:**	**67**	**67**	**67**	**67**	**2**	**64**	**2**		

Table S8: Overview of the results for the samples. All INDELs and SNPs of all samples in the study are depicted relative to the reference genome of *Echinococcus multilocularis* (Nakao et al., 2002). Their positions in the mitogenome, the change at the position, as well as the number of INDELs and SNPs are also shown. The genes on the mitogenome are marked in different colours. All INDELs and SNPs that occurred in all samples are highlighted in grey.

SNPs & INDELs of the samples

	Position [bp]		Designation & Federal state of Germany															
Gene	Minimum	Maximum	Em_1 (LS)	Em_2 (T)	Em_3 (B)	Em_4 (T)	Em_5 (B)	Em_6 (LS)	Em_7 (T)	Em_8 (T)	Em_9 (T)	Em_10 (T)	Em_11 (T)	Em_12 (B)	Em_13 (T)	Em_14 (T)	Change	Polymorphism Type
	16	16	G					G									A -> G	Substitution
	112	112	A	A	A	A	A	A	A	A	A	A	A	A	A	A	C -> A	Substitution
	132	132				T	T			T	T	T	T	T	T		A -> T	Substitution
	134	134				T	T			T	T	T	T	T	T		A -> T	Substitution
	141	141		A		A	A			A	A	A	A	A	A	A	G -> A	Substitution
	148	148		C		C	C			C	C	C		C	C	C	T -> C	Substitution
	155	155	T	T				T				T				T	A -> T	Substitution
	157	157	T	T				T				T				T	A -> T	Substitution
	270	269			+	+	+			+	+	+	+	+	+		+A	Insertion
	363	363	T	T	T	T	T	T	T	T	T	T	T	T	T	T	C -> T	Substitution
	658	658			A	A	A			A	A	A	A	A	A		G -> A	Substitution
	671	671	A	A	A	A	A	A	A	A	A	A	A	A	A	A	C -> A	Substitution
	1105	1105	T	T	T	T	T	T	T	T	T	T	T	T	T	T	C -> T	Substitution
	1120	1120				A											G -> A	Substitution
	1325	1325	T	T	T	T	T	T	T	T	T	T	T	T	T	T	C -> T	Substitution
	1502	1502	T	T	T	T	T	T	T	T	T	T	T	T	T	T	C -> T	Substitution
nd5	1631	1631	A	A	A	A	A	A	A	A	A	A	A	A	A	A	G -> A	Substitution
	1738	1738	G	G	G	G	G	G	G	G	G	G	G	G	G	G	A -> G	Substitution
	1774	1774	C	C	C	C	C	C	C	C	C	C	C	C	C	C	T -> C	Substitution
	1947	1947	G	G	G	G	G	G	G	G	G	G	G	G	G	G	A -> G	Substitution
	2055	2055	A	A	A	A	A	A	A	A	A	A	A	A	A	A	G -> A	Substitution
	2144	2144	G	G	G	G	G	G	G	G	G	G	G	G	G	G	A -> G	Substitution
	2314	2313							+								+AT	Insertion
	2440	2440							A								G -> A	Substitution
	2466	2466	T	T	T	T	T	T	T	T	T	T	T	T	T	T	C -> T	Substitution
cox3	2471	2471							G								T -> G	Substitution
	2484	2484	G	G	G	G	G	G	G	G	G	G	G	G	G	G	A -> G	Substitution
	2658	2658	T	T				T								T	C -> T	Substitution
	3191	3191			G	G	G			G	G	G	G	G	G		A -> G	Substitution
	3275	3275	C	C	C	C	C	C	C	C	C	C	C	C	C	C	T -> C	Substitution
cyt b	3479	3479	C	C	C	C	C	C	C	C	C	C	C	C	C	C	T -> C	Substitution
	3490	3490	A	A	A	A	A	A	A	A	A	A	A	A	A	A	G -> A	Substitution
	3798	3798	C	C	C	C	C	C	C	C	C	C	C	C	C	C	T -> C	Substitution
	4172	4172							G								A -> G	Substitution
	4589	4589	G	G	G	G	G	G	G	G	G	G	G	G	G	G	A -> G	Substitution
	4601	4601	A	A	A	A	A	A	A	A	A	A	A	A	A	A	G -> A	Substitution
	4671	4671	A	A	A	A	A	A	A	A	A	A	A	A	A	A	G -> A	Substitution
	4859	4859	C	C	C	C	C	C	C	C	C	C	C	C	C	C	T -> C	Substitution
	5036	5036	C	C	C	C	C	C	C	C	C	C	C	C	C	C	T -> C	Substitution
	5132	5132	T	T	T	T	T	T	T	T	T	T	T	T	T	T	C -> T	Substitution
nd 4	5183	5183			A												G -> A	Substitution
	5200	5200	T														C -> T	Substitution
	5294	5294							G								A -> G	Substitution
	5507	5507							A								G -> A	Substitution
	5637	5637	C	C				C	C							C	T -> C	Substitution
	5658	5658		G												G	A -> G	Substitution
	5835	5835	C	C	C	C	C	C	C	C	C	C	C	C	C	C	T -> C	Substitution
	5911	5911	T	T	T	T	T	T	T	T	T	T	T	T	T	T	C -> T	Substitution
	5924	5924	G	G	G	G	G	G	G	G	G	G	G	G	G	G	T -> G	Substitution
atp 6	6055	6055							C								T -> C	Substitution
	6125	6125	A	A	A	A	A	A	A	A	A	A	A	A	A	A	T -> A	Substitution
	6150	6150							A								G -> A	Substitution
	6247	6247				T				T	T						C -> T	Substitution
	6404	6404	G	G	G	G	G	G	G	G	G	G	G	G	G	G	A -> G	Substitution
	6430	6430	T	T	T	T	T	T	T	T	T	T	T	T	T	T	G -> T	Substitution
	6460	6460										A					G -> A	Substitution
nd 2	6511	6511	C	C	C	C	C	C	C	C	C	C	C	C	C	C	T -> C	Substitution
	6640	6640	T	T	T	T	T	T	T	T	T	T	T	T	T	T	A -> T	Substitution
	6726	6726	G	G	G	G	G	G	G	G	G	G	G	G	G	G	T -> G	Substitution
	6811	6811	C	C	C	C	C	C	C	C	C	C	C	C	C	C	T -> C	Substitution
	7105	7105	G	G	G	G	G	G	G	G	G	G	G	G	G	G	A -> G	Substitution
	7277	7277	A					A									T -> A	Substitution
	7349	7349			C	C	C			C	C	C	C	C	C		T -> C	Substitution
	7451	7451	A	A	A	A	A	A	A	A	A	A	A	A	A	A	G -> A	Substitution
	7578	7578	A	A	A	A	A	A	A	A	A	A	A	A	A	A	G -> A	Substitution
	7721	7721	T	T	T	T	T	T	T	T	T	T	T	T	T	T	C -> T	Substitution
nd 1	7725	7725							G								T -> G	Substitution
	7848	7848							C								T -> C	Substitution
	8156	8156				A											G -> A	Substitution
	8708	8708										-					-T	Deletion
	8717	8717	C	C	C	C	C	C	C	C	C	C	C	C	C	C	T -> C	Substitution
nd 3	8766	8766	C	C	C	C	C	C	C	C	C	C	C	C	C	C	T -> C	Substitution
	8868	8868	C	C	C	C	C	C	C	C	C	C	C	C	C	C	T -> C	Substitution
	8916	8916	A	A	A	A	A	A	A	A	A	A	A	A	A	A	G -> A	Substitution
	9163	9163							G								A -> G	Substitution
	9183	9183							T								G -> T	Substitution
	9401	9401	C	C	C	C	C	C	C	C	C	C	C	C	C	C	T -> C	Substitution
	9453	9453	C	C	C	C	C	C	C	C	C	C	C	C	C	C	T -> C	Substitution
	9528	9528										T					G -> T	Substitution
cox 1	9532	9532							T								C -> T	Substitution
	9839	9839	G	G	G	G	G	G	G	G	G	G	G	G	G	G	T -> G	Substitution
	9852	9852	T	T	T	T	T	T	T	T	T	T	T	T	T	T	C -> T	Substitution
	9986	9986	A	A	A	A	A	A	A	A	A	A	A	A	A	A	G -> A	Substitution
	10078	10079	-														-TT	Deletion
	10493	10493	A	A	A	A	A	A	A	A	A	A	A	A	A	A	G -> A	Substitution
	10856	10856	A	A	A	A	A	A	A	A	A	A	A	A	A	A	G -> A	Substitution
	10936	10936	G	G	G	G	G	G	G	G	G	G	G	G	G	G	A -> G	Substitution
	10944	10944	C	C	C	C	C	C	C	C	C	C	C	C	C	C	T -> C	Substitution
	10949	10949	A	A	A	A	A	A	A	A	A	A	A	A	A	A	G -> A	Substitution
	10967	10967	A					A									G -> A	Substitution
	11202	11202	A	A	A	A	A	A	A	A	A	A	A	A	A	A	G -> A	Substitution
	11523	11523	-	-	-	-	-	-	-	-	-	-	-	-	-	-	-T	Deletion
	11561	11561											T				C -> T	Substitution
	11939	11939							G								A -> G	Substitution
	11992	11992		C												C	T -> C	Substitution
	11998	11998	G	G				G								G	A -> G	Substitution
	12018	12018			T	T	T			T	T	T	T	T	T		C -> T	Substitution
	12093	12093	A	A	A	A	A	A	A	A	A	A	A	A	A	A	G -> A	Substitution
	12329	12329	G	G	G	G	G	G	G	G	G	G	G	G	G	G	A -> G	Substitution
cox 2	12823	12823	G	G	G	G	G	G	G	G	G	G	G	G	G	G	A -> G	Substitution
	13188	13188	G	G	G	G	G	G	G	G	G	G	G	G	G	G	A -> G	Substitution
nd 6	13549	13549	C	C	C	C	C	C	C	C	C	C	C	C	C	C	T -> C	Substitution
	**Number of**																	
		**INDELs:**	**2**	**1**	**2**	**2**	**2**	**1**	**2**	**2**	**2**	**3**	**2**	**2**	**2**	**1**		
		**SNPs:**	**67**	**67**	**63**	**69**	**66**	**66**	**72**	**67**	**67**	**70**	**66**	**66**	**66**	**67**		

**Legend:**	+	**Insertion present**
	-	**Deletion present**
	(LS)	**Lower Saxony**
	(T)	**Thuringia**
	(B)	**Brandenburg**

Table S9: SNPs and INDELs of samples sorted after host animal (Red Fox 1 to 4). All SNPs and INDELs are relative to the reference genome of *Echinococcus multilocularis* (Nakao et al., 2002). A second table shows only the SNPs separately. The genes on the mitogenome are marked in different colours. Technical replicates are highlighted in light yellow, while INDELs and SNPs are labelled in red and indicate differences between samples from the same host animal.

Red Fox 1 (SNPs & INDELs of the samples)

	Position [bp]		Designation & Federal state of Germany				
**Gene**	**Minimum**	**Maximum**	**Em_4 (T)**	**Em_8 (T)**	**Em_9 (T)**	**Change**	**Polymorphism Type**
	112	112	A	A	A	C -> A	Substitution
	132	132	T	T	T	A -> T	Substitution
	134	134	T	T	T	A -> T	Substitution
	141	141	A	A	A	G -> A	Substitution
	148	148	C	C	C	T -> C	Substitution
	269	270	+	+	+	+A	Insertion
	363	363	T	T	T	C -> T	Substitution
nd5	658	658	A	A	A	G -> A	Substitution
	671	671	A	A	A	C -> A	Substitution
	1105	1105	T	T	T	C -> T	Substitution
	1120	1120	A			G -> A	Substitution
	1325	1325	T	T	T	C -> T	Substitution
	1502	1502	T	T	T	C -> T	Substitution
	1631	1631	A	A	A	G -> A	Substitution
	1738	1738	G	G	G	A -> G	Substitution
	1774	1774	C	C	C	T -> C	Substitution
	1947	1947	G	G	G	A -> G	Substitution
	2055	2055	A	A	A	G -> A	Substitution
	2144	2144	G	G	G	A -> G	Substitution
cox 3	2466	2466	T	T	T	C -> T	Substitution
	2484	2484	G	G	G	A -> G	Substitution
cyt b	3191	3191	G	G	G	A -> G	Substitution
	3275	3275	C	C	C	T -> C	Substitution
	3479	3479	C	C	C	T -> C	Substitution
	3490	3490	A	A	A	G -> A	Substitution
	3798	3798	C	C	C	T -> C	Substitution
nd 4	4589	4589	G	G	G	A -> G	Substitution
	4601	4601	A	A	A	G -> A	Substitution
	4671	4671	A	A	A	G -> A	Substitution
	4859	4859	C	C	C	T -> C	Substitution
	5036	5036	C	C	C	T -> C	Substitution
	5132	5132	T	T	T	C -> T	Substitution
	5835	5835	C	C	C	T -> C	Substitution
atp 6	5911	5911	T	T	T	C -> T	Substitution
	5924	5924	G	G	G	T -> G	Substitution
	6125	6125	A	A	A	T -> A	Substitution
	6247	6247	T	T	T	C -> T	Substitution
nd 2	6404	6404	G	G	G	A -> G	Substitution
	6430	6430	T	T	T	G -> T	Substitution
	6511	6511	C	C	C	T -> C	Substitution
	6640	6640	T	T	T	A -> T	Substitution
	6726	6726	G	G	G	T -> G	Substitution
	6811	6811	C	C	C	T -> C	Substitution
	7105	7105	G	G	G	A -> G	Substitution
	7349	7349	C	C	C	T -> C	Substitution
	7451	7451	A	A	A	G -> A	Substitution
nd 1	7578	7578	A	A	A	G -> A	Substitution
	7721	7721	T	T	T	C -> T	Substitution
	8156	8156	A			G -> A	Substitution
nd 3	8717	8717	C	C	C	T -> C	Substitution
	8766	8766	C	C	C	T -> C	Substitution
	8868	8868	C	C	C	T -> C	Substitution
	8916	8916	A	A	A	G -> A	Substitution
cox 1	9401	9401	C	C	C	T -> C	Substitution
	9453	9453	C	C	C	T -> C	Substitution
	9839	9839	G	G	G	T -> G	Substitution
	9852	9852	T	T	T	C -> T	Substitution
	9986	9986	A	A	A	G -> A	Substitution
	10493	10493	A	A	A	G -> A	Substitution
	10856	10856	A	A	A	G -> A	Substitution
	10936	10936	G	G	G	A -> G	Substitution
	10944	10944	C	C	C	T -> C	Substitution
	10949	10949	A	A	A	G -> A	Substitution
	11202	11202	A	A	A	G -> A	Substitution
	11523	11523	-	-	-	-T	Deletion
	12018	12018	T	T	T	C -> T	Substitution
	12093	12093	A	A	A	G -> A	Substitution
	12329	12329	G	G	G	A -> G	Substitution
cox 2	12823	12823	G	G	G	A -> G	Substitution
	13188	13188	G	G	G	A -> G	Substitution
nd 6	13549	13549	C	C	C	T -> C	Substitution
	**Number of**						
		**INDELs:**	**2**	**2**	**2**		
		**SNPs:**	**69**	**67**	**67**		

Legend:		
	+	**Insertion present**
	-	**Deletion present**
	(T)	**Thuringia**
		**Technical replicas**

Red Fox 1 (SNPs of the samples)

Gene	Position [bp]	Designation & Federal state of Germany			Change	Polymorphism Type
		Em_4 (T)	Em_8 (T)	Em_9 (T)		
	112	A	A	A	C -> A	Substitution
	132	T	T	T	A -> T	Substitution
	134	T	T	T	A -> T	Substitution
	141	A	A	A	G -> A	Substitution
	148	C	C	C	T -> C	Substitution
	363	T	T	T	C -> T	Substitution
	658	A	A	A	G -> A	Substitution
	671	A	A	A	C -> A	Substitution
	1105	T	T	T	C -> T	Substitution
	1120	A			G -> A	Substitution
	1325	T	T	T	C -> T	Substitution
	1502	T	T	T	C -> T	Substitution
nd5	1631	A	A	A	G -> A	Substitution
	1738	G	G	G	A -> G	Substitution
	1774	C	C	C	T -> C	Substitution
	1947	G	G	G	A -> G	Substitution
	2055	A	A	A	G -> A	Substitution
	2144	G	G	G	A -> G	Substitution
cox 3	2466	T	T	T	C -> T	Substitution
	2484	G	G	G	A -> G	Substitution
	3191	G	G	G	A -> G	Substitution
	3275	C	C	C	T -> C	Substitution
cyt b	3479	C	C	C	T -> C	Substitution
	3490	A	A	A	G -> A	Substitution
	3798	C	C	C	T -> C	Substitution
	4589	G	G	G	A -> G	Substitution
	4601	A	A	A	G -> A	Substitution
nd 4	4671	A	A	A	G -> A	Substitution
	4859	C	C	C	T -> C	Substitution
	5036	C	C	C	T -> C	Substitution
	5132	T	T	T	C -> T	Substitution
	5835	C	C	C	T -> C	Substitution
	5911	T	T	T	C -> T	Substitution
atp 6	5924	G	G	G	T -> G	Substitution
	6125	A	A	A	T -> A	Substitution
	6247	T	T	T	C -> T	Substitution
	6404	G	G	G	A -> G	Substitution
	6430	T	T	T	G -> T	Substitution
	6511	C	C	C	T -> C	Substitution
nd 2	6640	T	T	T	A -> T	Substitution
	6726	G	G	G	T -> G	Substitution
	6811	C	C	C	T -> C	Substitution
	7105	G	G	G	A -> G	Substitution
	7349	C	C	C	T -> C	Substitution
	7451	A	A	A	G -> A	Substitution
	7578	A	A	A	G -> A	Substitution
nd 1	7721	T	T	T	C -> T	Substitution
	8156	A			G -> A	Substitution
	8717	C	C	C	T -> C	Substitution
nd 3	8766	C	C	C	T -> C	Substitution
	8868	C	C	C	T -> C	Substitution
	8916	A	A	A	G -> A	Substitution
	9401	C	C	C	T -> C	Substitution
	9453	C	C	C	T -> C	Substitution
cox 1	9839	G	G	G	T -> G	Substitution
	9852	T	T	T	C -> T	Substitution
	9986	A	A	A	G -> A	Substitution
	10493	A	A	A	G -> A	Substitution
	10856	A	A	A	G -> A	Substitution
	10936	G	G	G	A -> G	Substitution
	10944	C	C	C	T -> C	Substitution
	10949	A	A	A	G -> A	Substitution
	11202	A	A	A	G -> A	Substitution
	11523	-	-	-	-T	Deletion
	12018	T	T	T	C -> T	Substitution
	12093	A	A	A	G -> A	Substitution
	12329	G	G	G	A -> G	Substitution
cox 2	12823	G	G	G	A -> G	Substitution
	13188	G	G	G	A -> G	Substitution
nd 6	13549	C	C	C	T -> C	Substitution
	**Number of SNPs:**	**69**	**67**	**67**		

**Legend:**	(T)	**Thuringia**
		**Technical replicas**

Red Fox 2 (SNPs & INDELs of the samples)

Gene	Position [bp] Minimum	Position [bp] Maximum	Designation & Federal state of Germany Em_2 (T)	Designation & Federal state of Germany Em_14 (T)	Change	Polymorphism Type
	112	112	A	A	C -> A	Substitution
	141	141	A	A	G -> A	Substitution
	148	148	C	C	T -> C	Substitution
	155	155	T	T	A -> T	Substitution
	157	157	T	T	A -> T	Substitution
	363	363	T	T	C -> T	Substitution
	671	671	A	A	C -> A	Substitution
	1105	1105	T	T	C -> T	Substitution
	1325	1325	T	T	C -> T	Substitution
	1502	1502	T	T	C -> T	Substitution
nd 5	1631	1631	A	A	G -> A	Substitution
	1738	1738	G	G	A -> G	Substitution
	1774	1774	C	C	T -> C	Substitution
	1947	1947	G	G	A -> G	Substitution
	2055	2055	A	A	G -> A	Substitution
	2144	2144	G	G	A -> G	Substitution
	2466	2466	T	T	C -> T	Substitution
cox 3	2484	2484	G	G	A -> G	Substitution
	2658	2658	T	T	C -> T	Substitution
	3275	3275	C	C	T -> C	Substitution
cyt b	3479	3479	C	C	T -> C	Substitution
	3490	3490	A	A	G -> A	Substitution
	3798	3798	C	C	T -> C	Substitution
	4589	4589	G	G	A -> G	Substitution
	4601	4601	A	A	G -> A	Substitution
	4671	4671	A	A	G -> A	Substitution
nd 4	4859	4859	C	C	T -> C	Substitution
	5036	5036	C	C	T -> C	Substitution
	5132	5132	T	T	C -> T	Substitution
	5637	5637	C	C	T -> C	Substitution
	5658	5658	G	G	A -> G	Substitution
	5835	5835	C	C	T -> C	Substitution
	5911	5911	T	T	C -> T	Substitution
atp 6	5924	5924	G	G	T -> G	Substitution
	6125	6125	A	A	T -> A	Substitution
	6404	6404	G	G	A -> G	Substitution
	6430	6430	T	T	G -> T	Substitution
	6511	6511	C	C	T -> C	Substitution
nd 2	6640	6640	T	T	A -> T	Substitution
	6726	6726	G	G	T -> G	Substitution
	6811	6811	C	C	T -> C	Substitution
	7105	7105	G	G	A -> G	Substitution
	7451	7451	A	A	G -> A	Substitution
nd 1	7578	7578	A	A	G -> A	Substitution
	7721	7721	T	T	C -> T	Substitution
	8717	8717	C	C	T -> C	Substitution
nd 3	8766	8766	C	C	T -> C	Substitution
	8868	8868	C	C	T -> C	Substitution
	8916	8916	A	A	G -> A	Substitution
	9401	9401	C	C	T -> C	Substitution
	9453	9453	C	C	T -> C	Substitution
cox 1	9839	9839	G	G	T -> G	Substitution
	9852	9852	T	T	C -> T	Substitution
	9986	9986	A	A	G -> A	Substitution
	10493	10493	A	A	G -> A	Substitution
	10856	10856	A	A	G -> A	Substitution
	10936	10936	G	G	A -> G	Substitution
	10944	10944	C	C	T -> C	Substitution
	10949	10949	A	A	G -> A	Substitution
	11202	11202	A	A	G -> A	Substitution
	11523	11523	-	-	-T	Deletion
	11992	11992	C	C	T -> C	Substitution
	11998	11998	G	G	A -> G	Substitution
	12093	12093	A	A	G -> A	Substitution
	12329	12329	G	G	A -> G	Substitution
cox 2	12823	12823	G	G	A -> G	Substitution
	13188	13188	G	G	A -> G	Substitution
nd 6	13549	13549	C	C	T -> C	Substitution
	Number of					
		**INDELs:**	**1**	**1**		
		**SNPs:**	**67**	**67**		

Legend:		
	+	Insertion present
	-	Deletion present
	(T)	Thuringia

Red Fox 2 (SNPs of the samples)

Legend: (T) Thuringia

Gene	Position [bp]	Designation & Federal state of Germany: Em_2 (T)	Em_14 (T)	Change	Polymorphism Type
	112	A	A	C -> A	Substitution
	141	A	A	G -> A	Substitution
	148	C	C	T -> C	Substitution
	155	T	T	A -> T	Substitution
	157	T	T	A -> T	Substitution
	363	T	T	C -> T	Substitution
	671	A	A	C -> A	Substitution
	1105	T	T	C -> T	Substitution
	1325	T	T	C -> T	Substitution
	1502	T	T	C -> T	Substitution
nd 5	1631	A	A	G -> A	Substitution
	1738	G	G	A -> G	Substitution
	1774	C	C	T -> C	Substitution
	1947	G	G	A -> G	Substitution
	2055	A	A	G -> A	Substitution
	2144	G	G	A -> G	Substitution
	2466	T	T	C -> T	Substitution
cox 3	2484	G	G	A -> G	Substitution
	2658	T	T	C -> T	Substitution
	3275	C	C	T -> C	Substitution
cyt b	3479	C	C	T -> C	Substitution
	3490	A	A	G -> A	Substitution
	3798	C	C	T -> C	Substitution
	4589	G	G	A -> G	Substitution
	4601	A	A	G -> A	Substitution
	4671	A	A	G -> A	Substitution
	4859	C	C	T -> C	Substitution
nd 4	5036	C	C	T -> C	Substitution
	5132	T	T	C -> T	Substitution
	5637	C	C	T -> C	Substitution
	5658	G	G	A -> G	Substitution
	5835	C	C	T -> C	Substitution
	5911	T	T	C -> T	Substitution
atp 6	5924	G	G	T -> G	Substitution
	6125	A	A	T -> A	Substitution
	6404	G	G	A -> G	Substitution
	6430	T	T	G -> T	Substitution
	6511	C	C	T -> C	Substitution
nd 2	6640	T	T	A -> T	Substitution
	6726	G	G	T -> G	Substitution
	6811	C	C	T -> C	Substitution
	7105	G	G	A -> G	Substitution
	7451	A	A	G -> A	Substitution
nd 1	7578	A	A	G -> A	Substitution
	7721	T	T	C -> T	Substitution
	8717	C	C	T -> C	Substitution
nd 3	8766	C	C	T -> C	Substitution
	8868	C	C	T -> C	Substitution
	8916	A	A	G -> A	Substitution
	9401	C	C	T -> C	Substitution
	9453	C	C	T -> C	Substitution
cox 1	9839	G	G	T -> G	Substitution
	9852	T	T	C -> T	Substitution
	9986	A	A	G -> A	Substitution
	10493	A	A	G -> A	Substitution
	10856	A	A	G -> A	Substitution
	10936	G	G	A -> G	Substitution
	10944	C	C	T -> C	Substitution
	10949	A	A	G -> A	Substitution
	11202	A	A	G -> A	Substitution
	11992	C	C	T -> C	Substitution
	11998	G	G	A -> G	Substitution
	12093	A	A	G -> A	Substitution
	12329	G	G	A -> G	Substitution
cox 2	12823	G	G	A -> G	Substitution
	13188	G	G	A -> G	Substitution
nd 6	13549	C	C	T -> C	Substitution
	**Number of SNPs:**	**67**	**67**		

Supplement 2

Red Fox 3 (SNPs & INDELs of the samples)

	Position [bp]		Designation & Federal state of Germany			
**Gene**	**Minimum**	**Maximum**	**Em_1 (LS)**	**Em_6 (LS)**	**Change**	**Polymorphism Type**
	16	16	G	G	A -> G	Substitution
	112	112	A	A	C -> A	Substitution
	155	155	T	T	A -> T	Substitution
	157	157	T	T	A -> T	Substitution
	363	363	T	T	C -> T	Substitution
	671	671	A	A	C -> A	Substitution
	1105	1105	T	T	C -> T	Substitution
	1325	1325	T	T	C -> T	Substitution
	1502	1502	T	T	C -> T	Substitution
nd 5	1631	1631	A	A	G -> A	Substitution
	1738	1738	G	G	A -> G	Substitution
	1774	1774	C	C	T -> C	Substitution
	1947	1947	G	G	A -> G	Substitution
	2055	2055	A	A	G -> A	Substitution
	2144	2144	G	G	A -> G	Substitution
	2466	2466	T	T	C -> T	Substitution
cox 3	2484	2484	G	G	A -> G	Substitution
	2658	2658	T	T	C -> T	Substitution
	3275	3275	C	C	T -> C	Substitution
cyt b	3479	3479	C	C	T -> C	Substitution
	3490	3490	A	A	G -> A	Substitution
	3798	3798	C	C	T -> C	Substitution
	4589	4589	G	G	A -> G	Substitution
	4601	4601	A	A	G -> A	Substitution
	4671	4671	A	A	G -> A	Substitution
nd 4	4859	4859	C	C	T -> C	Substitution
	5036	5036	C	C	T -> C	Substitution
	5132	5132	T	T	C -> T	Substitution
	5200	5200	T		C -> T	Substitution
	5637	5637	C	C	T -> C	Substitution
	5835	5835	C	C	T -> C	Substitution
	5911	5911	T	T	C -> T	Substitution
atp 6	5924	5924	G	G	T -> G	Substitution
	6125	6125	A	A	T -> A	Substitution
	6404	6404	G	G	A -> G	Substitution
	6430	6430	T	T	G -> T	Substitution
	6511	6511	C	C	T -> C	Substitution
nd 2	6640	6640	T	T	A -> T	Substitution
	6726	6726	G	G	T -> G	Substitution
	6811	6811	C	C	T -> C	Substitution
	7105	7105	G	G	A -> G	Substitution
	7277	7277	A	A	T -> A	Substitution
	7451	7451	A	A	G -> A	Substitution
nd 1	7578	7578	A	A	G -> A	Substitution
	7721	7721	T	T	C -> T	Substitution
	8717	8717	C	C	T -> C	Substitution
nd 3	8766	8766	C	C	T -> C	Substitution
	8868	8868	C	C	T -> C	Substitution
	8916	8916	A	A	G -> A	Substitution
	9401	9401	C	C	T -> C	Substitution
	9453	9453	C	C	T -> C	Substitution
	9839	9839	G	G	T -> G	Substitution
cox 1	9852	9852	T	T	C -> T	Substitution
	9986	9986	A	A	G -> A	Substitution
	10078	10079	-		-TT	Deletion
	10493	10493	A	A	G -> A	Substitution
	10856	10856	A	A	G -> A	Substitution
	10936	10936	G	G	A -> G	Substitution
	10944	10944	C	C	T -> C	Substitution
	10949	10949	A	A	G -> A	Substitution
	10967	10967	A	A	G -> A	Substitution
	11202	11202	A	A	G -> A	Substitution
	11523	11523	-	-	-T	Deletion
	11998	11998	G	G	A -> G	Substitution
	12093	12093	A	A	G -> A	Substitution
	12329	12329	G	G	A -> G	Substitution
cox 2	12823	12823	G	G	A -> G	Substitution
	13188	13188	G	G	A -> G	Substitution
nd 6	13549	13549	C	C	T -> C	Substitution
	**Number of**					
		**INDELs:**	**2**	**1**		
		**SNPs:**	**67**	**66**		

**Legend:**		
	+	**Insertion present**
	-	**Deletion present**
	(LS)	**Lower Saxony**

Supplement 2

Red Fox 3 (SNPs of the samples)

Gene	Position [bp]	Designation & Federal state of Germany: Em_1 (LS)	Em_6 (LS)	Change	Polymorphism Type
	16	G	G	A -> G	Substitution
	112	A	A	C -> A	Substitution
	155	T	T	A -> T	Substitution
	157	T	T	A -> T	Substitution
	363	T	T	C -> T	Substitution
	671	A	A	C -> A	Substitution
	1105	T	T	C -> T	Substitution
	1325	T	T	C -> T	Substitution
	1502	T	T	C -> T	Substitution
nd 5	1631	A	A	G -> A	Substitution
	1738	G	G	A -> G	Substitution
	1774	C	C	T -> C	Substitution
	1947	G	G	A -> G	Substitution
	2055	A	A	G -> A	Substitution
	2144	G	G	A -> G	Substitution
	2466	T	T	C -> T	Substitution
cox 3	2484	G	G	A -> G	Substitution
	2658	T	T	C -> T	Substitution
	3275	C	C	T -> C	Substitution
cyt b	3479	C	C	T -> C	Substitution
	3490	A	A	G -> A	Substitution
	3798	C	C	T -> C	Substitution
	4589	G	G	A -> G	Substitution
	4601	A	A	G -> A	Substitution
	4671	A	A	G -> A	Substitution
nd 4	4859	C	C	T -> C	Substitution
	5036	C	C	T -> C	Substitution
	5132	T	T	C -> T	Substitution
	5200	T		C -> T	Substitution
	5637	C	C	T -> C	Substitution
	5835	C	C	T -> C	Substitution
	5911	T	T	C -> T	Substitution
atp 6	5924	G	G	T -> G	Substitution
	6125	A	A	T -> A	Substitution
	6404	G	G	A -> G	Substitution
	6430	T	T	G -> T	Substitution
	6511	C	C	T -> C	Substitution
nd 2	6640	T	T	A -> T	Substitution
	6726	G	G	T -> G	Substitution
	6811	C	C	T -> C	Substitution
	7105	G	G	A -> G	Substitution
	7277	A	A	T -> A	Substitution
	7451	A	A	G -> A	Substitution
nd 1	7578	A	A	G -> A	Substitution
	7721	T	T	C -> T	Substitution
	8717	C	C	T -> C	Substitution
nd 3	8766	C	C	T -> C	Substitution
	8868	C	C	T -> C	Substitution
	8916	A	A	G -> A	Substitution
	9401	C	C	T -> C	Substitution
	9453	C	C	T -> C	Substitution
cox 1	9839	G	G	T -> G	Substitution
	9852	T	T	C -> T	Substitution
	9986	A	A	G -> A	Substitution
	10493	A	A	G -> A	Substitution
	10856	A	A	G -> A	Substitution
	10936	G	G	A -> G	Substitution
	10944	C	C	T -> C	Substitution
	10949	A	A	G -> A	Substitution
	10967	A	A	G -> A	Substitution
	11202	A	A	G -> A	Substitution
	11998	G	G	A -> G	Substitution
	12093	A	A	G -> A	Substitution
	12329	G	G	A -> G	Substitution
cox 2	12823	G	G	A -> G	Substitution
	13188	G	G	A -> G	Substitution
nd 6	13549	C	C	T -> C	Substitution
	**Number of SNPs:**	**67**	**66**		

**Legend:**	(LS)	**Lower Saxony**

Red Fox 4 (SNPs & INDELs of the samples)

	Position [bp]		Designation & Federal state of Germany			
**Gene**	**Minimum**	**Maximum**	**Em_5 (B)**	**Em_12 (B)**	**Change**	**Polymorphism Type**
	112	112	A	A	C -> A	Substitution
	132	132	T	T	A -> T	Substitution
	134	134	T	T	A -> T	Substitution
	141	141	A	A	G -> A	Substitution
	148	148	C	C	T -> C	Substitution
	270	269	+	+	+A	Insertion
	363	363	T	T	C -> T	Substitution
	658	658	A	A	G -> A	Substitution
	671	671	A	A	C -> A	Substitution
	1105	1105	T	T	C -> T	Substitution
	1325	1325	T	T	C -> T	Substitution
	1502	1502	T	T	C -> T	Substitution
nd5	1631	1631	A	A	G -> A	Substitution
	1738	1738	G	G	A -> G	Substitution
	1774	1774	C	C	T -> C	Substitution
	1947	1947	G	G	A -> G	Substitution
	2055	2055	A	A	G -> A	Substitution
	2144	2144	G	G	A -> G	Substitution
cox 3	2466	2466	T	T	C -> T	Substitution
	2484	2484	G	G	A -> G	Substitution
	3191	3191	G	G	A -> G	Substitution
	3275	3275	C	C	T -> C	Substitution
cyt b	3479	3479	C	C	T -> C	Substitution
	3490	3490	A	A	G -> A	Substitution
	3798	3798	C	C	T -> C	Substitution
	4589	4589	G	G	A -> G	Substitution
	4601	4601	A	A	G -> A	Substitution
nd 4	4671	4671	A	A	G -> A	Substitution
	4859	4859	C	C	T -> C	Substitution
	5036	5036	C	C	T -> C	Substitution
	5132	5132	T	T	C -> T	Substitution
	5835	5835	C	C	T -> C	Substitution
	5911	5911	T	T	C -> T	Substitution
atp 6	5924	5924	G	G	T -> G	Substitution
	6125	6125	A	A	T -> A	Substitution
	6404	6404	G	G	A -> G	Substitution
	6430	6430	T	T	G -> T	Substitution
	6511	6511	C	C	T -> C	Substitution
nd 2	6640	6640	T	T	A -> T	Substitution
	6726	6726	G	G	T -> G	Substitution
	6811	6811	C	C	T -> C	Substitution
	7105	7105	G	G	A -> G	Substitution
	7349	7349	C	C	T -> C	Substitution
	7451	7451	A	A	G -> A	Substitution
nd 1	7578	7578	A	A	G -> A	Substitution
	7721	7721	T	T	C -> T	Substitution
	8717	8717	C	C	T -> C	Substitution
nd 3	8766	8766	C	C	T -> C	Substitution
	8868	8868	C	C	T -> C	Substitution
	8916	8916	A	A	G -> A	Substitution
	9401	9401	C	C	T -> C	Substitution
	9453	9453	C	C	T -> C	Substitution
cox 1	9839	9839	G	G	T -> G	Substitution
	9852	9852	T	T	C -> T	Substitution
	9986	9986	A	A	G -> A	Substitution
	10493	10493	A	A	G -> A	Substitution
	10856	10856	A	A	G -> A	Substitution
	10936	10936	G	G	A -> G	Substitution
	10944	10944	C	C	T -> C	Substitution
	10949	10949	A	A	G -> A	Substitution
	11202	11202	A	A	G -> A	Substitution
	11523	11523	-	-	-T	Deletion
	12018	12018	T	T	C -> T	Substitution
	12093	12093	A	A	G -> A	Substitution
	12329	12329	G	G	A -> G	Substitution
cox 2	12823	12823	G	G	A -> G	Substitution
	13188	13188	G	G	A -> G	Substitution
nd 6	13549	13549	C	C	T -> C	Substitution
	**Number of**					
		**INDELs:**	**2**	**2**		
		**SNPs:**	**66**	**66**		

**Legend:**		
	+	**Insertion present**
	-	**Deletion present**
	(B)	**Brandenburg**

Red Fox 4 (SNPs of the samples)

Legend:	(B)	Brandenburg

		Designation & Federal state of Germany			
**Gene**	**Position [bp]**	**Em_5 (B)**	**Em_12 (B)**	**Change**	**Polymorphism Type**
	112	A	A	C -> A	Substitution
	132	T	T	A -> T	Substitution
	134	T	T	A -> T	Substitution
	141	A	A	G -> A	Substitution
	148	C	C	T -> C	Substitution
	363	T	T	C -> T	Substitution
	658	A	A	G -> A	Substitution
	671	A	A	C -> A	Substitution
	1105	T	T	C -> T	Substitution
	1325	T	T	C -> T	Substitution
	1502	T	T	C -> T	Substitution
nd 5	1631	A	A	G -> A	Substitution
	1738	G	G	A -> G	Substitution
	1774	C	C	T -> C	Substitution
	1947	G	G	A -> G	Substitution
	2055	A	A	G -> A	Substitution
	2144	G	G	A -> G	Substitution
cox 3	2466	T	T	C -> T	Substitution
	2484	G	G	A -> G	Substitution
	3191	G	G	A -> G	Substitution
	3275	C	C	T -> C	Substitution
cyt b	3479	C	C	T -> C	Substitution
	3490	A	A	G -> A	Substitution
	3798	C	C	T -> C	Substitution
	4589	G	G	A -> G	Substitution
	4601	A	A	G -> A	Substitution
nd 4	4671	A	A	G -> A	Substitution
	4859	C	C	T -> C	Substitution
	5036	C	C	T -> C	Substitution
	5132	T	T	C -> T	Substitution
	5835	C	C	T -> C	Substitution
	5911	T	T	C -> T	Substitution
atp 6	5924	G	G	T -> G	Substitution
	6125	A	A	T -> A	Substitution
	6404	G	G	A -> G	Substitution
	6430	T	T	G -> T	Substitution
	6511	C	C	T -> C	Substitution
nd 2	6640	T	T	A -> T	Substitution
	6726	G	G	T -> G	Substitution
	6811	C	C	T -> C	Substitution
	7105	G	G	A -> G	Substitution
	7349	C	C	T -> C	Substitution
	7451	A	A	G -> A	Substitution
nd 1	7578	A	A	G -> A	Substitution
	7721	T	T	C -> T	Substitution
	8717	C	C	T -> C	Substitution
nd 3	8766	C	C	T -> C	Substitution
	8868	C	C	T -> C	Substitution
	8916	A	A	G -> A	Substitution
	9401	C	C	T -> C	Substitution
	9453	C	C	T -> C	Substitution
cox 1	9839	G	G	T -> G	Substitution
	9852	T	T	C -> T	Substitution
	9986	A	A	G -> A	Substitution
	10493	A	A	G -> A	Substitution
	10856	A	A	G -> A	Substitution
	10936	G	G	A -> G	Substitution
	10944	C	C	T -> C	Substitution
	10949	A	A	G -> A	Substitution
	11202	A	A	G -> A	Substitution
	12018	T	T	C -> T	Substitution
	12093	A	A	G -> A	Substitution
	12329	G	G	A -> G	Substitution
cox 2	12823	G	G	A -> G	Substitution
	13188	G	G	A -> G	Substitution
nd 6	13549	C	C	T -> C	Substitution
	**Number of SNPs:**	**66**	**66**		

Figure S7: Haplotype network analysis of the *Echinococcus multilocularis* mitogenome. (**A)** Haplotype network represented by individuals (samples with all specifications), **(B)** Haplotype network represented by individuals (only with sample ID). (**C)** Haplotype network sorted by host animal (Red Fox). (**D)** Haplotype network sorted by regions (i.e. German Federal States). (**A)** to (**D**) created after Toparslan et al. (2020). The name abbreviations stand for: Em_X = *Echinococcus multilocularis* sample with sample ID (with X = 1 to 14), B = Brandenburg, LS = Lower Saxony, T = Thuringia, Red Fox_X = Host animal with animal ID (with X = 1 to 9), HX = Haplotype number (with X = 1 to 10). The short line in the picture represent one SNP each.

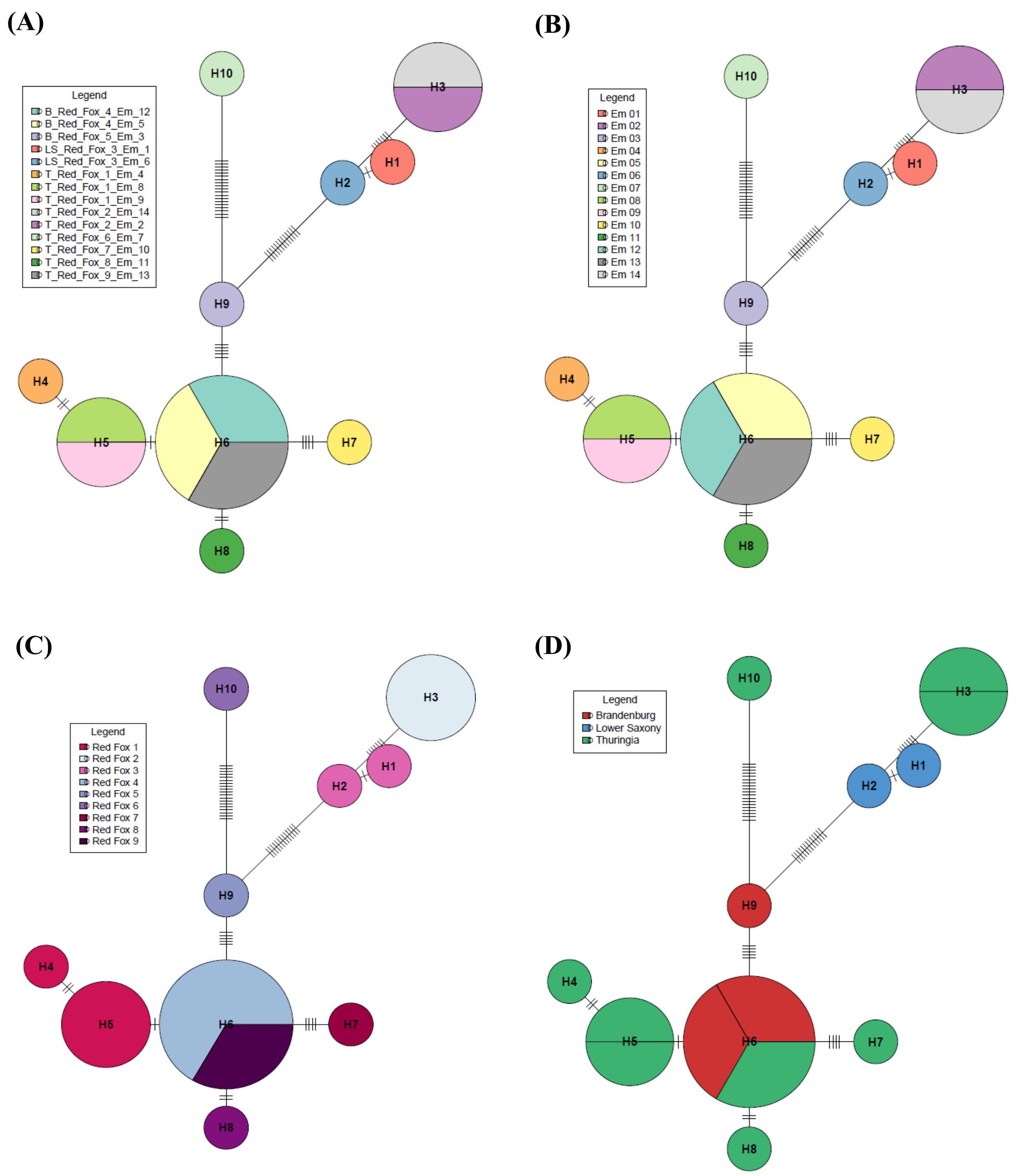

Haplotype network analysis of the *Echinococcus multilocularis* mitogenome. **Figure S8**: Haplotype network sorted by Federal state created with the PopArt program (to check the results). The name abbreviations stand for: Em_X = *Echinococcus multilocularis* sample with sample ID (with X = 1 to 14), B = Brandenburg (red), LS = Lower Saxony (blue), T = Thuringia (green), Red Fox_X = Host animal with animal ID (with X = 1 to 9). The short line in the picture represent one SNP each. **Table S10**: statistic parameters created with the program PopArt. Nucleotide diversity (pi) as the average number of nucleotide differences per site between two DNA sequences in all possible sample pairs in the population (Al-Jawabreh et al., 2023). Segregating sites is the number of sites that differ among sequences. Parsimony-informative site contains at least two types of nucleotides (or amino acids), and at least two of them occur with a minimum frequency of two. Tajima's D is a measure of selection (a neutrality test). **Figure S8** and **Table S10** created after Leigh and Bryant (2015).

Figure S8

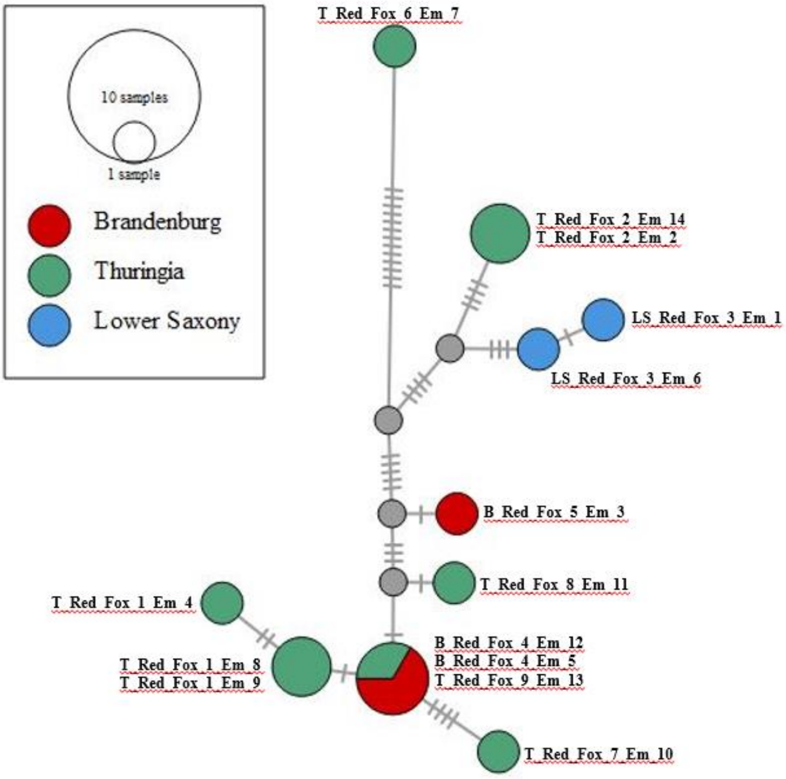

Table S10

Statistic parameter	Results
Nucleotide diversity	pi = 0.000637705
Number of segregating sites	39
Number of parsimony-informative sites	19
Tajima's D statistic	D = -1.24189
	p (D >= -1.24189) = 0.88595

Haplotype network analysis of the *Echinococcus multilocularis* mitogenome. Table S11: Frequence of haplotypes. **Table S12**: Number of haplotypes per region with B = Brandenburg, LS = Lower Saxony, and T = Thuringia. **Table S13**: Haplotype distance matrix (Hamming distance method). **Table S11** to **Table S13** created after Toparslan et al. (2020).

Table S11

Haplotypes	Sequences length (42 base pairs)	Frequences of haplotypes	Percentages of haplotype frequences
H1	G A A G T T T G G G T T A A G T A G C A T G C G A T T T G T A G G C - - A C A T G C	1	7.14
H2	. . . . C . . . . T T	1	7.14
H3	A . . A C . . . . C . . . G . . . . T . . . . T T G . . C . .	2	14.29
H4	A T T A C A A A A . . C G . . C . . T . . . T . T C . . A . . . . . T T G . . . A T	1	7.14
H5	A T T A C A A A . . . C G . . C . . T . . . T . T C . . . . T T G . . . A T	2	14.29
H6	A T T A C A A A . . . C G . . C . . T . . . . . T C . . . . T T G . . . A T	3	21.43
H7	A T T A C . . A . . . C G . . C . . T . . . . A T C . . . - . . T . T T G . . . A T	1	7.14
H8	A T T A . A A A . . . C G . . C . . T . . . . . T C . . . . T T G T . . A T	1	7.14
H9	A . . . . A A A . . . C G . A C . . T . . . . . T C . . . . T T G . . . A T	1	7.14
H10	A . . . . A A . . A G C . G . C G A . . C A . . T . G C . . G T . T T T G . G . A .	1	7.14

Table S12

Haplotypes	B	LS	T
H1	0	1	0
H2	0	1	0
H3	0	0	2
H4	0	0	1
H5	0	0	2
H6	2	0	1
H7	0	0	1
H8	0	0	1
H9	1	0	0
H10	0	0	1

Table S13

	H1	H2	H3	H4	H5	H6	H7	H8	H9	H10
H1	0	3	10	22	20	19	20	19	16	23
H2	3	0	7	19	17	16	17	16	13	20
H3	10	7	0	16	14	13	14	15	14	21
H4	22	19	16	0	2	3	8	5	8	25
H5	20	17	14	2	0	1	6	3	6	23
H6	19	16	13	3	1	0	5	2	5	22
H7	20	17	14	8	6	5	0	7	10	27
H8	19	16	15	5	3	2	7	0	5	22
H9	16	13	14	8	6	5	10	5	0	19
H10	23	20	21	25	23	22	27	22	19	0

Figure S9: Haplotype network analysis of the *Echinococcus multilocularis* mitogenome. (**J**) and (**K**) created after Toparslan et al. (2020). (**J**) Heat map based on the number of nucleotide differences between the haplotypes. Each branch of the phylogenetic tree represents the corresponding haplotype in the matrix. Colours: dark red = close relationships, white = far relationships. (**K**) Neighbor-joining (NJ) tree for mitogenome of *Echinococcus multilocularis* (Hamming distance method of nucleotide differences). Coloured internal nodes represent the bootstrap confidence level (values were specified by colouring according to confidence intervals). Bootstrap Percentage (BP) ≥85 the confidence interval is strong, 70 ≤ BP < 85 moderate, and weak for 50 ≤ BP < 70.

(J)

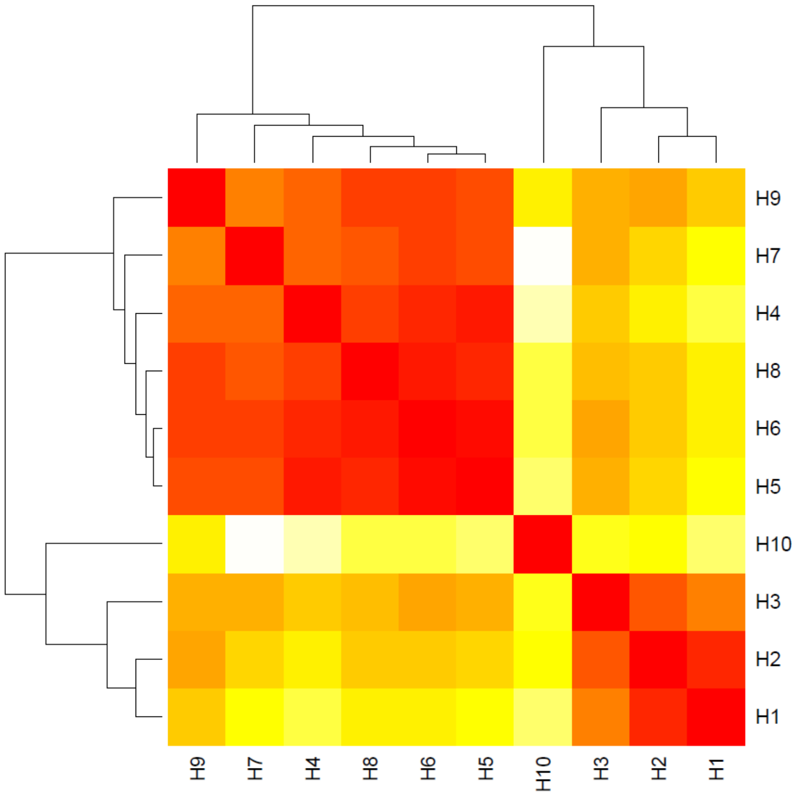

(K)

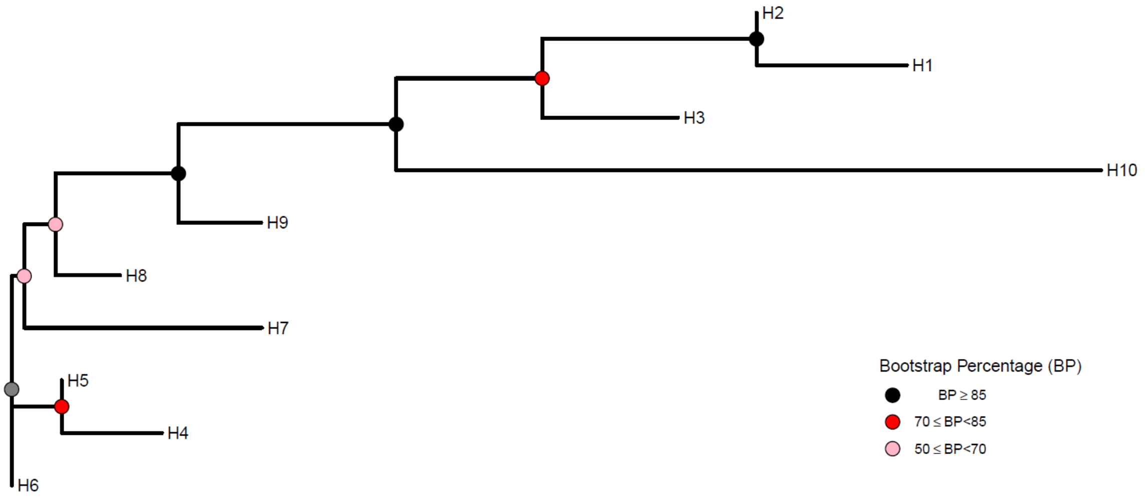

Figure S10: Workflow of phenol-chloroform method. *Created in BioRender. Rachel, F. (2025) https://BioRender.com/z91y885*

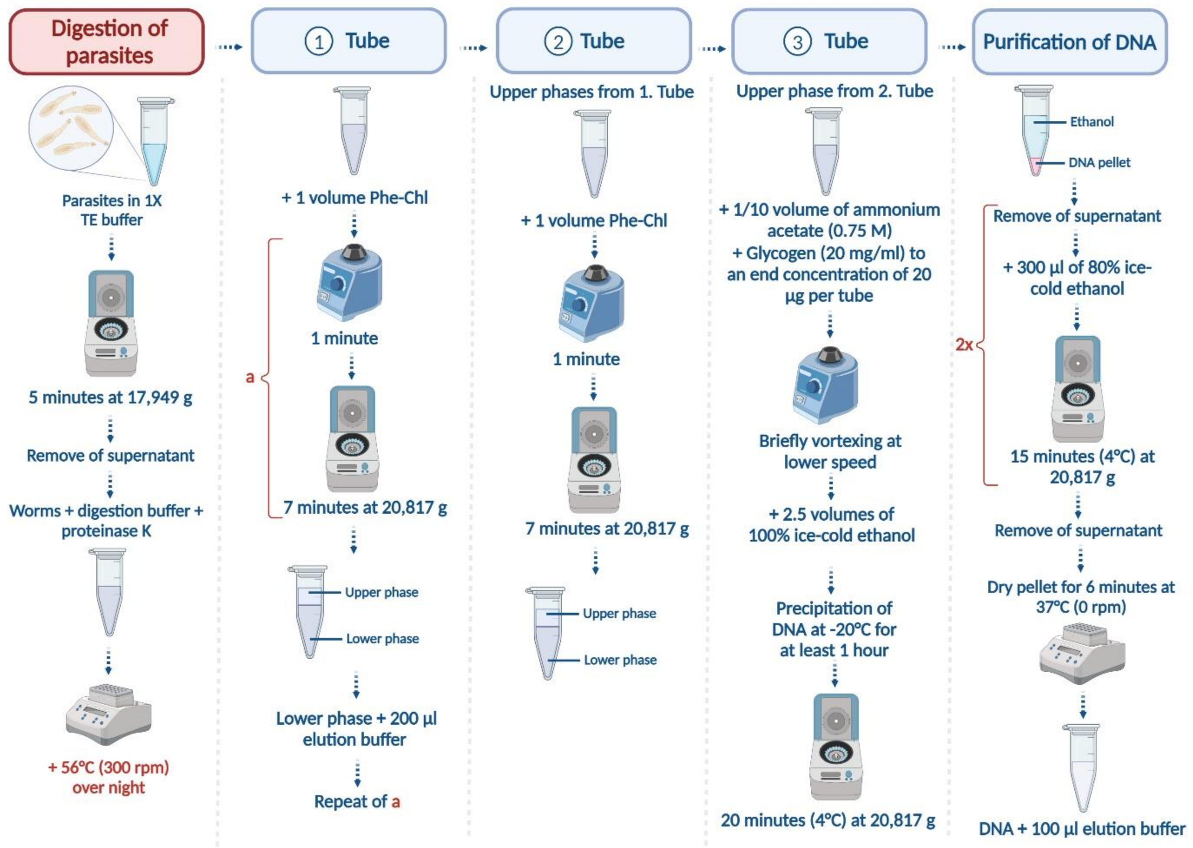

Table S14: NGS data of the WGS samples. Legend: ≥ 30X = proportion of genome with at least 30-fold coverage, ≥ 50X = proportion of genome with at least 50-fold coverage, % Aligned = percentage of mapped reads, Aligned = number of mapped reads, and Total reads = number of reads. Generated by QualiMap v.2.2.2-dev.

Sample ID	≥ 30X	≥ 50X	Mean depth [X]	% Aligned	Aligned	Total reads	mean mapping quality
Em_9_WGS	100.0%	100.0%	1516.6	0.27%	143829.0	54,188,571	59.8
Em_10_WGS	100.0%	100.0%	8198.9	0.71%	773874.0	108,957,541	59.8
Em_11_WGS	100.0%	100.0%	2980.7	0.49%	288678.0	58,851,531	59.9
Em_12_WGS	99,88%	99,64%	492.8	0.10%	52027.0	52,976,892	59.8

REFERENCES

Al-Jawabreh, A., Ereqat, S., Al-Jawabreh, H., Dumaidi, K., and Nasereddin, A. (2023). Genetic diversity and haplotype analysis of Leishmania tropica identified in sand fly vectors of the genera Phlebotomus and Sergentomyia using next-generation sequencing technology. *Parasitol Res* 122, 1351–1360. doi: 10.1007/s00436-023-07835-1

Isaksson, M., Hagström, Å., Armua-Fernandez, M. T., Wahlström, H., Ågren, E. O., Miller, A., et al. (2014). A semi-automated magnetic capture probe based DNA extraction and real-time PCR method applied in the Swedish surveillance of Echinococcus multilocularis in red fox (Vulpes vulpes) faecal samples. *Parasit Vectors* 7, 583.

Leigh, J. W., and Bryant, D. (2015). popart : full-feature software for haplotype network construction. *Methods Ecol Evol* 6, 1110–1116. doi: 10.1111/2041-210X.12410

Maksimov, P., Isaksson, M., Schares, G., Romig, T., and Conraths, F. J. (2019). Validation of PCR-based protocols for the detection of Echinococcus multilocularis DNA in the final host using the Intestinal Scraping Technique as a reference. *Food and Waterborne Parasitology* 15, e00044.

Nakao, M., Yokoyama, N., Sako, Y., Fukunaga, M., and Ito, A. (2002). The complete mitochondrial DNA sequence of the cestode Echinococcus multilocularis (Cyclophyllidea: Taeniidae). *Mitochondrion* 1, 497–509. doi: 10.1016/S1567-7249(02)00040-5

Toparslan, E., Karabag, K., and Bilge, U. (2020). A workflow with R: Phylogenetic analyses and visualizations using mitochondrial cytochrome b gene sequences. *PLoS One* 15, e0243927. doi: 10.1371/journal.pone.0243927
